# The endometrial transcriptomic response to pregnancy is altered in cows after uterine infection

**DOI:** 10.1371/journal.pone.0265062

**Published:** 2022-03-31

**Authors:** Mackenzie J. Dickson, Jeanette V. Bishop, Thomas R. Hansen, I. Martin Sheldon, John J. Bromfield

**Affiliations:** 1 Department of Animal Sciences, University of Florida, Gainesville, FL, United States of America; 2 Department of Biomedical Sciences, Colorado State University, Fort Collins, CO, United States of America; 3 Swansea University Medical School, Swansea, United Kingdom; Fondazione IRCCS Ca’ Granda Ospedale Maggiore Policlinico, ITALY

## Abstract

Pregnancy induces changes in the transcriptome of the bovine endometrium from 15 days after insemination. However, pregnancy is less likely to occur if cows had a postpartum bacterial infection of the uterus, even after the resolution of disease. We hypothesized that uterine bacterial infection alters the endometrial transcriptomic signature of pregnancy after the resolution of disease. To examine the endometrial transcriptomic signature of pregnancy, cows were inseminated 130 days after intrauterine infusion of pathogenic *Escherichia coli* and *Trueperella pyogenes*, subsequently endometrium was collected 16 days after insemination for RNA sequencing. We found 171 pregnancy regulated genes in cows 146 days after bacterial infection. When comparing our findings with previous studies that described the endometrial transcriptomic signature of pregnancy in healthy cows, 24 genes were consistently differentially expressed in pregnancy, including *MX1*, *MX2* and *STAT1*. However, 12 pregnancy regulated genes were found only in the endometrium of healthy cows, including *ISG15* and *TRANK1*. Furthermore, 28 pregnancy regulated genes were found only in the endometrium of cows following bacterial infection and these were associated with altered iNOS, TLR, and IL-7 signaling pathways. Although 94 predicted upstream regulators were conserved amongst the studies, 14 were found only in the endometrium of pregnant healthy cows, and 5 were found only in cows following bacterial infection, including AIRE, NFKBIA, and DUSP1. In conclusion, there were both consistent and discordant features of the endometrial transcriptomic signature of pregnancy 146 days after intrauterine bacterial infusion. These findings imply that there is an essential transcriptomic signature of pregnancy, but that infection induces long-term changes in the endometrium that affect the transcriptomic response to pregnancy.

## Introduction

All dairy cows have bacteria present in the uterus shortly after calving [1–3] and up to 40% of postpartum cows will develop a uterine disease including metritis or clinical endometritis [4]. Even after the resolution of uterine disease cow fertility is diminished due to reduced pregnancy rates, greater pregnancy loss, and a greater incidence of culling compared to cows that do not develop uterine disease [5, 6]. The persistent subfertility observed in cows after disease resolution suggests that the physiology of the reproductive tract is perturbed long-term by uterine disease [7]. When bred correctly, approximately 70–80% of cattle will achieve fertilization after insemination; however, up to 50% of these pregnancies will be lost within the first week of gestation with an addition 30% of pregnancies are lost during embryo elongation and maternal recognition of pregnancy between days 8 and 27 [8, 9].

Previous work has shown that experimental uterine infection reduces the ability of oocytes to develop to morula stage embryos after in vitro fertilization and culture, suggesting that oocyte competence is diminished during and after infection [10]. Interestingly, the transfer of embryos derived from healthy donors to recipients that previously had uterine disease does not rescue uterine disease associated pregnancy loss, suggesting the endometrium also plays a role in the subfertility observed in cows after uterine disease [6, 11, 12], suggesting additional pregnancy loss occurs after day 7 of pregnancy when embryos are transferred to recipients.

During active uterine infection the endometrium increases expression of inflammatory markers and alters prostaglandin secretion [13, 14]. Surprisingly, even after the clearance of infection, persistent changes to the transcriptome of granulosa cells, endometrium and oviduct tissues are apparent in cows months after uterine infection, demonstrating uterine infection imparts a long-term impact on reproductive tissues that may contribute to altered fertility of cows after resolution of uterine disease [15, 16]. In healthy cows, the endometrial environment during early pregnancy (days 15–17) is largely influenced by the secretion of interferon-tau (IFNT) by the conceptus which drives maternal recognition of pregnancy. Many interferon-inducible genes such as *ISG15*, *MX1*, *MX2*, *OAS1*, and *STAT1*, are upregulated in the endometrium of pregnancy in healthy cows compared to cycling cows at the time of maternal recognition of pregnancy [17–19]. However, it remains unclear which of these genes are essential for pregnancy, and whether the detrimental effects of uterine infection are evident in the endometrial transcriptome of pregnancy.

To disentangle the effects of age, lactation, and the postpartum period, we took advantage of nonlactating, primiparous cows that were subjected to experimental uterine infection with pathogenic bacteria [10]. We hypothesized that uterine bacterial infection alters the endometrial transcriptomic signature of pregnancy after the resolution of disease. Using endometrial tissues collected from a previous experiment [10], we evaluated the endometrial transcriptome of pregnant and non-pregnant cows 146 days after intrauterine infusion with pathogenic bacteria. We then used bioinformatics to compare the endometrial transcriptome of previously infected cows with the endometrial signature of pregnancy in healthy cows from previous studies. Finally, we verified the discrepancies between the infected and healthy cows using qPCR for selected genes from cows infused with bacteria or vehicle medium. We found that there is a consistent endometrial transcriptomic signature of pregnancy regardless of prior uterine infection. However, some pregnancy-associated genes were not altered in cows after uterine infection, while a cohort of unique pregnancy related genes were identified in cows after infection. The absence or presence of these defined genes may contribute to the subfertility observed in cows after uterine infection.

## Materials and methods

The University of Florida Institutional Animal Care and Use Committee approved all animal procedures (protocol number 201508884). The experiment was conducted from February to August 2018 at the University of Florida Dairy Research Unit. Methodology and data pertaining to intrauterine infusion, disease progression, ovarian function, developmental competence of oocytes after in vitro fertilization, and embryo quality have been previously reported [10]. All previous analysis was performed comparing vehicle infused cows with bacteria infused cows, regardless of pregnancy status [10]. Briefly, 23 two-year old primiparous non-lactating Holstein cows had estrous cycles synchronized prior to the intrauterine infusion of vehicle medium (*n* = 11) or *Escherichia coli* MS499 and *Trueperella pyogenes* MS249 (*n* = 12), designated as day 0 of the experiment. On experimental day 110, estrous cycles were synchronized and on experimental day 130, all cows were artificially inseminated with 500 μL of commercial semen from the sire Passat 7HO12659 (Select Sires, Plain City, OH). All cows were slaughtered by captive bolt followed by exsanguination at a USDA approved abattoir on experimental day 146, sixteen days after insemination to determine pregnancy status by flushing of the uterus. The uterine horn ipsilateral to the corpus luteum was opened and intercaruncular endometrium was dissected with sterile forceps and scissors, snap frozen in liquid nitrogen and stored at -80°C until processed for RNA sequencing. No cows had clinical signs (vaginal discharge, uterine pus, fever) of uterine infection at the time of insemination (day 130) or slaughter (day 146).

Serum progesterone and interferon tau (IFNT) content of uterine fluid was quantified by ELISA as previously reported [10] to verify the pregnancy status of cows. The presence of an embryo and uterine fluid concentration of IFNT defined whether each cow was pregnant or non-pregnant. In addition to the previous report of data [10], **S1 Table** shows the effect of pregnancy status on ovarian function, and developmental competence of oocytes after in vitro fertilization and culture.

### RNA extraction and cDNA library preparation

Intercaruncular endometrium was thawed and immersed in 350 μL RLT buffer (Qiagen, Hilden, Germany) and homogenized using 2.8 mm ceramic beads (Qiagen) in a bead beater tissue homogenizer (Precellys 24; Bertin Technologies SAS, Montigny-le-Bretonneux, France). Samples were processed using two cycles of 45 s each at 6500 rpm with a 30 s interval between cycles. After homogenization, endometrial RNA was purified using the RNeasy mini kit and on-column DNase digestion according to the manufacturer’s instructions (Qiagen). Total RNA was quantified, and RNA quality was assessed using an Agilent 2100 Bioanalyzer (Agilent Technologies, Santa Clara, CA). Only samples with a total RNA 28S:18S ratio > 0.5 and RNA integrity number ≥ 6.8 were used for library construction and RNA sequencing. Whilst it would have been ideal to include vehicle-infused pregnant as well as bacteria-infused endometrium in the analysis, the former RNA was of insufficient quality for RNA sequencing (RNA integrity number < 6.8). However, as the quality threshold is less stringent, we were able to use this RNA for qPCR to examine the expression of selected genes from the RNA sequencing in pregnant and non-pregnant animals after vehicle infusion, as well as after bacterial infusion and uterine infection. Library preparation was conducted by Novogene Inc. (Sacramento, CA) using NEBNext Ultra II RNA Library Prep Kit for Illumina (New England BioLabs Inc., Ipswich, MA). Barcoded libraries were assessed using Qubit 2.0 (ThermoFisher, Invitrogen, Grand Island, NY), Agilent 2100 Bioanalzyer (Agilent Technologies), and quantified with qPCR. Individual libraries were pooled at equal molar concentrations and sequencing was performed using the Illumina NovaSeq 6000 platform producing paired-end 150 base pair reads and Q30 >80%. All transcriptome analysis described from hereon was performed only using endometrium from pregnant (*n* = 3) and non-pregnant (*n* = 4) cows after intrauterine infusion of pathogenic bacteria.

### Read mapping and differential gene expression analysis

Original data files from high-throughput sequencing were transformed into sequenced raw reads by CASAVA base recognition and stored in FASTQ format files. Reads were filtered to remove adaptors, reads with more than 10% uncertain nucleotides, and reads with more than 50% low quality reads. After data filtering, paired-end clean reads of each sample were aligned to the latest bovine reference genome (ARS-UCD1.2) using HISAT2 v2.1 and quantification was performed using FeatureCounts v1.5.0. Differential expression analysis between pregnant and non-pregnant cows was performed using the DESeq2 R package v2_1.6.3. The resulting *P* values were adjusted using the Benjamini and Hochberg’s approach for controlling the False Discovery Rate (FDR). Genes with an FDR ≤ 0.05 were considered as differentially expressed between pregnant and non-pregnant cows. The data underlying this article are available in NCBI Gene Expression Omnibus (GEO) at https://www.ncbi.nlm.nih.gov/geo/query/acc.cgi?acc=GSE183149, and can be accessed with the series identifier GSE183149.

### Pathway analysis of differentially expressed genes

Ingenuity Pathway Analysis (Qiagen) was used to identify canonical pathways, gene networks, and upstream regulators of differentially expressed genes affected by pregnancy [20]. Differentially expressed genes with an FDR ≤ 0.05 were used for analysis. Canonical pathways with a -log_10_ *P* ≥ 1.3 and corresponding z-score to predict activation (z ≥ 2) or inactivation (z ≤ -2) were identified. A network score of z ≥ 2 gives 99% confidence the network was not identified by chance. Gene networks were identified by assessing the number of differentially expressed genes in each network. Predicted upstream regulators of differentially expressed genes were limited to genes, RNAs, and protein, and predicted diseases and functions, were identified by z-scores ≥ 2 or ≤ -2 and were considered significant predictors of activation or inhibition of differentially expressed genes, respectively.

### Endometrial transcriptome data sets from comparison papers

To determine differences in the pregnancy transcriptome signature between healthy and previously infected cows, results of the experiment presented here were compared to the data from other transcriptome analyses describing the endometrial signature in healthy cows. We selected three studies that analyzed bovine endometrium from day 15 [17], day 16 [19], or day 17 [18] of pregnancy compared to the corresponding day of the estrous cycle in cows that were not inseminated. Briefly, the studies synchronized estrous cycles of animals and inseminated a portion of cows with semen, with the remaining cows not inseminated [18, 19] or inseminated with sperm-free supernatant [17]. The studies only considered cows to be pregnant when a conceptus was recovered. Gene lists were obtained from published supplemental tables and gene identities verified. Gene expression data from Bauersachs et al., and Cerri et al., were obtained from microarray (Affymetrix) and published supplemental tables included NCBI gene identification numbers. Sequencing data from Forde et al., were identified with Ensembl transcript identification numbers and were converted to NCBI gene identification numbers using Ensembl biomart with database Ensembl Genes 100 and the bovine genome ARS-UCD1.2 [21]. Any Ensembl transcript identification that was not automatically detected and converted, was manually identified using UniProt accession numbers, or RefSeq mRNA accession numbers provided in supplemental tables from the original publication. NCBI gene identification numbers from all datasets were verified using bioDBnet [22]. Only confirmed gene identification numbers that were current and identified a single gene were used for our analysis. Genes were considered differentially expressed and used for comparison against the current data if they had an FDR ≤ 0.05 and had log_2_ fold-change ≥ 1.5 or ≤ -1.5 (**S2–S4 Tables**). We believe this approach would yield the most robust identification of differentially expressed genes in the three previously described experiments using different platforms and experimental models.

Each data set was analyzed independently using Ingenuity Pathway Analysis (Qiagen) to identify canonical pathways, gene networks, and predicted upstream regulators of defined differentially expressed genes. The same threshold values described above were applied to these datasets, with canonical pathways significant if -log_10_ *P* ≥ 1.3 and upstream regulators if z-scores ≥ 2 or ≤ -2.

### qPCR

We used qPCR to quantify conserved and unique pregnancy-associated genes in pregnant and non-pregnant cows previously infused with pathogenic bacteria or vehicle medium. Reverse transcription was performed using the Verso cDNA synthesis kit (Thermo Fisher Scientific, Waltham, MA). All primers were designed using the National Center for Biotechnology Information (NCBI) database (**S5 Table**). Amplification efficiency for each primer pair was evaluated and met MIQE guidelines [23]. Real time RT-PCR was performed in duplicate 20 μL reactions including forward and reverse primer, iTaq Universal SYBR green master mix (Bio-Rad, Hercules, CA) and 2 to 40 ng cDNA. A CFX Connect light cycler (Bio-Rad) was used with an initial denaturation step at 95°C for 30 sec followed by 40 cycles of a two-step protocol using 95°C for 5 s and annealing and extension at 60°C for 30 s. The primer set for *OXTR* required a three-step protocol using 63°C as annealing temperature for 5 s and extension at 60°C for 30 s. A no template control was used to determine non-specific amplification for each primer pair. Relative expression for each gene was calculated using the 2^-ΔCt^ method relative to the geometric mean of the selected housekeeping genes (*ACTB*, *GAPDH*, *RPL19*). Housekeeping gene expression was stable across treatments and pregnancy status (*P* > 0.05).

### Statistical and data analyses

The experimental unit was the cow and, unless otherwise stated, data were analyzed with two main factors to investigate the effects of treatment (infusion of vehicle vs. infusion of bacteria), pregnancy status (non-pregnant vs pregnant), and the interaction between treatment and pregnancy. Oocyte number, morulae number, morulae development rate, concentration of peripheral progesterone, corpus luteum diameter, and IFNT data were analyzed with general linear model using effects of treatment, pregnancy status, and the interaction between treatment and pregnancy status (SPSS v26; IBM Corporation, Armonk, NY). Endometrial qPCR data were log transformed for normality and data analyzed using a general linear model with the effects of treatment, pregnancy status, and the interaction of treatment and pregnancy status. Pairwise comparisons were used to analyze the interaction term. Transcriptome data of all transcripts were used for principal component analysis using ClustVis [24]. Genes that were differentially expressed in non-pregnant compared to pregnant cows were selected using an FDR ≤ 0.05 and are reported as log_2_ fold-change. A heat map was generated for differentially expressed genes with Heatmapper [25] using Pearson distance measurement method and complete linkage clustering. GraphPad Prism v8.4 was utilized for linear regression analysis comparing RNA sequencing data with qPCR results.

## Results

### Pregnancy alters the endometrial transcriptome after intrauterine infusion of bacteria

Cows received a single intrauterine infusion of pathogenic bacteria to induce a uterine infection and allowed time to recover. Following uterine infection and resolution (130 d), cows were inseminated and 16 days later, uterine contents were collected. A total of 12 filamentous conceptuses (vehicle = 6 of 11, bacteria = 6 of 12) were recovered 16 days following insemination. Interferon tau was detected in 17 of 23 uterine fluid samples (vehicle = 8 of 11, bacteria = 9 of 12) and ranged from 0.086 to 6,858 ng/mL and was greater than 61 ng/mL in all cows from which an embryo was recovered. As such, 5 vehicle infused cows and 5 bacteria infused cows were designated non-pregnant, 6 vehicle infused cows and 7 bacteria infused cows were designated as pregnant based on the presence of an embryo and uterine fluid concentration of IFNT (**S1 Table**). Retrospective analysis of data previously reported [10] showed there was no difference in the developmental potential of oocytes collected from pregnant or non-pregnant cows (*P* > 0.05; **S1 Table**). This data confirmed that any observed differences in pregnancy status would not be due to ovarian function (CL size or progesterone) or oocyte competence (embryos development after IVF and culture). As such, the endometrial transcriptomes were sequenced to characterize the endometrial transcriptomic signature of pregnancy after uterine infection.

After sequencing and read processing of intercaruncular endometrium, 445,762,946 high quality reads were used for analysis (approximately 63.7 million clean reads per sample). An average of 95.7% high quality reads were aligned to the reference genome resulting in the detection of 26,092 unique transcripts in the endometrium (**S6 Table**). The most abundant endometrial genes identified based on total read counts are described in **S7 Table**. Principal component analysis using the expression of all transcripts of pregnant and non-pregnant cows explains 29% and 23.8% of the variance observed, with no distinct clustering of pregnant and non-pregnant transcriptomes after intrauterine infusion of pathogenic bacteria (**S1 Fig**). RNA sequencing reads and gene expression measured by qPCR of three target genes (*CPM*, *OXTR*, and *STC2*) revealed an r^2^ > 0.85 suggesting a robust linear relationship between sequencing data and targeted PCR amplification (**S2 Fig**).

**Fig 1:**
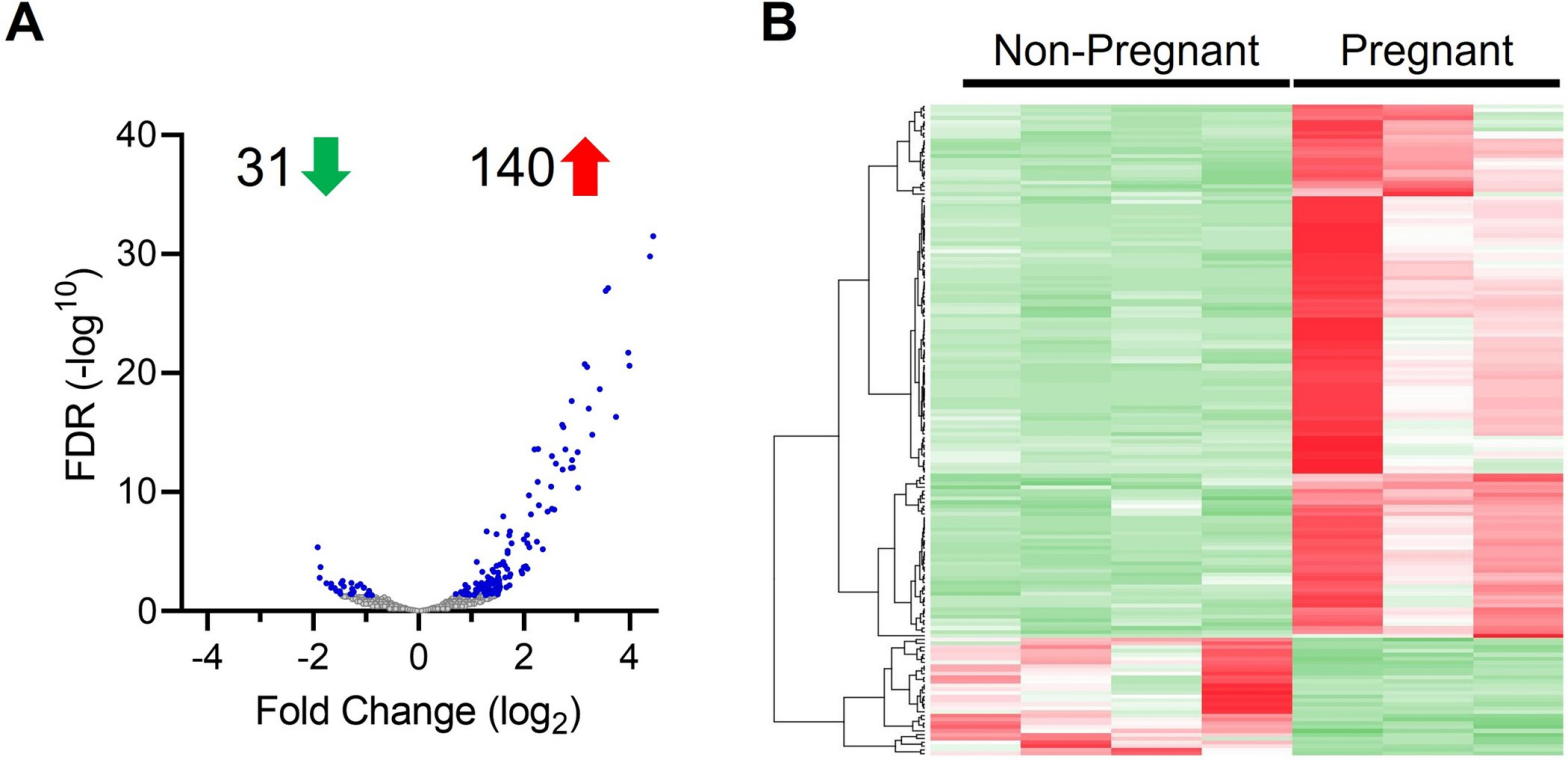
Pregnancy regulated genes following intrauterine infusion of pathogenic bacteria. Cows were inseminated 130 days after intrauterine infusion of pathogenic bacteria and endometrium was collected 16 days later. Based on the presence of an embryo and interferon tau, cows were designated as pregnant (n = 3) or non-pregnant (n = 4). Intercaruncular endometrium was subjected to RNA sequencing analysis. (A) Volcano plot depicting the fold change (log2) and false discovery rate (-log10 FDR) of each endometrial gene of pregnant cows compared to non-pregnant cows. Differentially expressed genes (FDR < 0.05) are colored blue. A total of 140 genes were upregulated and 31 genes were downregulated in the endometrium of pregnant cows compared to non-pregnant cows. (B) Heatmap presents hierarchal clustering of differentially expressed genes with each column representing a single cow and each row a single gene. Rows were clustered with Pearson distance measurement method and complete linkage. Gene expression intensities are shown in green (decreased) to red (increased).

After uterine infection, a total of 171 pregnancy regulated genes were identified in the intercaruncular endometrium 16 days after insemination (FDR ≤ 0.05; **Fig 1A** and **S8 Table**). Of the 171 pregnancy regulated genes, 140 genes were upregulated and 31 were downregulated in pregnant cows compared to non-pregnant cows. A heatmap shows the uniform expression of the 171 pregnancy regulated genes identified in the intercaruncular endometrium of cows after uterine infection (**Fig 1B**).

**Fig 2:**
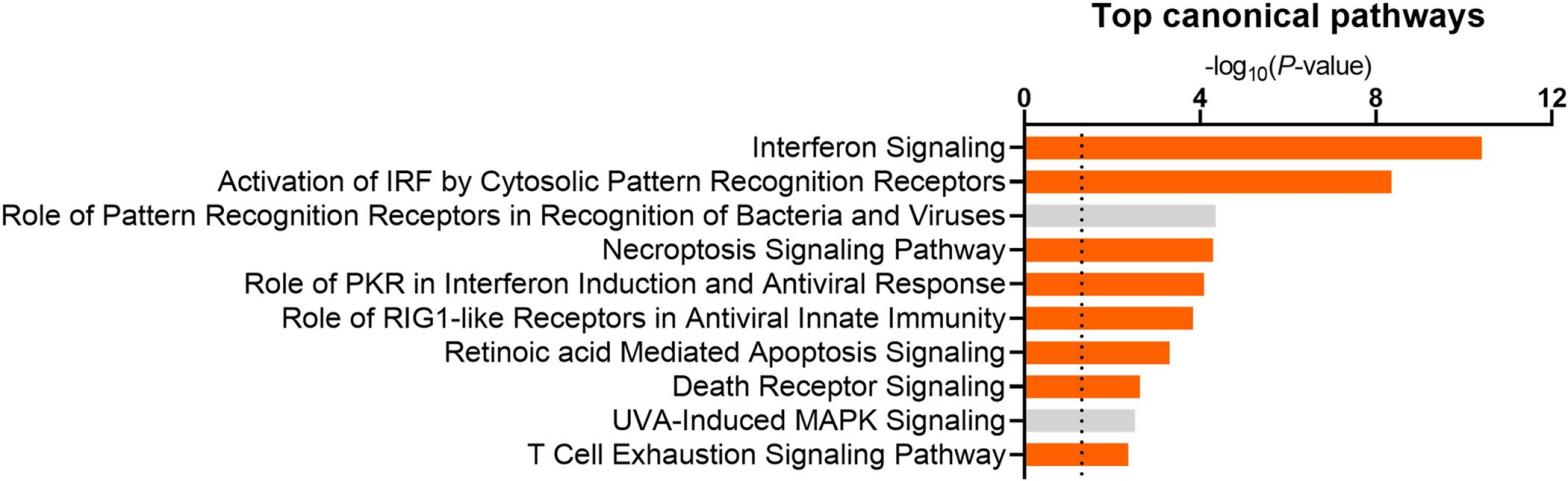
Canonical pathways regulated by pregnancy in cows after intrauterine infusion of pathogenic bacteria.

Analysis of the 171 pregnancy regulated genes identified 23 canonical pathways altered in the endometrium of cows after uterine infection (*P* < 0.05; **Fig 2** and **S9 Table**). The most significant canonical pathways included 1) interferon signaling; 2) activation of IRF by cytosolic pattern recognition receptors; 3) role of pattern recognition receptors in recognition of bacteria and viruses; and 4) necroptosis signaling pathway.

The top 10 altered canonical pathways were identified using IPA based on 171 differentially expressed genes characterized in the endometrium of pregnant cows compared to non-pregnant cows after infusion with bacteria. Pathways were considered significant if -log_10_ *P* ≥ 1.3, depicted by the dotted line. Most pathways were predicted to be activated (orange, z-score ≤ -2). Grey bars represent significantly affected canonical pathways where a z-score could not be calculated. A full list of altered canonical pathways can be found in **S9 Table**.

Using the 171 pregnancy regulated genes identified in the endometrium after uterine infection, ten gene networks were differentially regulated (*P* < 0.05) including 1) connective tissue disorders, immunological disease, inflammatory disease; 2) dermatological diseases and conditions, immunological disease, organismal injury and abnormalities; and 3) antimicrobial response, infectious diseases, inflammatory response (**S10 Table**).

A total of 119 genes, RNAs, or proteins were identified as predicted upstream regulators of pregnancy regulated genes identified in the endometrium after uterine infection, of which 83 were activated and 36 were inhibited (*P* < 0.05; **S11 Table**). The top three upstream regulators predicted to be activated in the endometrium of pregnant cows after uterine infection include 1) IFNG (cytokine); 2) IFNA2 (cytokine); and 3) PRL (cytokine). The top three upstream regulators predicted to be inhibited in the endometrium of pregnant cows after uterine infection include 1) MAPK1 (kinase); 2) NKX2-3 (transcription regulator); and 3) IL1RN (cytokine).

### Consistent transcriptomic features of pregnancy in healthy and infected endometrium

**Fig 3:**
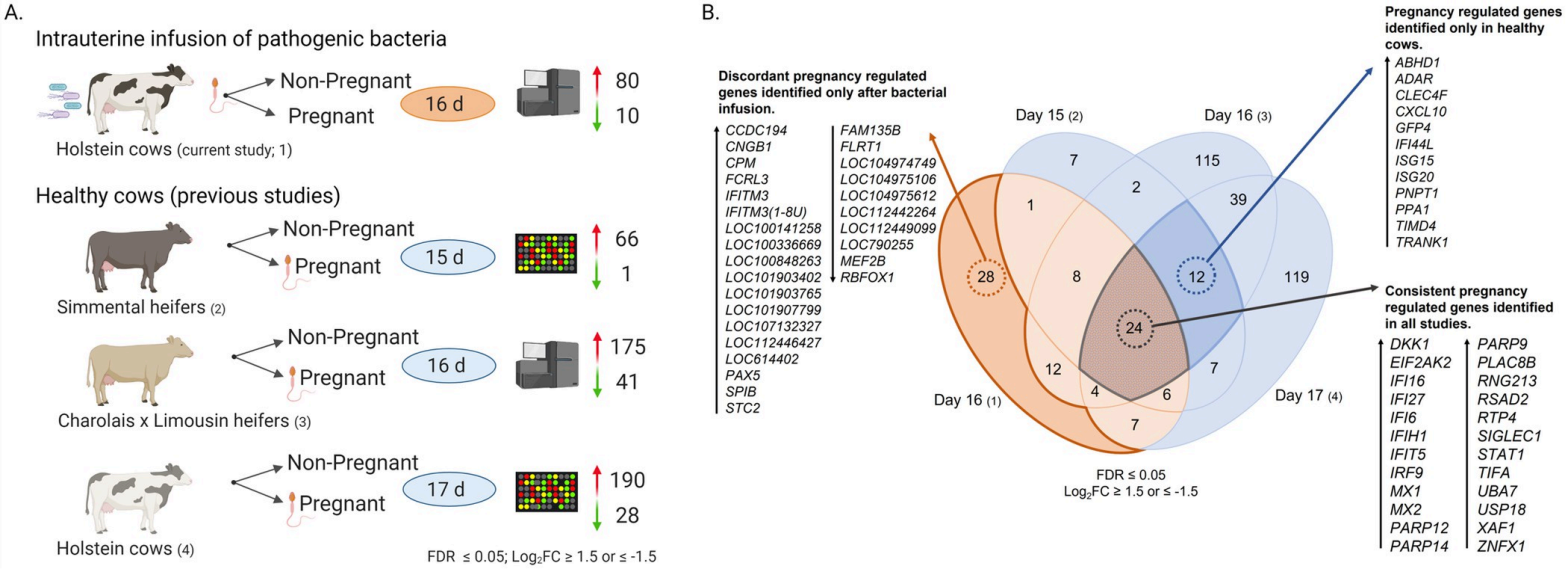
Consistent and discordant pregnancy regulated genes in the endometrium of healthy cows and cows following intrauterine infusion of pathogenic bacteria. Data from previously published studies using healthy cows were compared to the current data from cows after intrauterine infusion of bacteria. (A) Differentially expressed genes with an FDR ≤ 0.05 and log_2_ FC ≥ 1.5 or ≤ -1.5 from all studies were considered. Holstein cows were inseminated 130 days after intrauterine infection of pathogenic bacteria and sixteen days later classified as non-pregnant or pregnant based on the presence of a conceptus and IFNT (orange). Three previous studies were used for comparison (blue), including 1) microarray analysis of Simmental heifers either inseminated with sperm-free supernatant and classified as non-pregnant cycling or inseminated and classified as pregnant based on presence of a conceptus fifteen days later [17]; 2) RNA sequencing of crossbred Charolais and Limousin heifers either never inseminated and classified as non-pregnant cycling, or inseminated and classified as pregnant based on presence of a conceptus sixteen days later [19]; and 3) microarray analysis of Holstein cows either not inseminated and classified as non-pregnant cycling, or inseminated and classified as pregnant based on the presence of a conceptus seventeen days later [18]. Comparisons show differentially expressed genes in the pregnant cows compared to the non-pregnant cows. (B) A Venn diagram displays the overlap of differentially expressed endometrial genes in all four studies. The orange segment represents the current study in cows after infusion of bacteria, and the blue segments represent previous studies using healthy cows. Arrows depict if genes were upregulated or downregulated in the endometrium of pregnant cows compared to non-pregnant cows. A complete list of differentially expressed genes from all studies used here can be found in **S2–S4** and **S12 Tables**, and a list with details of genes listed in the figure can be found in **S13 Table**.

Three previous studies report the effect of pregnancy on the endometrial transcriptome of healthy cows at the time of maternal recognition of pregnancy (day 15, 16 and 17 post insemination). All three of the previous studies reported the transcriptomic signature of pregnancy in healthy cows [17–19]. Subsequently the transcriptomic signature of pregnancy in healthy cows was compared to our findings using cows with an experimentally induced uterine infection. The goal was to identify consistent and discordant features of the endometrial transcriptomic signature of pregnancy after uterine infection. Data from all studies were restricted to differentially expressed genes with a more stringent threshold (FDR ≤ 0.05 and log_2_FC ≥ 1.5 or ≤ -1.5; **Fig 3A**). The differentially expressed genes identified in the endometrium of pregnant cows after uterine infection were modified to the same stringent criteria (FDR ≤ 0.05 and log_2_FC ≥ 1.5 or ≤ -1.5), restricting analysis to 90 differentially expressed genes from the original 171 previously discussed (**S12 Table**). To summarize, a total of 67, 216 and 218 genes were regulated by pregnancy in healthy cows at the corresponding day of the estrous cycle (day 15, day 16 and day 17, respectively; **Fig 3A**). A complete list of genes regulated by pregnancy in healthy cows is provided in **S2–S4 Tables**. When comparing all four studies, a total of 24 genes were consistently upregulated by pregnancy regardless of previous uterine infection, including *DKK1*, *MX1*, *MX2*, *STAT1* and a number of interferon stimulated genes (**Fig 3B** and **S13 Table**).

### Distinct transcriptomic features of pregnancy in healthy and post-infection cows

While there were consistent features of the endometrial transcriptomic signature of pregnancy across all four studies, there were also discordant features associated with uterine infection. A total of 12 pregnancy regulated genes were upregulated only in healthy cows and not cows after uterine infection, including *CXCL10*, *ISG15* and *TRANK1* (**Fig 3B**). Furthermore, 28 unique pregnancy regulated genes were only identified in cows following uterine infection and not in healthy cows (**Fig 3B** and **S13 Table**). Of the 28 unique pregnancy regulated genes in cows after uterine infection, 10 genes were downregulated, and 18 genes were upregulated. Five of these genes encode for non-coding RNAs, 2 are pseudogenes and 3 are uncharacterized proteins (**Fig 3B**). These discordant pregnancy regulated genes identified in cows after a uterine infection may provide insight into the long-term impact and altered fertility following resolution of uterine infection.

### Predicted pathways and regulators differ between healthy and post-infection cows

**Fig 4:**
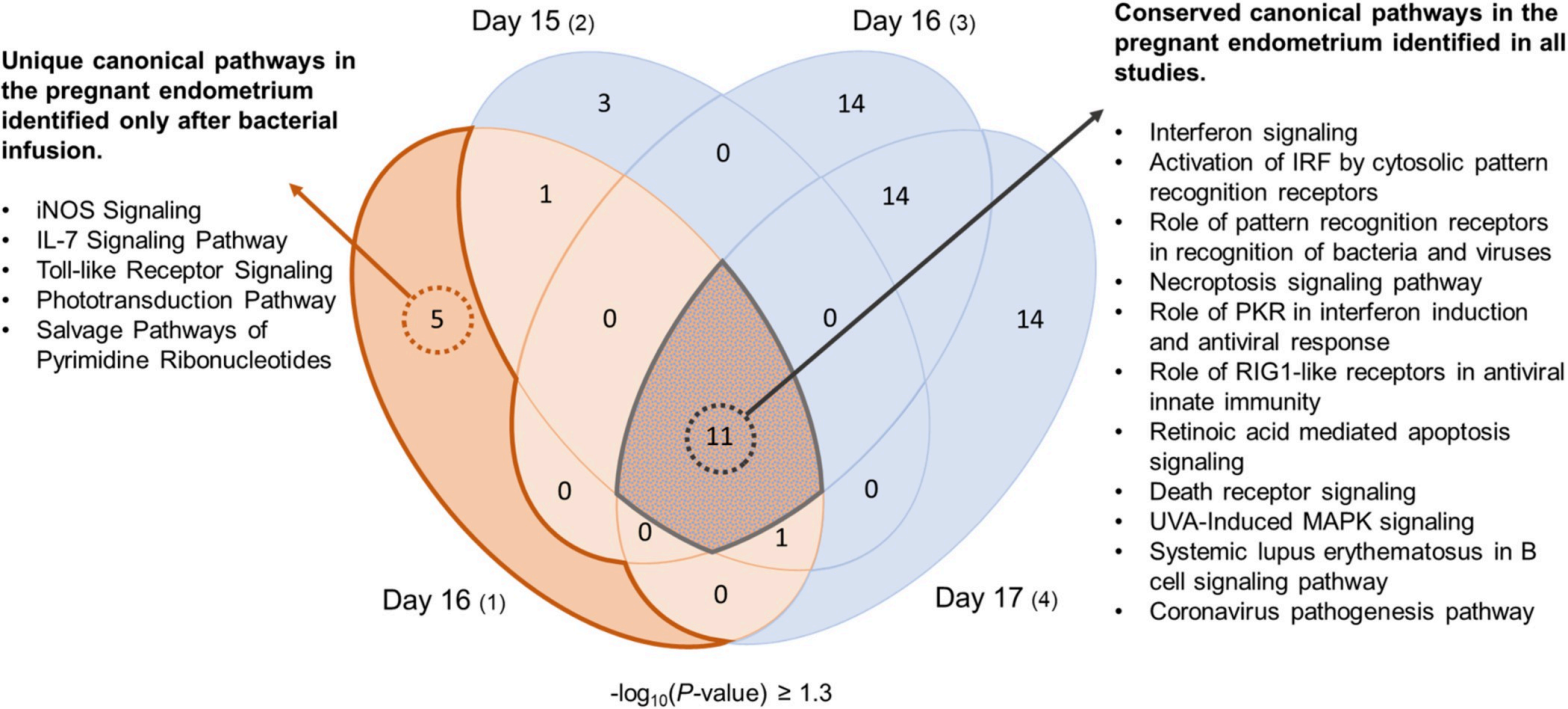
Pregnancy regulated canonical pathways in the endometrium of healthy cows and cows following intrauterine infusion of pathogenic bacteria.

Using the criteria described above, pregnancy regulated genes from each study were analyzed to further identify dysregulated canonical pathways and predicted upstream regulators of differentially expressed genes. A total of 18 canonical pathways were affected at day 16 of pregnancy in cows following uterine infection (*P* < 0.05; **Fig 4**). In comparison, a total of 16, 39 and 40 canonical pathways were affected at day 15, day 16 and day 17, respectively, in healthy cows (*P* < 0.05; **Fig 4** and **S14 Table**). A total of 11 canonical pathways were consistently affected by pregnancy amongst the four studies, including 1) interferon signaling, 2) activation of IRF by cytosolic pattern recognition receptors, and 3) role of pattern recognition receptors in recognition of bacteria and viruses (*P* < 0.05; **Fig 4**). Interestingly, five discordant canonical pathways were uniquely affected by pregnancy following uterine infection, including iNOS signaling, Toll-like receptor signaling, and IL-7 signaling pathway (*P* < 0.05; **Fig 4**). There were no canonical pathways affected by pregnancy that were only identified in healthy cows and not in cows following uterine infection.

Data from previously published studies using healthy cows were compared to the current data from cows after infusion with bacteria. Altered canonical pathways were determined using the differentially expressed genes identified in the endometrium of non-pregnant cows compared to pregnant cows presented in **Fig 3** and **S13 Table**. Canonical pathways were considered significant if -log_10_ (*P* value) ≥ 1.3. The orange segment represents the current study in cows after infusion of bacteria, and the blue segments represent previous studies using healthy cows. A complete list of altered canonical pathways, z-score, *P*-value and associated genes from all studies can be found in **S14 Table**.

**Fig 5:**
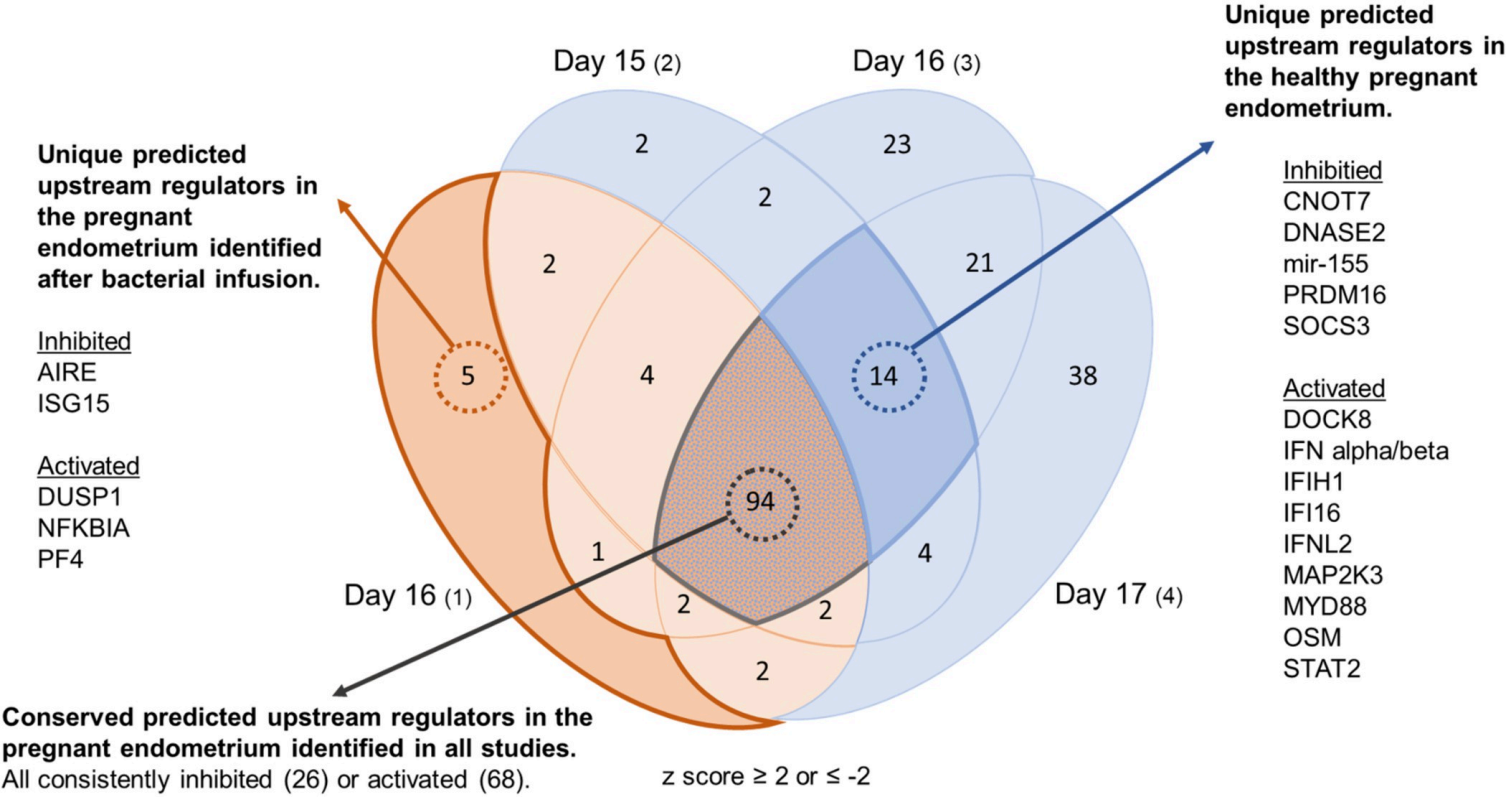
Pregnancy regulated predicted upstream regulators in the endometrium of healthy cows and cows following intrauterine infection of bacteria.

A total of 112 predicted upstream regulators of pregnancy regulated genes were identified in cows following uterine infection (*P* < 0.05; **Fig 5** and **S15 Table**). In comparison, a total of 124, 161 and 177 predicted upstream regulators of pregnancy regulated genes were identified in healthy cows at day 15, day 16 and day 17, respectively (*P* < 0.05; **Fig 5** and **S15 Table**). In total, 94 predicted upstream regulators of pregnancy regulated genes were consistently identified in all four studies, including the activation of IFN, IL-1β, NFκB and PRL, and the inhibition of ACKR2, IL-1RN, and IL-10RA (*P* < 0.05; **Fig 5** and **S15 Table**). A total of 14 predicted upstream regulators were identified only in healthy cows (*P* < 0.05; **Fig 5**), while five unique predicted upstream regulators were identified only in cows after uterine infection, including the inhibition of ISG15 and AIRE, and activation of DUSP1, NFKBIA, and PF4 (*P* < 0.05; **Fig 5**). Examples of gene networks associated with predicted upstream regulators of pregnancy regulated genes following uterine infection are shown in **S3 Fig**.

Data from previously published studies using healthy cows were compared to the current data from cows after infusion with bacteria. Significant predicted upstream regulators of differentially expressed endometrial genes (presented in **Fig 3** and **S13 Table**) had a z-score ≥ 2 or ≤ -2. The orange segment represents the current study in cows after infusion of bacteria, and the blue segments represent previous studies using healthy cows. A complete list of predicted upstream regulators, associated genes, activation status, z-score and *P*-value from all studies can be found in **S15 Table**.

### Selected pregnancy regulated genes in the endometrium after uterine infection

**Fig 6:**
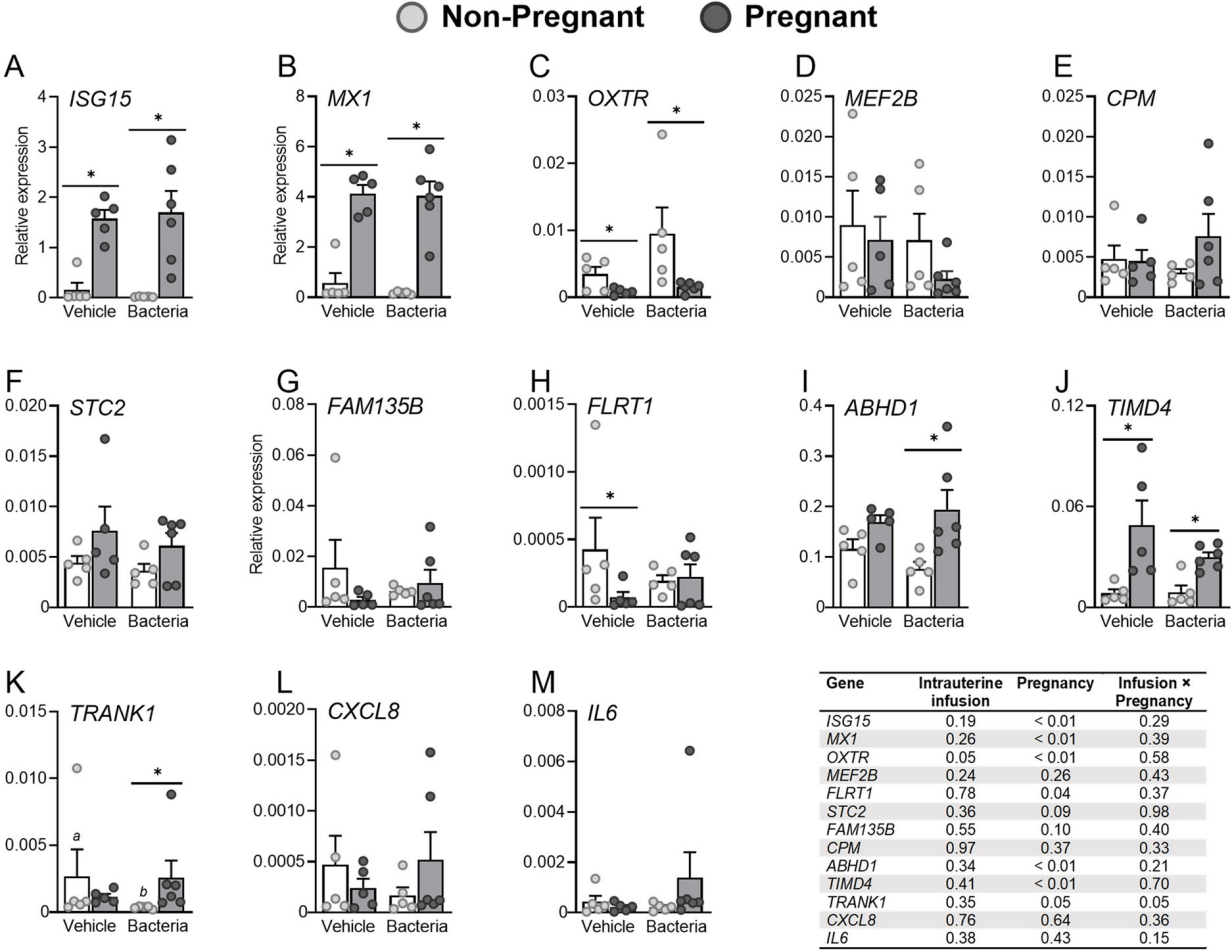
Effect of pregnancy and intrauterine infusion of pathogenic bacteria on the expression of endometrial genes identified by transcriptome analysis.

Following analysis to determine the endometrial transcriptomic signature of pregnancy, qPCR was performed on endometrium from cows that received an intrauterine infusion of either vehicle or pathogenic bacteria that were pregnant or non-pregnant (**Fig 6**).

Cows were inseminated 130 days after intrauterine infusion of either vehicle medium or pathogenic bacteria. Endometrium was collected 16 days later and based on the presence of an embryo and IFNT cows were designated as vehicle-non-pregnant (n = 5), vehicle-pregnant (n = 5), bacteria-non-pregnant (n = 5), or bacteria-pregnant (n = 6). Endometrial expression of (A) *ISG15*, (B) *MX1*, (C) *OXTR*, (D) *MEF2B*, (E) *CPM*, (F) *STC2*, (G) *FAM135B*, (H) *FLRT1*, (I) *ABHD1*, (J) *TIMD4*, (K) *TRANK1*, (L) *CXCL8*, and (M) *IL6* was evaluated by real-time RT-PCR. Data are presented as expression relative to the geometric mean of three housekeeping genes (*ACTB*, *GAPDH*, and *RPL19*). Each dot represents a cow, and the bar represents the mean ± SEM. * indicates *P* ≤ 0.05 between pregnant and non-pregnant within an infusion group. Superscripts indicate *P* ≤ 0.05 between treatment groups within a pregnancy status.

Expression of interferon-stimulated genes *ISG15* and *MX1* (known to be upregulated by pregnancy) were increased in the endometrium of pregnant cows compared to non-pregnant cows, regardless of intrauterine infusion (*P* ≤ 0.01; **Fig 6A and 6B**). Expression of *OXTR* (known to be downregulated by pregnancy) was decreased in the endometrium of pregnant cows compared to non-pregnant cows (*P* ≤ 0.01) and was increased by intrauterine infusion of pathogenic bacteria regardless of pregnancy (*P* = 0.05; **Fig 6C**).

Of the 28 unique, discordant genes regulated by pregnancy in cows following intrauterine infusion of pathogenic bacteria (**Fig 3B**), five genes that are not regulated by type 1 interferons (*MEF2B*, *CPM*, *STC2*, *FAM135B*, and *FLRT1*) were selected for analysis by qPCR. Expression of *MEF2B*, *CPM*, *STC2*, and *FAM135B* were not affected by pregnancy or intrauterine infusion (**Fig 6D–6G**); however, expression of *FLRT1* was affected by pregnancy, specifically in cows following intrauterine infusion of vehicle medium (*P* ≤ 0.05; **Fig 6H**).

Of the 12 genes consistently upregulated by pregnancy in healthy cows but not in cows following intrauterine infusion of pathogenic bacteria (**Fig 4B**), three genes (*ABHD1*, *TIMD4*, and *TRANK1*) were selected for analysis by qPCR (**Fig 6I–6K**). Endometrial expression of *ABHD1*, *TIMD4*, and *TRANK1* were affected by pregnancy (*P* ≤ 0.05) but not intrauterine infusion. Specifically, expression of *ABHD1* was increased by pregnancy only in cows following intrauterine infusion of bacteria (**Fig 6I**), while expression of *TIMD4* was increased by pregnancy regardless of intrauterine infusion (**Fig 6J**). In addition to being affected by pregnancy status, the expression of *TRANK1* was also affected by the interaction of intrauterine infusion and pregnancy (*P* = 0.05; **Fig 6K**), as expression was reduced by intrauterine infusion of pathogenic bacteria in non-pregnant cows when compared to non-pregnant cows infused with vehicle (*P* ≤ 0.05). Endometrial expression of the inflammatory mediators, *CXCL8* or *IL6*, were not affected by pregnancy or intrauterine infusion (*P* > 0.05; **Fig 6L and 6M**).

## Discussion

Although some of the subfertility following postpartum uterine disease is associated with compromised oocytes [10], it has been suggested that there may also be long-term impacts of uterine infection on the endometrium since transfer of embryos from healthy donors to recipients with prior uterine disease does resolve associated subfertility [6, 11, 12]. While our experimental model was not designed to detect differences in pregnancy rates after uterine infection, we found evidence for detrimental effects of uterine infection 146 days earlier on the endometrial transcriptome of pregnancy. The data presented in **S1 Table** also confirms that cows did not fail to become pregnant due to oocyte or ovarian dysfunction, allowing for a clear interpretation of transcriptome data acquired from pregnant and non-pregnant cows. As such, by comparing the endometrial transcriptome of pregnancy at the time of maternal recognition of pregnancy following bacterial infusion with data from previous studies using healthy cows, we found 24 genes and 11 pathways that were consistently upregulated at the time of maternal recognition of pregnancy, regardless of previous uterine infection. We suggest that this ensemble of genes and pathways comprises an endometrial signature of pregnancy. Other pregnancy-associated genes altered after uterine infection may contribute to subfertility associated with uterine disease.

Bovine maternal recognition of pregnancy is driven by IFNT secreted from the conceptus between days 15–17 of gestation and is a significant window of pregnancy loss in cattle [8, 9, 26]. While the three data sets describing the endometrial transcriptome of pregnancy performed by others uses different breeds of cattle, time points and analytic methods than what was used in the current study we believe the collective characterization of the endometrial transcriptome of pregnancy described here demonstrates an extremely robust, conserved set of genes required for pregnancy in all cattle. The collective data from the analysis described here show that many of the conserved genes identified across all four studies include interferon-inducible genes (*EIF2AK2*, *IFI6*, *IFI16*, *IFI27*, *IFIH1*, *IFIT5*, *MX1*, *MX2*, *STAT1*, *XAF1*). Consistently upregulated pathways in the pregnant endometrium across all studies, included interferon signaling, activation of interferon regulatory factor, role of pattern recognition receptors in recognition of bacteria and viruses, and necroptosis signaling pathway. These findings imply that core IFNT signaling in the endometrium is not impaired after uterine infection and that there is an essential transcriptomic signature of pregnancy. However, based on the transcriptome analysis several unique pregnancy regulated genes were only identified in healthy cows, including interferon-induced genes (*IFI44L*, *ISG20*, *IFNAR1*, *IFNAR2*) and interferon-independent genes (*ABHD1*, *TIMD4*, *TRANK1*). Conversely, several pregnancy regulated genes, canonical pathways, and predicted upstream regulators were only found in cows following uterine infection. These discordant, pregnancy regulated genes and pathways may contribute to subfertility following uterine disease or compromise later stages of pregnancy, and they warrant further investigation.

Establishment and maintenance of pregnancy requires maternal immune tolerance of the semi-allogeneic conceptus coordinated by a unique repertoire of immune cells. Interleukin (IL)-7 signaling is critical for development and function of T cells [27]. Interestingly, IL-7 signaling was identified as a discordant pregnancy affected pathway in cows following uterine infection that was not identified in healthy cows (**Fig 4** and **S14 Table**). Dysregulated IL-7 influences pregnancy outcomes in women, with altered *IL7* and *IL7R* expression in the decidua of women with recurrent miscarriage [28], and reduced peripheral IL-7 in obese pregnant mothers that are sub-fertile [29]. Conversely, exogenous administration of IL-7 during early pregnancy causes fetal resorption and decreased *Foxp3* gene expression in the decidua of mice that is indicative of T regulatory cells [28]. While the role of IL-7 signaling in bovine pregnancy warrants further investigation, altered IL-7 signaling in the endometrium may be impacting immune cell dynamics and reducing maternal immune tolerance of the conceptus in cows after uterine disease.

The transcription factor, autoimmune regulator (AIRE), was identified as an inhibited discordant upstream regulator of pregnancy regulated genes following uterine infection. Pregnancy regulated genes downstream of AIRE in cows following uterine infection are all upregulated (*EIF2AK2*, *IFI44*, *HERC6*, *PARP14*, *TNFSF10*), suggesting the dysregulated expression of these genes could be important for pregnancy establishment following uterine disease. Indeed, the expression of endometrial *AIRE* has been shown to be increased in highly fertile cows compared to less fertile cows [30], while uterine knockdown of *Aire* in mice inhibits embryo implantation [31]. The role of AIRE as a potential mediator of pregnancy establishment and fertility in cows following uterine infection warrants further exploration.

Establishment and maintenance of pregnancy requires a balance of pro- and anti-inflammatory processes that when dysregulated can be detrimental to pregnancy success [32]. Nitric oxide (NO) is an immune regulator with an important role during implantation and pregnancy [33, 34]. The inducible nitric oxide synthase (iNOS) pathway, regulated by *NOS2* expression, was identified as a discordant pregnancy regulated canonical pathway in cows following uterine infection and not in healthy cows. Peritoneal macrophages in women with endometriosis have increased *NOS2* expression and NO production compared to healthy women, which can be further exacerbated after LPS stimulation [35, 36], and in mice elevated NO inhibits embryo implantation [37]. Cows in the current study and cows diagnosed with a uterine infection are exposed to LPS from Gram-negative *E*. *coli* in the uterus that could potentially alter endometrial nitric oxide production and iNOS signaling, resulting in reduced fertility. However, the long-term effects of uterine infection on the endometrial transcriptome, and the use of congruent signaling pathways for both immunity and conception, once again highlight the importance of being able to avoid, resist, and tolerate infection with pathogenic bacteria in the postpartum uterus [38].

Toll-like receptors (TLR) respond to pathogen-associated molecular patterns and initiate a cellular signaling cascade that culminates in an innate immune response and production of pro-inflammatory cytokines [39]. Data here show that TLRs 2 to 10 are expressed in the bovine endometrium, and expression is not affected by pregnancy. However, the TLR signaling pathway was identified as a discordant pregnancy regulated canonical pathway in cows following uterine infection but not in healthy cows. In vitro activation of TLR signaling in human stroma cells of the early pregnant decidua can decrease the proportion of regulatory T cells in peripheral lymphocytes [40], suggesting that dysregulated TLR signaling is associated with pregnancy pathology [41]. Interestingly, bovine endometrial epithelial cells increase production of inflammatory cytokines in response to sperm via the TLR2/TLR4 pathways [42], which may explain why we observed changes in cows following uterine infection that were all inseminated, opposed to observation made in healthy cows where cycling cows that were never inseminated were used as the baseline comparison for pregnancy [17–19]. Numerous studies have described the acute activation of bovine endometrial TLR signaling in response to bacteria or bacterial components; however, it is unclear if alterations to TLR signaling persist in the reproductive tract after the resolution of uterine infection that could compromise pregnancy success [43, 44].

Nuclear factor kappa B inhibitor alpha (NFKBIA), which encodes for IκBα, is involved in both iNOS and TLR signaling pathways and was identified as a discordant, activated upstream regulator of pregnancy regulated genes in cows following uterine infection and not in healthy cows. While there is no direct evidence for a role of endometrial *NFKBIA* in fertility, *NFKBIA* expression and phosphorylation of IκBα are reduced in the endometrium of endometriosis patients with reduced fertility [45]. The dysregulation of iNOS and/or TLR signaling pathways in the endometrium mediated by *NFKBIA* could be contributing to the subfertility observed in cows after uterine disease.

Eighteen percent of the discordant, pregnancy regulated transcripts (5 of 28) identified in cows after uterine infection were non-coding RNAs. While our understanding of non-coding RNA function in the bovine endometrium is poor, non-coding RNAs are known to regulate basal and gene specific transcription, translation and in some cases protein function. In addition, some non-coding RNAs may indeed encode proteins that have yet to be characterized. However, endometrial expression of non-coding RNAs in swine and goat have been characterized throughout the estrous cycle and during the peri-implantation period [46–48]. In the pig, expression of long non-coding RNAs at the time of embryo implantation were correlated with genes involved in MAPK signaling, including dual-specificity phosphatases (*DUSP4*), *DUSP10*, and *CD14* [47]. In the current study, DUSP1 was identified as a discordant, activated upstream regulator of pregnancy regulated genes in cows following uterine infection. It is speculated that DUSP1 may play a role in pregnancy establishment and maintenance by dysregulating MAPK signaling required for pregnancy success in the bovine [49, 50]. We did not analyze specific non-coding RNAs or their potential targets in the current study; however, the expression of non-coding RNAs and their role in inflammation and pregnancy establishment require further investigation in the cow and may lead to novel targets for intervention or identification of genes involved in pregnancy establishment.

Although endometrium from cows infused with vehicle had insufficient RNA integrity for RNAseq, qPCR was used to compare 8 selected endometrial genes between vehicle and bacteria infused animals. While some genes were not significantly affected following intrauterine infusion, *TRANK1* expression was dependent on the interaction between pregnancy and intrauterine infusion. The data describe an effect of pregnancy status on *TRANK1* expression only in cows bacteria infused cows, with lower expression of *TRANK1* in non-pregnant bacteria infused cows compared to non-pregnant bacteria infused cows. This suggests that endometrial *TRANK1* expression could play a role in uterine infection related subfertility and one could speculate that reduced *TRANK1* expression, mediated by bacterial infection, does not permit the establish of pregnancy. The function of TRANK1 has been best described in the brain for its role in neurological disorders, and both female and male homozygous *Trank1* knock-out mice are reported to be fertile [51]. The role of *TRANK1* in the bovine endometrium has not been reported; however, endometrial *TRANK1* expression is increased in early decidualized stromal cells compared to later or non-decidualized cells of women [52]. With such little information known regarding *TRANK1* and its role in the bovine endometrium, further investigation is required before a mechanistic link between fertility and uterine infection can be hypothesized.

The low integrity of RNA isolated from vehicle infused cows limited our ability to execute our ideal experiment of using RNA sequencing to analyze the effect of intrauterine infusion on the endometrial signature of pregnancy in cows. Despite this drawback, we were able to compare our transcriptome analysis after induction of uterine infection to that of previous reports using healthy cows to identify endometrial genes that may contribute to post infection subfertility. However, the previous reports utilized different breeds of cows, different days of pregnancy (d15, d16, d17), different transcriptome tools (microarray), and performed sequence alignment with older versions of the bovine genome than used in the current study; all of which could increase variation in the final analysis performed here. Alternatively, these differences in experimental strategies could be viewed as a strength, as this collective analysis using very stringent criteria identified a robust set of pregnancy associated genes that were consistently characterized amongst the four studies. However, caution should be applied when interpreting individual gene expression from the comparison of all four studies. In future studies to identify genes and mechanisms associated with infection related infertility, we should consider collecting endometrial samples at earlier stages of pregnancy. Furthermore, we must account for the potential impacts of previous uterine infection on oocyte and embryo viability. The developmental competence of oocytes after bacterial infusion was similar between pregnant and non-pregnant cows in the current study based on in vitro fertilization and embryo culture [10], but perhaps future experiments should utilize embryo transfer from healthy donors to recipients after infusion of bacteria to better separate potential effects on the germ line from those of the endometrium.

In conclusion, we characterized the endometrial transcriptomic signature of pregnancy in cows after a uterine infection. Furthermore, we compared these new data with those from previous studies using healthy cows and identified a robust set of genes, pathways, and upstream regulators that represent an essential transcriptomic signature of pregnancy. Additionally, we identified pregnancy regulated genes unique to the endometrium of cows following uterine infection that were not observed in healthy cows. These genes, pathways, and upstream regulators could contribute to reduced fertility following uterine disease.

## Supporting information

S1 Fig Principal component analysis of endometrial transcript reads acquired from pregnant and non-pregnant cows after intrauterine infusion of pathogenic bacteria. Cows were inseminated 130 days after intrauterine infusion of pathogenic bacteria and endometrium was collected 16 days later. Based on the presence of an embryo and interferon tau, cows were designated as pregnant (n = 3) or non-pregnant (n = 4). Endometrium was subjected to RNA sequencing analysis. Read counts for all transcripts were subjected to principal component analysis. Principal component (PC) 1 and principal component 2 explain 29% and 23.8% of the total variance, respectively.
(DOCX)

S2 Fig Validation of RNA sequencing using real time RT-PCR. The validity of RNA sequencing of endometrium was confirmed using real time RT-PCR. Linear regression using read counts and relative expression provides the correlation coefficient and *P*-value for the expression of (A) *CPM*, (B) *OXTR*, and (C) *STC2*.
(DOCX)

S3 Fig Unique predicted upstream regulators of differentially expressed genes identified in the pregnant endometrium only after infusion with bacteria. Ingenuity Pathway Analysis identified five unique predicted upstream regulators in the endometrium of pregnant cows after infusion of pathogenic bacteria that were not identified in pregnant healthy cows. Each web depicts a single predicted upstream regulator in the center and its associated differentially expressed genes that are upregulated (red) in pregnant cows compared to non-pregnant cows. (A) AIRE, (B) NFKBIA, (C) DUSP1, (D) PF4, and (E) ISG15. Orange color predicts activation (z-score ≥ 2) and blue predicts inhibition (z-score ≤ -2), while red icons indicate upregulated genes in the endometrium of pregnant cows after infusion with bacteria.
(DOCX)

S1 Table Characteristics of cows after intrauterine infusion of vehicle or pathogenic bacteria. (DOCX)

S2 Table Differentially expressed endometrial genes at day 15 in the healthy pregnant cow compared to the non-pregnant cow from the previous study Bauersachs et al., 2012. (DOCX)

S3 Table Differentially expressed endometrial genes at day 16 in the healthy pregnant cow compared to the non-pregnant cow from the previous study Forde et al., 2012. (DOCX)

S4 Table Differentially expressed endometrial genes at day 17 in the healthy pregnant cow compared to the non-pregnant cow from the previous study Cerri et al., 2012. (DOCX)

S5 Table Primer details use for real time RT-PCR in bovine endometrium. (DOCX)

S6 Table Summary of read mapping for endometrial samples obtained from cows after intrauterine infusion of pathogenic bacteria. (DOCX)

S7 Table Most abundantly expressed endometrial genes in the cows after intrauterine infusion of pathogenic bacteria. (DOCX)

S8 Table Differentially expressed endometrial genes in pregnant cows compared to non-pregnant cows after intrauterine infusion with pathogenic bacteria. (DOCX)

S9 Table Canonical pathways and related genes altered in the endometrium of pregnant cows compared to the non-pregnant cows after intrauterine infusion of pathogenic bacteria. (DOCX)

S10 Table Gene networks altered in the endometrium of pregnant cows compared to the non-pregnant cows after intrauterine infusion of pathogenic bacteria. (DOCX)

S11 Table Predicted upstream regulators identified in the endometrium of pregnant cows compared to the non-pregnant cows after intrauterine infusion of pathogenic bacteria. (DOCX)

S12 Table Differentially expressed endometrial genes in pregnant cows compared to non-pregnant cows after intrauterine infusion with pathogenic bacteria (Log2FC ≥ 1.5 or ≤ -1.5). (DOCX)

S13 Table Differentially expressed genes in the endometrium of pregnant cows compared to non-pregnant cows in all studies (Log2FC compared to non-pregnant). (DOCX)

S14 Table Altered canonical pathways in the endometrium of pregnant cows compared to non-pregnant cows in all studies. Infected day 16, Healthy day 15, Healthy day 16, Healthy day 17.
(DOCX)

S15 Table Predicted upstream regulators in the endometrium of pregnant cows compared to non-pregnant cows using all studies. Predicted activation state was either activated (+) or inhibited (-).
(DOCX)
