# Supplementary material for: The endometrial transcriptomic response to pregnancy is altered in cows after uterine infection
Source: PLoS One. 2022 Mar 31;17(3):e0265062. doi: 10.1371/journal.pone.0265062 (PMC8970397; doi:10.1371/journal.pone.0265062)

**Supplemental Figure S1.** **Principal component analysis of endometrial transcript reads acquired from pregnant and non-pregnant cows after intrauterine infusion of pathogenic bacteria.** Cows were inseminated 130 days after intrauterine infusion of pathogenic bacteria and endometrium was collected 16 days later. Based on the presence of an embryo and interferon tau, cows were designated as pregnant (n = 3) or non-pregnant (n = 4). Endometrium was subjected to RNA sequencing analysis. Read counts for all transcripts were subjected to principal component analysis. Principal component (PC) 1 and principal component 2 explain 29% and 23.8% of the total variance, respectively.


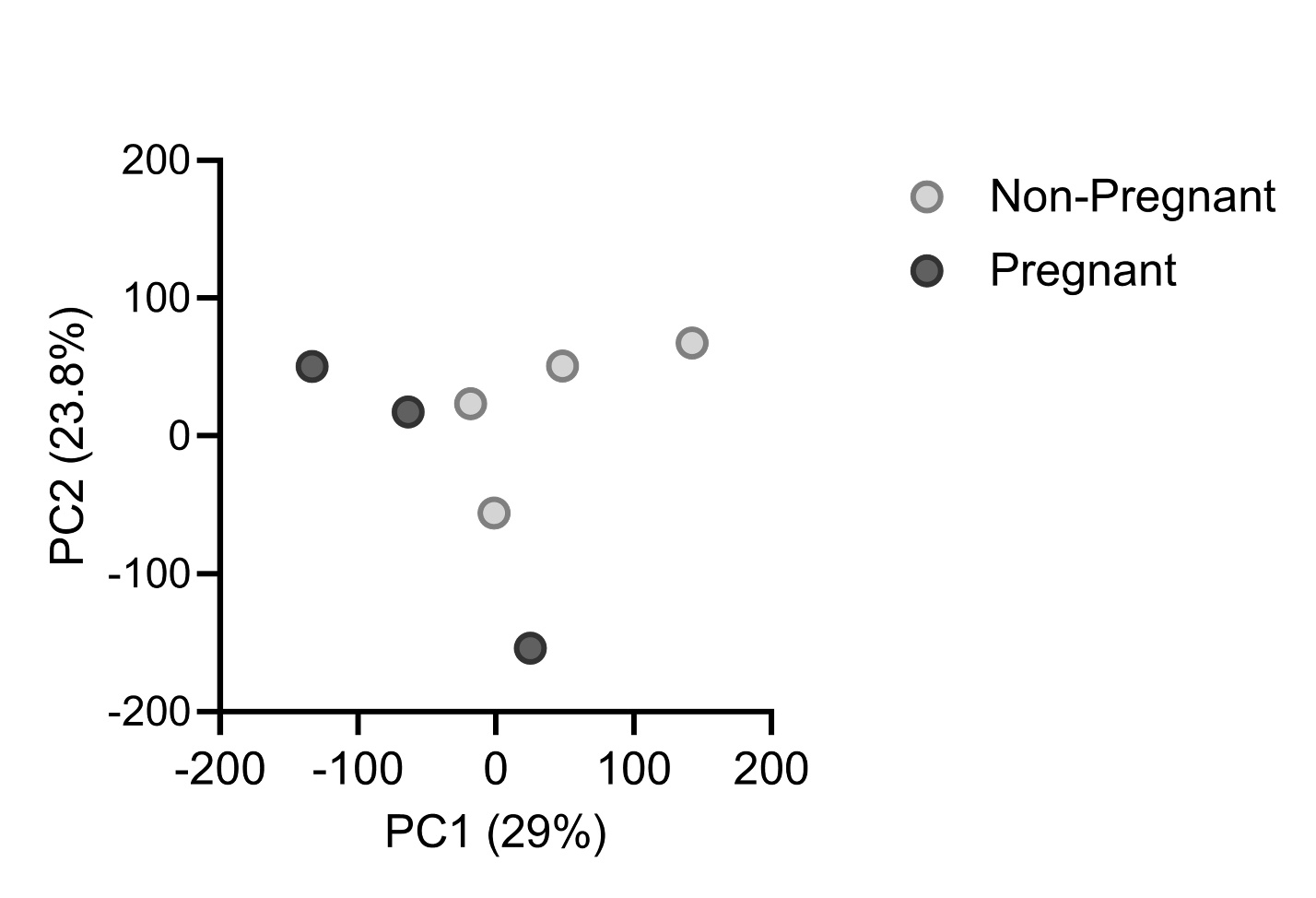

Supplement: S1 Fig — Cows were inseminated 130 days after intrauterine infusion of pathogenic bacteria and endometrium was collected 16 days later. Based on the presence of an embryo and interferon tau, cows were designated as pregnant (n = 3) or non-pregnant (n = 4). Endometrium was subjected to RNA sequencing analysis. Read counts for all transcripts were subjected to principal component analysis. Principal component (PC) 1 and principal component 2 explain 29% and 23.8% of the total variance, respectively. (DOCX) [file pone.0265062.s001.docx]
