# Supplementary material for: The endometrial transcriptomic response to pregnancy is altered in cows after uterine infection
Source: PLoS One. 2022 Mar 31;17(3):e0265062. doi: 10.1371/journal.pone.0265062 (PMC8970397; doi:10.1371/journal.pone.0265062)

**Supplemental Figure S2. Validation of RNA sequencing using real time RT-PCR.** The validity of RNA sequencing of endometrium was confirmed using real time RT-PCR. Linear regression using read counts and relative expression provides the correlation coefficient and *P*-value for the expression of (A) *CPM*, (B) *OXTR*, and (C) *STC2*


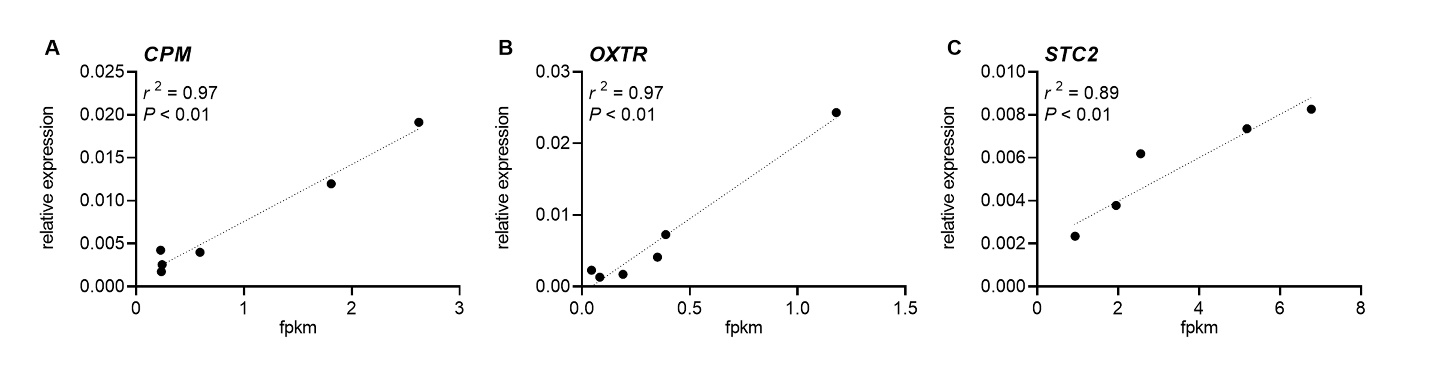

Supplement: S2 Fig — The validity of RNA sequencing of endometrium was confirmed using real time RT-PCR. Linear regression using read counts and relative expression provides the correlation coefficient and P-value for the expression of (A) CPM, (B) OXTR, and (C) STC2. (DOCX) [file pone.0265062.s002.docx]
