# Supplementary material for: The endometrial transcriptomic response to pregnancy is altered in cows after uterine infection
Source: PLoS One. 2022 Mar 31;17(3):e0265062. doi: 10.1371/journal.pone.0265062 (PMC8970397; doi:10.1371/journal.pone.0265062)

**Supplemental Figure S3. Unique predicted upstream regulators of differentially expressed genes identified in the pregnant endometrium only after infusion with bacteria**. Ingenuity Pathway Analysis identified five unique predicted upstream regulators in the endometrium of pregnant cows after infusion of pathogenic bacteria that were not identified in pregnant healthy cows. Each web depicts a single predicted upstream regulator in the center and its associated differentially expressed genes that are upregulated (red) in pregnant cows compared to non-pregnant cows. (A) AIRE, (B) NFKBIA, (C) DUSP1, (D) PF4, and (E) ISG15. Orange color predicts activation (z-score ≥ 2) and blue predicts inhibition (z-score ≤ -2), while red icons indicate upregulated genes in the endometrium of pregnant cows after infusion with bacteria.


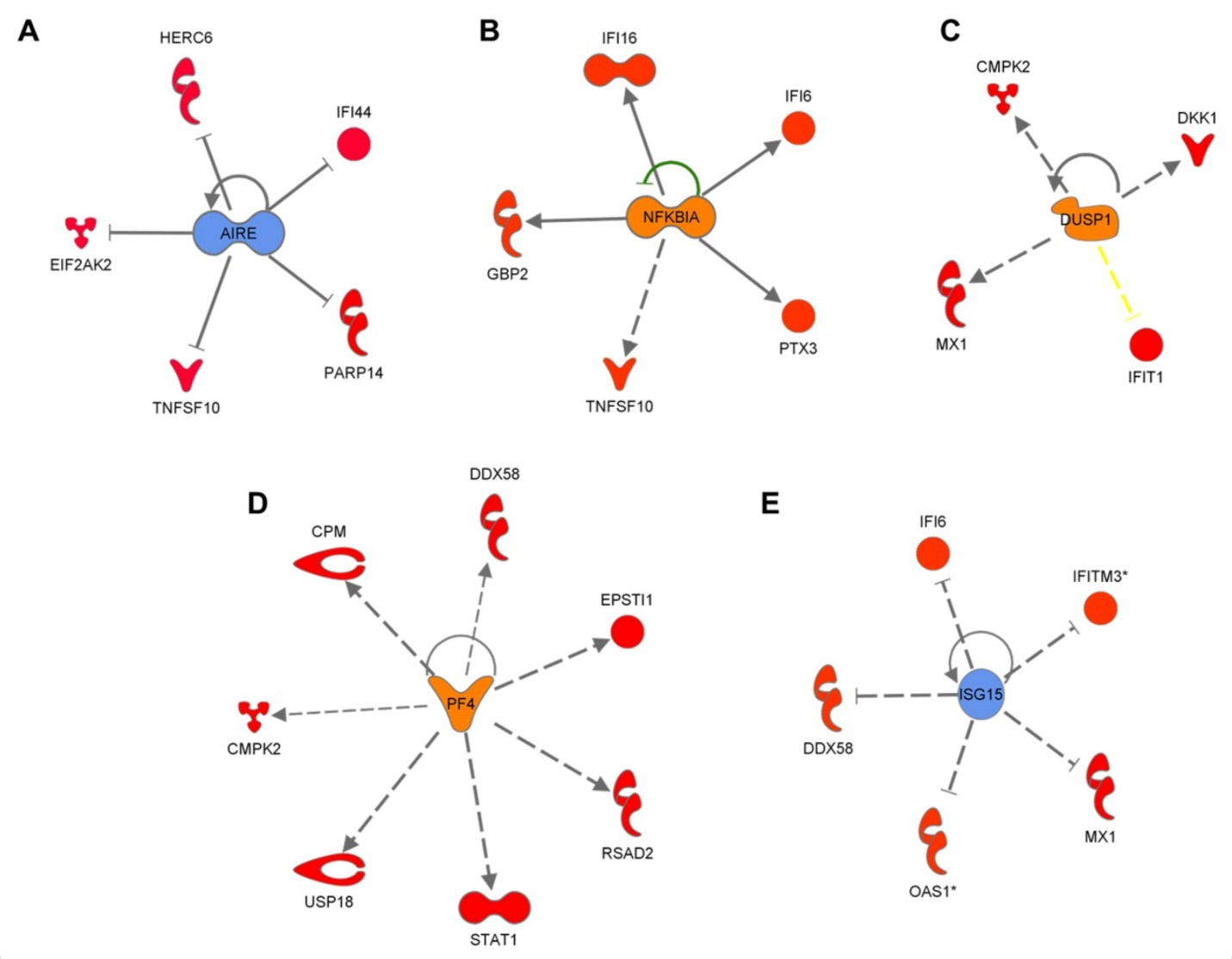

Supplement: S3 Fig — Ingenuity Pathway Analysis identified five unique predicted upstream regulators in the endometrium of pregnant cows after infusion of pathogenic bacteria that were not identified in pregnant healthy cows. Each web depicts a single predicted upstream regulator in the center and its associated differentially expressed genes that are upregulated (red) in pregnant cows compared to non-pregnant cows. (A) AIRE, (B) NFKBIA, (C) DUSP1, (D) PF4, and (E) ISG15. Orange color predicts activation (z-score ≥ 2) and blue predicts inhibition (z-score ≤ -2), while red icons indicate upregulated genes in the endometrium of pregnant cows after infusion with bacteria. (DOCX) [file pone.0265062.s003.docx]
