# Supplementary material for: The endometrial transcriptomic response to pregnancy is altered in cows after uterine infection
Source: PLoS One. 2022 Mar 31;17(3):e0265062. doi: 10.1371/journal.pone.0265062 (PMC8970397; doi:10.1371/journal.pone.0265062)
Supplement: S1 Table — (DOCX) [file pone.0265062.s004.docx]

**S1 Table. Characteristics of cows after intrauterine infusion of vehicle or pathogenic bacteria.**

|  | Vehicle | | Bacteria | | *P* value^a^ | |
| --- | --- | --- | --- | --- | --- | --- |
|  | Non-Pregnant | Pregnant | Non-Pregnant | Pregnant | Trt | Preg |
| *N* | 5 | 6 | 5 | 7 |  |  |
| CL diameter (mm)^b^ | 21.8 ± 0.7 | 23.3 ± 1.3 | 23.4 ± 0.6 | 21.8 ± 0.6 | 0.99 | 0.96 |
| P4 (ng/mL) ^c^ | 12.0 ± 0.9 | 11.8 ± 0.7 | 11.7 ± 1.4 | 11.2 ± 1.5 | 0.72 | 0.80 |
| IFNT (ng/mL) ^b^ | 0.1 ± 0.0 | 765.0 ± 384.8 | 0.0 ± 0.0 | 1685.8 ± 978.6 | 0.49 | 0.07 |
| Oocyte (n) ^d^ | 37.2 ± 10.6 | 42.0 ± 8.0 | 40.4 ± 8.1 | 41.6 ± 9.6 | 0.88 | 0.75 |
| Morula (n) ^d^ | 8.6 ± 2.2 | 11.0 ± 1.7 | 10.6 ± 2.7 | 8.3 ± 2.6 | 0.88 | 0.99 |
| Morula/oocyte (%) | 25.3 ± 2.2 | 27.9 ± 4.0 | 25.3 ± 4.8 | 16.5 ± 3.4 | 0.14 | 0.42 |

^a^ Comparison of vehicle or bacteria intrauterine infusion (Trt) or pregnancy status (Preg).

^b^ Quantified 16 days post insemination at time of conceptus collection.

^c^ Circulating progesterone quantified 15 days post insemination.

^d^ Sum of four rounds of oocytes collections, in vitro fertilization, and embryo culture.
