# Supplementary material for: The endometrial transcriptomic response to pregnancy is altered in cows after uterine infection
Source: PLoS One. 2022 Mar 31;17(3):e0265062. doi: 10.1371/journal.pone.0265062 (PMC8970397; doi:10.1371/journal.pone.0265062)
Supplement: S2 Table — (DOCX) [file pone.0265062.s005.docx]

**S2 Table. Differentially expressed endometrial genes at day 15 in the healthy pregnant cow compared to the non-pregnant cow from the previous study Bauersachs et al., 2012.**

| Gene ID | Symbol | Log_2_FC | Adj *P* Value |
| --- | --- | --- | --- |
| 510774 | *ABHD1* | 1.513 | 0.002 |
| 505134 | *ADAR* | 1.651 | 0.002 |
| 505518 | *C15H11orf34* | 3.433 | 0.014 |
| 280678 | *C4A* | 1.756 | 0.010 |
| 281044 | *CCL8* | 2.354 | 0.001 |
| 511001 | *CLEC4F* | 2.933 | 0.001 |
| 505167 | *CRYM* | 1.537 | 0.017 |
| 615107 | *CXCL10* | 2.951 | 0.001 |
| 504760 | *DDX58* | 2.303 | 0.002 |
| 508378 | *DHX58* | 2.572 | 0.001 |
| 504445 | *DKK1* | 2.049 | 0.046 |
| 515051 | *DTX3L* | 2.353 | 0.001 |
| 347700 | *EIF2AK2* | 2.981 | 0.001 |
| 281751 | *EIF4E* | 1.544 | 0.001 |
| 614555 | *EPSTI1* | 2.743 | 0.001 |
| 281758 | *FABP3* | 2.477 | 0.012 |
| 510874 | *FBXO17* | 1.885 | 0.000 |
| 613313 | *GBP4* | 2.592 | 0.035 |
| 516949 | *GBP5* | 2.421 | 0.001 |
| 527520 | *HERC6* | 1.661 | 0.010 |
| 506759 | *IFI16* | 2.479 | 0.001 |
| 507138 | *IFI27* | 3.076 | 0.002 |
| 508348 | *IFI44* | 4.155 | 0.001 |
| 508347 | *IFI44L* | 3.826 | 0.000 |
| 512913 | *IFI6* | 2.461 | 0.001 |
| 535490 | *IFIH1* | 2.44 | 0.002 |
| 515091 | *IFIT5* | 2.203 | 0.000 |
| 353510 | *IFITM1* | 1.886 | 0.021 |
| 100125591 | *IRF7* | 2.148 | 0.001 |
| 509855 | *IRF9* | 2.56 | 0.001 |
| 281871 | *ISG15* | 5.243 | 0.001 |
| 506604 | *ISG20* | 3.191 | 0.003 |
| 100139670 | *LOC100139670* | 4.786 | 0.001 |
| 504861 | *LOC504861* | 1.56 | 0.007 |
| 509283 | *LOC509283* | 2.917 | 0.001 |
| 512486 | *LOC512486* | 2.09 | 0.032 |

S2 Table. Continued

| Gene ID | Symbol | Log_2_FC | Adj *P* Value |
| --- | --- | --- | --- |
| 618737 | *LOC618737* | 4.536 | 0.001 |
| 281908 | *MFAP5* | 1.653 | 0.015 |
| 280872 | *MX1* | 3.642 | 0.004 |
| 280873 | *MX2* | 5.321 | 0.000 |
| 654488 | *OAS1Y* | 3.662 | 0.001 |
| 513185 | *PARP12* | 2.122 | 0.001 |
| 540789 | *PARP14* | 2.527 | 0.001 |
| 510532 | *PARP9* | 1.795 | 0.001 |
| 767910 | *PLAC8B* | 2.278 | 0.009 |
| 100138545 | *PML* | 1.972 | 0.001 |
| 508877 | *PNPT1* | 2.393 | 0.001 |
| 280701 | *PPA1* | 1.644 | 0.001 |
| 506415 | *RSAD2* | 4.842 | 0.001 |
| 532442 | *RTP4* | 3.342 | 0.000 |
| 514205 | *SAMD9* | 3.851 | 0.001 |
| 539759 | *SIGLEC1* | 2.268 | 0.012 |
| 521795 | *SLFN11* | 2.952 | 0.021 |
| 100140338 | *SP100* | 2.393 | 0.003 |
| 510377 | *SP140* | 1.629 | 0.002 |
| 510814 | *STAT1* | 1.69 | 0.002 |
| 784029 | *TDGF1* | -1.581 | 0.045 |
| 783855 | *TIFA* | 1.629 | 0.004 |
| 507549 | *TIMD4* | 1.61 | 0.040 |
| 507215 | *TNFSF10* | 1.772 | 0.005 |
| 523970 | *TRAK2* | 2.419 | 0.008 |
| 509859 | *TRANK1* | 1.567 | 0.001 |
| 497204 | *UBA7* | 3.268 | 0.001 |
| 515202 | *USP18* | 3.642 | 0.001 |
| 509740 | *XAF1* | 2.687 | 0.001 |
| 508333 | *ZBP1* | 2.973 | 0.001 |
| 539807 | *ZNFX1* | 2.768 | 0.000 |
