# Supplementary material for: The endometrial transcriptomic response to pregnancy is altered in cows after uterine infection
Source: PLoS One. 2022 Mar 31;17(3):e0265062. doi: 10.1371/journal.pone.0265062 (PMC8970397; doi:10.1371/journal.pone.0265062)
Supplement: S3 Table — (DOCX) [file pone.0265062.s006.docx]

**S3 Table. Differentially expressed endometrial genes at day 16 in the healthy pregnant cow compared to the non-pregnant cow from the previous study Forde et al., 2012.**

| Gene ID | Symbol | Log_2_FC | Adj *P* Value |
| --- | --- | --- | --- |
| 510774 | *ABHD1* | 2.315 | 1.17E-47 |
| 505649 | *ACCS* | -1.515 | 4.98E-04 |
| 281672 | *ACKR4* | 6.069 | 2.79E-04 |
| 532272 | *ADAMTS13* | -2.07 | 2.35E-07 |
| 505134 | *ADAR* | 2.815 | 7.62E-47 |
| 525795 | *AGRN* | 1.963 | 6.53E-16 |
| 327662 | *ANXA1* | 1.644 | 1.05E-04 |
| 506045 | *ATAD1* | 1.584 | 1.91E-05 |
| 512313 | *ATP2A3* | -1.89 | 2.25E-04 |
| 100137953 | *ATP8B4* | 4.727 | 9.13E-15 |
| 280729 | *B2M* | 1.813 | 1.38E-08 |
| 515585 | *B3GNT2* | 1.527 | 4.32E-06 |
| 522469 | *BATF2* | 3.435 | 1.70E-08 |
| 533338 | *BCL2L12* | 1.535 | 1.01E-06 |
| 280734 | *BPI* | 5.029 | 4.87E-05 |
| 280737 | *BTC* | 1.522 | 1.69E-04 |
| 326579 | *BZW2* | 2.22 | 6.45E-05 |
| 617435 | *C1QB* | 1.897 | 1.22E-06 |
| 509968 | *C1QC* | 1.683 | 4.58E-05 |
| 767827 | *C1S* | 1.551 | 3.14E-06 |
| 515440 | *C2* | 2.18 | 2.37E-09 |
| 540702 | *C3AR1* | 1.892 | 8.91E-05 |
| 280678 | *C4A* | 3.039 | 1.90E-09 |
| 515918 | *CA8* | -2.177 | 2.10E-06 |
| 338039 | *CASP4* | 2.418 | 7.26E-07 |
| 507481 | *CASP8* | 1.704 | 5.32E-13 |
| 529166 | *CBLN3* | 1.892 | 6.78E-05 |
| 404072 | *CCL11* | 3.81 | 2.20E-05 |
| 524530 | *CCND1* | -1.626 | 3.46E-05 |
| 533834 | *CD274* | 4.545 | 3.00E-04 |
| 286849 | *CD40* | 1.667 | 4.09E-04 |
| 505040 | *CD53* | 2.075 | 5.26E-09 |
| 539690 | *CD93* | -2.163 | 5.05E-08 |
| 513265 | *CDKN2AIP* | 1.627 | 5.72E-05 |
| 782472 | *CGAS* | 2.481 | 1.13E-06 |
| 515280 | *CHP2* | -1.793 | 2.71E-04 |

S3 Table. Continued.

| Gene ID | Symbol | Log_2_FC | Adj *P* Value |
| --- | --- | --- | --- |
| 508740 | *CLEC12A* | 1.92 | 1.07E-03 |
| 511001 | *CLEC4F* | 77.35 | 3.14E-26 |
| 784304 | *CMPK2* | 10.265 | 4.07E-34 |
| 509620 | *CMTR1* | 1.984 | 2.82E-36 |
| 784375 | *CNTNAP3* | -2.614 | 6.42E-04 |
| 613849 | *COL13A1* | 1.622 | 9.59E-04 |
| 338086 | *COX7A1* | 1.554 | 9.07E-08 |
| 785528 | *CWH43* | 1.982 | 3.22E-06 |
| 615107 | *CXCL10* | 4.553 | 1.55E-05 |
| 541171 | *DIPK1A* | 1.502 | 7.70E-10 |
| 504445 | *DKK1* | 2.794 | 4.54E-06 |
| 533992 | *DRAM1* | 1.854 | 1.59E-06 |
| 515051 | *DTX3L* | 4.78 | 3.23E-23 |
| 281750 | *EDNRB* | 1.753 | 7.50E-04 |
| 347700 | *EIF2AK2* | 5.511 | 1.75E-20 |
| 768233 | *ELMOD1* | 2.068 | 1.21E-04 |
| 614555 | *EPSTI1* | 9.56 | 5.29E-25 |
| 539571 | *ESM1* | -2.565 | 1.80E-04 |
| 280685 | *F2* | -1.775 | 5.35E-04 |
| 615144 | *FAM171A2* | -1.788 | 6.21E-04 |
| 514701 | *FAM3B* | 1.985 | 3.23E-04 |
| 508561 | *FAM83D* | -2.668 | 7.61E-04 |
| 282227 | *FCGR1A* | 1.987 | 4.65E-04 |
| 540142 | *FOXS1* | 3.102 | 5.98E-14 |
| 510714 | *FRMD4A* | -1.593 | 5.19E-05 |
| 526127 | *FRMD4B* | 1.603 | 1.14E-04 |
| 613313 | *GBP4* | 7.114 | 1.57E-12 |
| 525937 | *GDA* | 1.705 | 7.18E-04 |
| 508774 | *GDAP2* | 1.626 | 5.06E-05 |
| 281797 | *GNGT2* | 3.788 | 7.66E-04 |
| 517332 | *GRAMD1B* | -1.504 | 8.03E-04 |
| 510225 | *GRINA* | 1.581 | 5.74E-05 |
| 513231 | *GTF2B* | 1.836 | 1.01E-04 |
| 514373 | *HERC5* | 3.576 | 3.77E-12 |
| 527520 | *HERC6* | 10.559 | 4.73E-26 |
| 507480 | *HES4* | 2.135 | 4.19E-04 |
| 616129 | *HOXB2* | -1.517 | 6.55E-04 |
| 768240 | *HOXB4* | -1.782 | 1.86E-04 |

S3 Table. Continued.

| Gene ID | Symbol | Log_2_FC | Adj *P* Value |
| --- | --- | --- | --- |
| 100848273 | *HSH2D* | 3.996 | 4.52E-05 |
| 506281 | *IDO1* | 5.563 | 4.17E-07 |
| 506759 | *IFI16* | 5.177 | 1.72E-21 |
| 507138 | *IFI27* | 6.384 | 1.26E-28 |
| 510697 | *IFI35* | 2.018 | 1.25E-13 |
| 508347 | *IFI44L* | 18.086 | 8.82E-17 |
| 512913 | *IFI6* | 8.766 | 5.15E-22 |
| 535490 | *IFIH1* | 5.57 | 8.39E-20 |
| 527528 | *IFIT2* | 12.752 | 3.57E-12 |
| 509678 | *IFIT3* | 12.505 | 1.00E-19 |
| 515091 | *IFIT5* | 5.41 | 6.67E-27 |
| 511022 | *IL23A* | 1.955 | 8.47E-12 |
| 788637 | *IQCN* | -3.774 | 2.04E-04 |
| 516979 | *IRF3* | 1.564 | 1.54E-16 |
| 509855 | *IRF9* | 4.868 | 1.71E-70 |
| 281871 | *ISG15* | 54.688 | 5.28E-69 |
| 506604 | *ISG20* | 13.44 | 1.71E-13 |
| 509394 | *ISYNA1* | -1.58 | 4.29E-04 |
| 506526 | *ITGA10* | 1.65 | 2.44E-05 |
| 521931 | *KCNK12* | -3.757 | 4.42E-04 |
| 514211 | *KIAA1755* | 1.562 | 5.01E-13 |
| 281889 | *KRT17* | 4.883 | 7.07E-05 |
| 520327 | *KYNU* | 5.706 | 3.16E-04 |
| 531327 | *LAMP3* | 2.308 | 2.84E-05 |
| 531137 | *LGALS3BP* | 2.102 | 2.76E-12 |
| 510813 | *LGALS9* | 2.579 | 1.33E-15 |
| 100125267 | *LIPA* | 1.514 | 5.90E-04 |
| 100139670 | *LOC100139670* | 42.135 | 2.46E-31 |
| 112441507 | *LOC112441507* | 17.808 | 1.75E-44 |
| 112442826 | *LOC112442826* | 2.743 | 3.94E-04 |
| 509283 | *LOC509283* | 5.979 | 1.27E-21 |
| 511531 | *LOC511531* | 4.251 | 1.32E-14 |
| 512672 | *LOC512672* | 2.553 | 3.22E-06 |
| 513659 | *LOC513659* | 2.17 | 2.73E-04 |
| 616948 | *LOC616948* | 2.435 | 3.31E-07 |
| 506141 | *LOC618409* | 3.188 | 1.18E-15 |
| 781710 | *LOC781710* | 3.637 | 6.45E-09 |
| 516507 | *LRRC66* | 2.141 | 1.01E-03 |

S3 Table. Continued.

| Gene ID | Symbol | Log_2_FC | Adj *P* Value |
| --- | --- | --- | --- |
| 510977 | *LY6E* | 1.804 | 2.75E-05 |
| 535622 | *MAP3K8* | 1.873 | 1.47E-07 |
| 281296 | *MAPT* | -1.557 | 1.02E-03 |
| 505267 | *MFNG* | -1.521 | 8.86E-04 |
| 510644 | *MICALL2* | -1.514 | 4.44E-05 |
| 790225 | *MLKL* | 2.102 | 8.83E-05 |
| 513807 | *MORC3* | 1.757 | 2.42E-06 |
| 523206 | *MOV10* | 1.555 | 7.37E-16 |
| 525504 | *MST1R* | 1.589 | 2.24E-10 |
| 280872 | *MX1* | 14.694 | 2.87E-29 |
| 280873 | *MX2* | 52.595 | 4.86E-26 |
| 520472 | *NAMPT* | 1.645 | 8.20E-04 |
| 614457 | *NRIP2* | -1.621 | 8.55E-05 |
| 525562 | *NRTN* | -1.796 | 1.09E-04 |
| 347699 | *OAS1X* | 23.646 | 1.66E-10 |
| 654488 | *OAS1Y* | 12.478 | 4.14E-48 |
| 519922 | *OAS1Z* | 13.977 | 5.34E-30 |
| 529660 | *OAS2* | 16.557 | 3.11E-43 |
| 534150 | *OPTN* | 1.592 | 3.28E-05 |
| 281371 | *OXTR* | -7.406 | 4.49E-04 |
| 767936 | *P2RY14* | -1.541 | 6.76E-04 |
| 518368 | *PARM1* | 1.658 | 3.62E-06 |
| 510991 | *PARP10* | 2.749 | 1.01E-29 |
| 513185 | *PARP12* | 3.201 | 4.58E-23 |
| 540789 | *PARP14* | 6.324 | 1.22E-22 |
| 510532 | *PARP9* | 3.647 | 1.62E-23 |
| 515067 | *PGAM2* | -1.712 | 1.78E-05 |
| 767910 | *PLAC8B* | 6.858 | 4.74E-27 |
| 281983 | *PLAUR* | 1.52 | 8.67E-04 |
| 510408 | *PLCL2* | -1.518 | 5.09E-04 |
| 524990 | *PLVAP* | -1.74 | 4.82E-04 |
| 617469 | *PMEPA1* | -1.501 | 1.03E-03 |
| 100138545 | *PML* | 2.869 | 3.91E-32 |
| 508877 | *PNPT1* | 3.411 | 3.58E-13 |
| 541218 | *POLK* | 1.569 | 9.03E-06 |
| 280701 | *PPA1* | 2.448 | 6.65E-12 |
| 282091 | *PPP1R16B* | -1.603 | 8.89E-04 |
| 100137803 | *PRDM16* | -2.647 | 1.02E-03 |

S3 Table. Continued.

| Gene ID | Symbol | Log_2_FC | Adj *P* Value |
| --- | --- | --- | --- |
| 539141 | *PSMA2* | 1.724 | 2.84E-11 |
| 509857 | *PSME2* | 1.498 | 1.15E-07 |
| 617807 | *PSMF1* | 2.138 | 1.04E-14 |
| 505285 | *PTPRE* | 2.281 | 2.71E-05 |
| 513223 | *RASEF* | 1.739 | 3.16E-06 |
| 617625 | *RBM43* | 2.384 | 5.06E-06 |
| 507427 | *RIPK3* | 1.629 | 7.72E-04 |
| 282341 | *RNASE6* | 1.852 | 2.55E-07 |
| 100048947 | *RNASEL* | 1.593 | 1.68E-07 |
| 513479 | *RNF114* | 1.596 | 1.60E-57 |
| 767991 | *RNF24* | -1.508 | 3.73E-04 |
| 506415 | *RSAD2* | 36.622 | 2.85E-47 |
| 511675 | *RSPO1* | 7.054 | 2.48E-06 |
| 532442 | *RTP4* | 5.497 | 1.13E-34 |
| 504467 | *SASS6* | 2.012 | 9.44E-04 |
| 790815 | *SCLY* | 1.592 | 4.30E-10 |
| 539321 | *SERTAD1* | 1.584 | 1.25E-04 |
| 506155 | *SGCG* | -4.031 | 4.87E-07 |
| 539087 | *SHFL* | 1.592 | 2.42E-06 |
| 616861 | *SHISA5* | 1.955 | 1.05E-11 |
| 539759 | *SIGLEC1* | 4.862 | 1.06E-34 |
| 513984 | *SLC15A3* | 2.407 | 4.15E-18 |
| 282484 | *SLC34A2* | -1.677 | 9.58E-04 |
| 527023 | *SLC40A1* | 1.985 | 7.20E-06 |
| 524085 | *SLC41A3* | -1.511 | 1.36E-04 |
| 506958 | *SLC66A3* | 1.663 | 1.42E-04 |
| 514339 | *SLC6A12* | 15.228 | 7.84E-04 |
| 508174 | *SLC7A9* | 6.147 | 5.56E-05 |
| 521795 | *SLFN11* | 9.479 | 3.60E-12 |
| 504287 | *SOAT1* | 2.244 | 3.07E-04 |
| 515204 | *SP110* | 3.186 | 2.60E-14 |
| 100139208 | *SP140L* | 3.753 | 4.20E-23 |
| 510814 | *STAT1* | 3.722 | 2.70E-33 |
| 511023 | *STAT2* | 1.925 | 1.51E-11 |
| 540850 | *SYT7* | -1.58 | 1.55E-07 |
| 524959 | *TAP1* | 1.808 | 1.07E-11 |
| 617047 | *TCIM* | 1.609 | 1.46E-05 |
| 506702 | *TDRD7* | 2.536 | 4.94E-15 |

S3 Table. Continued.

| Gene ID | Symbol | Log_2_FC | Adj *P* Value |
| --- | --- | --- | --- |
| 533862 | *TENT2* | 1.587 | 1.22E-04 |
| 783855 | *TIFA* | 3.301 | 1.83E-11 |
| 507549 | *TIMD4* | 3.009 | 6.65E-06 |
| 540975 | *TIPARP* | 1.552 | 5.00E-05 |
| 533038 | *TM4SF1* | 1.66 | 1.06E-07 |
| 508269 | *TMEM106A* | 1.638 | 4.01E-07 |
| 515475 | *TMEM140* | 1.756 | 3.05E-06 |
| 618298 | *TMEM182* | 2.251 | 8.00E-04 |
| 507215 | *TNFSF10* | 2.95 | 6.11E-07 |
| 504507 | *TNFSF13B* | 5.113 | 3.82E-07 |
| 505683 | *TPGS1* | -1.641 | 3.81E-04 |
| 509859 | *TRANK1* | 2.573 | 2.36E-29 |
| 506467 | *TREM2* | -2.225 | 4.52E-04 |
| 282099 | *TREX1* | 2 | 1.12E-06 |
| 359715 | *TRIM21* | 1.547 | 5.74E-04 |
| 510923 | *TRIM25* | 1.656 | 4.92E-12 |
| 539820 | *TRIM34* | 2.64 | 3.09E-10 |
| 514896 | *TRIM56* | 1.595 | 8.36E-05 |
| 497204 | *UBA7* | 8.616 | 1.82E-65 |
| 509471 | *UBE2L6* | 2.113 | 6.09E-11 |
| 504557 | *UPB1* | 2.913 | 8.33E-04 |
| 515202 | *USP18* | 14.555 | 2.50E-33 |
| 524531 | *USP25* | 1.596 | 2.25E-06 |
| 509740 | *XAF1* | 6.623 | 7.87E-25 |
| 517417 | *XRN2* | 1.502 | 1.06E-08 |
| 508333 | *ZBP1* | 17.9 | 2.88E-32 |
| 509706 | *ZNF74* | -1.5 | 5.99E-08 |
| 539807 | *ZNFX1* | 5.262 | 1.05E-81 |
