# Supplementary material for: The endometrial transcriptomic response to pregnancy is altered in cows after uterine infection
Source: PLoS One. 2022 Mar 31;17(3):e0265062. doi: 10.1371/journal.pone.0265062 (PMC8970397; doi:10.1371/journal.pone.0265062)
Supplement: S4 Table — (DOCX) [file pone.0265062.s007.docx]

**S4 Table. Differentially expressed endometrial genes at day 17 in the healthy pregnant cow compared to the non-pregnant cow from the previous study Cerri et al., 2012.**

| Gene ID | Symbol | Log_2_FC | Adj *P* Value |
| --- | --- | --- | --- |
| 511515 | *AATK* | 1.686 | 1.63E-06 |
| 510774 | *ABHD1* | 3.209 | 3.52E-10 |
| 613794 | *ABLIM3* | 1.552 | 3.51E-07 |
| 505134 | *ADAR* | 2.083 | 6.89E-17 |
| 534286 | *ALAS1* | 1.64 | 1.01E-10 |
| 407169 | *ALOX12* | 3.504 | 8.58E-12 |
| 613869 | *ALOX5AP* | 2.649 | 2.73E-10 |
| 518393 | *AOX4* | 1.586 | 3.48E-04 |
| 518752 | *ARG2* | 2.007 | 3.11E-07 |
| 616246 | *ARHGAP15* | 2.559 | 9.90E-08 |
| 506075 | *ARHGEF25* | -1.531 | 9.61E-07 |
| 506045 | *ATAD1* | 1.644 | 1.54E-07 |
| 515266 | *ATF3* | 2.402 | 8.93E-10 |
| 790880 | *ATXN3* | 2.187 | 1.47E-12 |
| 533338 | *BCL2L12* | 2.421 | 9.62E-13 |
| 508365 | *BCL2L14* | 1.653 | 7.97E-07 |
| 509786 | *BCL2L15* | 2.537 | 7.98E-11 |
| 282534 | *BOLA-DQA1* | 2.002 | 8.91E-03 |
| 282535 | *BOLA-DQA2* | 3.793 | 8.12E-03 |
| 326579 | *BZW2* | 1.613 | 3.35E-08 |
| 505518 | *C15H11orf34* | 8.707 | 3.74E-15 |
| 617435 | *C1QB* | 1.896 | 6.66E-12 |
| 509968 | *C1QC* | 1.982 | 2.21E-10 |
| 515440 | *C2* | 3.786 | 4.73E-11 |
| 529849 | *C2CD4B* | 2.283 | 1.54E-07 |
| 515918 | *CA8* | -1.725 | 2.33E-07 |
| 616136 | *CALHM6* | 2.725 | 2.73E-11 |
| 338039 | *CASP4* | 2.179 | 9.77E-14 |
| 615922 | *CCDC136* | 1.701 | 1.30E-09 |
| 281044 | *CCL8* | 4.81 | 3.38E-12 |
| 510668 | *CCR7* | 2.596 | 1.73E-06 |
| 286849 | *CD40* | 1.621 | 4.73E-07 |
| 782186 | *CD58* | 1.662 | 2.25E-06 |
| 281058 | *CD69* | 1.827 | 1.33E-09 |
| 414345 | *CD86* | 2.163 | 9.26E-09 |
| 539690 | *CD93* | -2.384 | 5.44E-09 |

S4 Table. Continued.

| Gene ID | Symbol | Log_2_FC | Adj *P* Value |
| --- | --- | --- | --- |
| 514076 | *CFB* | 1.556 | 7.71E-06 |
| 511001 | *CLEC4F* | 8.66 | 5.65E-23 |
| 618591 | *CLECL1* | 1.835 | 3.11E-06 |
| 536537 | *CNOT9* | 1.545 | 1.65E-08 |
| 280752 | *CNP* | 1.822 | 3.36E-06 |
| 781493 | *COL14A1* | -2.28 | 3.59E-08 |
| 534505 | *CPXM2* | 1.943 | 6.13E-04 |
| 540605 | *CREM* | 2.251 | 1.27E-10 |
| 281719 | *CRYAB* | 2.267 | 4.46E-12 |
| 281724 | *CRYGS* | 3.888 | 2.07E-08 |
| 505167 | *CRYM* | 1.684 | 3.98E-05 |
| 615107 | *CXCL10* | 3.53 | 3.05E-10 |
| 539047 | *CYP26A1* | 2.079 | 2.53E-03 |
| 280762 | *DDC* | 2.264 | 1.55E-06 |
| 504760 | *DDX58* | 3.796 | 2.27E-14 |
| 508378 | *DHX58* | 3.422 | 4.61E-14 |
| 504445 | *DKK1* | 1.982 | 6.43E-08 |
| 512512 | *DNASE1L3* | 2.473 | 1.98E-07 |
| 533992 | *DRAM1* | 2.036 | 7.60E-13 |
| 513753 | *EDN3* | -1.819 | 7.11E-05 |
| 505206 | *EHD4* | 1.641 | 5.58E-10 |
| 347700 | *EIF2AK2* | 2.971 | 1.44E-09 |
| 282711 | *EPAS1* | 1.897 | 1.32E-06 |
| 617442 | *EVI2B* | 1.941 | 1.57E-07 |
| 281758 | *FABP3* | 4.78 | 3.16E-11 |
| 514701 | *FAM3B* | 1.735 | 1.29E-07 |
| 508882 | *FAP* | -1.621 | 2.21E-09 |
| 513483 | *FBP1* | 3.96 | 5.64E-06 |
| 282227 | *FCGR1A* | 2.413 | 9.36E-14 |
| 281812 | *FGFBP1* | 4.156 | 7.52E-08 |
| 540142 | *FOXS1* | 2.668 | 5.05E-17 |
| 505622 | *GALNT17* | 1.612 | 1.65E-05 |
| 613313 | *GBP4* | 2.69 | 1.32E-08 |
| 516949 | *GBP5* | 4.104 | 1.92E-09 |
| 508774 | *GDAP2* | 1.534 | 1.42E-12 |
| 523294 | *GLT8D2* | -1.868 | 4.07E-08 |
| 512826 | *GPIHBP1* | -1.611 | 3.00E-07 |
| 287025 | *GPLD1* | 2.886 | 4.18E-06 |

S4 Table. Continued.

| Gene ID | Symbol | Log_2_FC | Adj *P* Value |
| --- | --- | --- | --- |
| 533760 | *GRIK1* | -2.097 | 4.16E-05 |
| 525059 | *GSS* | 1.884 | 4.69E-06 |
| 513971 | *H1-2* | 1.633 | 1.82E-06 |
| 100126192 | *H19* | -1.687 | 2.75E-03 |
| 785042 | *HOOK1* | 3.299 | 2.68E-13 |
| 506281 | *IDO1* | 4.302 | 1.80E-12 |
| 506759 | *IFI16* | 2.845 | 4.31E-17 |
| 507138 | *IFI27* | 2.549 | 1.50E-10 |
| 510697 | *IFI35* | 1.859 | 2.86E-15 |
| 508348 | *IFI44* | 4.349 | 1.49E-12 |
| 508347 | *IFI44L* | 5.09 | 5.60E-14 |
| 512913 | *IFI6* | 2.789 | 8.22E-15 |
| 535490 | *IFIH1* | 2.714 | 6.85E-13 |
| 527528 | *IFIT2* | 6.395 | 9.72E-13 |
| 515091 | *IFIT5* | 2.249 | 1.78E-14 |
| 353510 | *IFITM1* | 1.755 | 2.56E-12 |
| 526461 | *IFITM5* | 3.728 | 3.90E-13 |
| 537487 | *IGSF10* | -2.15 | 3.26E-08 |
| 100125591 | *IRF7* | 3.95 | 6.74E-14 |
| 509855 | *IRF9* | 2.728 | 5.30E-14 |
| 617420 | *ISG12(B)* | 2.224 | 6.28E-06 |
| 281871 | *ISG15* | 5.068 | 2.32E-11 |
| 506604 | *ISG20* | 7.139 | 2.35E-15 |
| 515018 | *ISLR* | -1.94 | 1.16E-06 |
| 281889 | *KRT17* | 3.494 | 3.50E-09 |
| 531137 | *LGALS3BP* | 1.752 | 6.28E-08 |
| 510813 | *LGALS9* | 1.988 | 1.09E-11 |
| 507402 | *LOC100298356* | 5.486 | 2.79E-11 |
| 508666 | *LOC508666* | -1.563 | 5.87E-07 |
| 509283 | *LOC509283* | 3.477 | 1.64E-11 |
| 510382 | *LOC510382* | 2.754 | 9.39E-04 |
| 511531 | *LOC511531* | 2.159 | 1.21E-06 |
| 512486 | *LOC512486* | 2.922 | 4.01E-11 |
| 512672 | *LOC512672* | 1.837 | 1.12E-09 |
| 514978 | *LOC514978* | 4.788 | 1.80E-20 |
| 515676 | *LOC515676* | 3.787 | 2.72E-06 |
| 616948 | *LOC616948* | 2.236 | 1.77E-13 |
| 617696 | *LOC617696* | 1.965 | 9.10E-12 |

S4 Table. Continued.

| Gene ID | Symbol | Log_2_FC | Adj *P* Value |
| --- | --- | --- | --- |
| 539366 | *LRFN5* | -1.892 | 3.57E-04 |
| 510977 | *LY6E* | 1.628 | 1.02E-12 |
| 505805 | *LY6G6C* | 1.93 | 7.22E-07 |
| 281287 | *LYZ1* | 1.603 | 2.31E-03 |
| 527595 | *MCM10* | 2.503 | 1.08E-04 |
| 540701 | *MEP1B* | 2.207 | 1.94E-07 |
| 282276 | *MGAT4A* | 3.417 | 4.77E-11 |
| 790225 | *MLKL* | 3.941 | 8.44E-12 |
| 539387 | *MPZL1* | 1.602 | 1.01E-07 |
| 525504 | *MST1R* | 2.443 | 6.85E-12 |
| 281187 | *MSTN* | -1.608 | 7.89E-06 |
| 280872 | *MX1* | 3.177 | 1.77E-11 |
| 280873 | *MX2* | 7.814 | 5.45E-13 |
| 522392 | *MXRA8* | -1.623 | 3.21E-10 |
| 505994 | *NCDN* | 1.77 | 1.26E-05 |
| 281346 | *NCF2* | 1.594 | 2.57E-08 |
| 510003 | *NEDD4L* | 1.558 | 4.67E-12 |
| 782441 | *NLRC5* | 2.451 | 6.05E-10 |
| 511280 | *NMI* | 1.717 | 1.71E-13 |
| 511858 | *NT5C3A* | 1.708 | 3.38E-10 |
| 347699 | *OAS1* | 3.883 | 3.03E-13 |
| 514720 | *OSMR* | 2.064 | 1.21E-05 |
| 281371 | *OXTR* | -2.334 | 2.70E-04 |
| 513185 | *PARP12* | 2.386 | 4.02E-15 |
| 540789 | *PARP14* | 3.131 | 4.02E-15 |
| 510532 | *PARP9* | 2.256 | 1.35E-16 |
| 537453 | *PATL1* | 2.953 | 4.43E-11 |
| 282856 | *PCK2* | 1.624 | 1.49E-08 |
| 514168 | *PDXK* | 2.621 | 2.22E-07 |
| 281401 | *PIGR* | -2.286 | 4.75E-07 |
| 509228 | *PLAC8A* | 3.805 | 3.36E-09 |
| 767910 | *PLAC8B* | 3.092 | 5.55E-14 |
| 510748 | *PLEKHA4* | 1.996 | 2.12E-09 |
| 524990 | *PLVAP* | -1.988 | 3.84E-07 |
| 513533 | *PMVK* | 1.952 | 2.74E-08 |
| 508877 | *PNPT1* | 2.947 | 2.12E-19 |
| 280701 | *PPA1* | 2.384 | 1.10E-17 |
| 282091 | *PPP1R16B* | -1.637 | 1.50E-09 |

S4 Table. Continued.

| Gene ID | Symbol | Log_2_FC | Adj *P* Value |
| --- | --- | --- | --- |
| 282000 | *PRELP* | -1.699 | 2.12E-08 |
| 510394 | *PRSS22* | 1.658 | 4.65E-05 |
| 617807 | *PSMF1* | 1.771 | 4.07E-17 |
| 541148 | *PTX3* | 3.056 | 1.44E-09 |
| 515860 | *PXDN* | 5.464 | 9.44E-14 |
| 282846 | *PYCARD* | 1.84 | 1.20E-08 |
| 282035 | *RGS16* | 2.089 | 2.72E-10 |
| 282341 | *RNASE6* | 1.761 | 3.23E-12 |
| 506415 | *RSAD2* | 4.996 | 1.59E-10 |
| 532442 | *RTP4* | 3.83 | 2.59E-11 |
| 282467 | *S100A12* | 2.739 | 1.63E-08 |
| 514205 | *SAMD9* | 4.594 | 5.36E-14 |
| 504467 | *SASS6* | 1.904 | 4.92E-13 |
| 282348 | *SCNN1A* | -1.977 | 1.17E-06 |
| 513593 | *SERINC2* | 1.673 | 4.72E-10 |
| 286871 | *SERPINA14* | 3.337 | 7.19E-07 |
| 539321 | *SERTAD1* | 1.589 | 1.01E-11 |
| 617797 | *SH3BGR* | -2.078 | 1.92E-08 |
| 617336 | *SHISA2* | 2.98 | 7.54E-06 |
| 781091 | *SHISA3* | 3.377 | 2.62E-08 |
| 616861 | *SHISA5* | 2.145 | 1.57E-15 |
| 539759 | *SIGLEC1* | 4.911 | 4.56E-20 |
| 286845 | *SLC12A2* | 1.629 | 8.53E-07 |
| 521181 | *SLC15A1* | 2.304 | 1.83E-05 |
| 505775 | *SLC16A1* | 2.604 | 3.55E-09 |
| 535872 | *SLC16A2* | -1.661 | 5.02E-09 |
| 512495 | *SLC38A5* | 1.951 | 5.52E-08 |
| 282361 | *SLC5A1* | 1.545 | 3.64E-05 |
| 518699 | *SMPDL3B* | 1.502 | 8.05E-07 |
| 518795 | *SOCS1* | 1.858 | 4.33E-09 |
| 519439 | *SOX18* | -1.595 | 5.32E-07 |
| 510377 | *SP140* | 1.921 | 5.19E-13 |
| 533103 | *SPHK2* | -1.641 | 2.38E-13 |
| 537972 | *SPTLC2* | 1.536 | 1.32E-10 |
| 100125878 | *SSLP1* | -1.548 | 8.19E-08 |
| 512369 | *STARD5* | 1.746 | 4.70E-08 |
| 510814 | *STAT1* | 1.508 | 5.29E-14 |

S4 Table. Continued.

| Gene ID | Symbol | Log_2_FC | Adj *P* Value |
| --- | --- | --- | --- |
| 511023 | *STAT2* | 1.924 | 1.05E-15 |
| 534995 | *SYNGR1* | 1.658 | 6.92E-05 |
| 404136 | *TACR3* | 1.504 | 1.49E-04 |
| 539853 | *TACSTD2* | 1.835 | 4.61E-09 |
| 524959 | *TAP1* | 1.739 | 2.11E-12 |
| 506702 | *TDRD7* | 2.157 | 4.56E-16 |
| 783855 | *TIFA* | 2.962 | 1.20E-10 |
| 507549 | *TIMD4* | 4.137 | 9.89E-13 |
| 404076 | *TKDP1* | 3.104 | 9.18E-03 |
| 515475 | *TMEM140* | 2.169 | 3.88E-12 |
| 533681 | *TMEM156* | 1.531 | 2.19E-06 |
| 505490 | *TMEM40* | 3.46 | 6.29E-06 |
| 510305 | *TMEM45B* | 1.882 | 1.22E-07 |
| 617948 | *TNFRSF13B* | 2.01 | 2.21E-09 |
| 504507 | *TNFSF13B* | 3.035 | 3.04E-11 |
| 509859 | *TRANK1* | 2.351 | 1.22E-13 |
| 282099 | *TREX1* | 3.769 | 1.65E-13 |
| 539820 | *TRIM34* | 2.01 | 6.03E-14 |
| 539001 | *TSPAN2* | -1.562 | 2.76E-05 |
| 497204 | *UBA7* | 3.803 | 1.41E-13 |
| 509471 | *UBE2L6* | 1.861 | 2.03E-11 |
| 535385 | *UNC45B* | 2.387 | 4.35E-09 |
| 613535 | *UNC93A* | 2.248 | 2.30E-07 |
| 282113 | *UPK1B* | 2.005 | 8.40E-03 |
| 515202 | *USP18* | 4.327 | 1.64E-14 |
| 613549 | *VGLL1* | 1.918 | 7.56E-05 |
| 524159 | *VWA8* | 3.179 | 1.58E-14 |
| 281576 | *WARS* | 1.6 | 7.27E-11 |
| 509740 | *XAF1* | 3.249 | 7.70E-16 |
| 539807 | *ZNFX1* | 3.006 | 1.21E-15 |
