# Supplementary material for: The endometrial transcriptomic response to pregnancy is altered in cows after uterine infection
Source: PLoS One. 2022 Mar 31;17(3):e0265062. doi: 10.1371/journal.pone.0265062 (PMC8970397; doi:10.1371/journal.pone.0265062)
Supplement: S5 Table — (DOCX) [file pone.0265062.s008.docx]

**S5 Table. Primer details use for real time RT-PCR in bovine endometrium.**

| Gene Symbol | Primer Sequence | Primer Concentration | Accession number |
| --- | --- | --- | --- |
| *ABHD1* | 5ˊ CTTTGTCCTACAGGCCCGC | 500 nM | NM_001035094.2 |
|  | 3ˊ CTTGGACTGGCTGCTTGGTA |  |  |
| *ACTB* | 5ˊ CAGAAGCACTCGTACGTGGG | 500 nM | NM_173979.3 |
|  | 3ˊ TTGGCCTTAGGGTTCAGGG |  |  |
| *CPM* | 5ˊ GGACTTCAGCTACCACCACC | 300 nM | XM_003586067.4 |
|  | 3ˊ CACGACGAGAACCCACAGG |  |  |
| *CXCL8* | 5ˊ GCAGGTATTTGTGAAGAGAGCTG | 500 nM | NM_173925.2 |
|  | 3ˊ CACAGAACATGAGGCACTGAA |  |  |
| *FAM135B* | 5ˊ CATCACACCTTGCGGGTCCGA | 300 nM | NM_001078091.2 |
|  | 3ˊ GGGACAGCTGGCTGTGGGTC |  |  |
| *FLRT1* | 5ˊ CCGGGTCTCCATCTGTGAGT | 500 nM | XM_005227213.4 |
|  | 3ˊ TAGCACATTGCGGTCTCAGG |  |  |
| *GAPDH* | 5ˊ AGGTCGGAGTGAACGGATTC | 500 nM | NM_001034034.2 |
|  | 3ˊ ATGGCGACGATGTCCACTTT |  |  |
| *IL6* | 5ˊ ATGACTTCTGCTTTCCCTACCC | 500 nM | NM_173923.2 |
|  | 3ˊ GCTGCTTTCACACTCATCATTC |  |  |

S5 Table. Continued.

| Gene Symbol | Primer Sequence | Primer Concentration | Accession number |
| --- | --- | --- | --- |
| *ISG15* | 5ˊ AGAGAGCCTGGCACCAGAAC | 500 nM | NM_174366.1 |
|  | 3ˊ TTCTGGGCGATGAACTGCTT |  |  |
| *MEF2B* | 5ˊ GTCCAGGTGGAGCAGACAAA | 500 nM | NM_001145793.1 |
|  | 3ˊ ATCAGCCCGAACTTTCGCTT |  |  |
| *MX1* | 5ˊ AGACGAGTGGAAAGGCAAAGTC | 500 nM | NM_173940.2 |
|  | 3ˊ GATGGCAATCTGGGCTTCAC |  |  |
| *OXTR* | 5ˊ AAGATCCGCACGGTCAAGAT | 500 nM | NM_174134.2 |
|  | 3ˊ TGAAAGGTGAGGCTTCCTTG |  |  |
| *RPL19* | 5ˊ ATGCCAACTCCCGCCAGCAGAT | 500 nM | NM_001040516.2 |
|  | 3ˊ TGTTTTTCCGGCATCGAGCCCG |  |  |
| *STC2* | 5ˊ CACTGTTTGGTCAACGCTGG | 500 nM | NM_001192745.3 |
|  | 3ˊ TGATGAACGACTTGCCCTGG |  |  |
| *TIMD4* | 5ˊ GGCCTCTGATTCTCTGGCTG | 500 nM | NM_001075320.1 |
|  | 3ˊ AGTCACTGGCTGACCCAAAA |  |  |
| *TRANK1* | 5ˊ CGAGCACCCAGATGGACC | 500 nM | XM_024983138.1 |
|  | 3ˊ CCACTGGTACAACTGCAGGA |  |  |
