# Supplementary material for: The endometrial transcriptomic response to pregnancy is altered in cows after uterine infection
Source: PLoS One. 2022 Mar 31;17(3):e0265062. doi: 10.1371/journal.pone.0265062 (PMC8970397; doi:10.1371/journal.pone.0265062)
Supplement: S6 Table — (DOCX) [file pone.0265062.s009.docx]

**S6 Table. Summary of read mapping for endometrial samples obtained from cows after intrauterine infusion of pathogenic bacteria.**

| Cow ID | Pregnancy Status | RNA integrity number | Raw reads | Clean reads | Mapped reads | Mapped reads (%) | Mapped transcripts |
| --- | --- | --- | --- | --- | --- | --- | --- |
| 7264 | Non-Pregnant | 7.3 | 75,154,822 | 72,581,934 | 68,360,501 | 94.18% | 22,535 |
| 7389 | Non-Pregnant | 6.9 | 60,737,768 | 58,684,410 | 56,325,262 | 95.98% | 22,238 |
| 7643 | Non-Pregnant | 8.6 | 64,057,526 | 62,250,658 | 59,647,818 | 95.82% | 22,481 |
| 8040 | Non-Pregnant | 6.8 | 67,077,856 | 65,103,720 | 62,226,869 | 95.58% | 21,566 |
| 7703 | Pregnant | 7.6 | 65,462,796 | 63,716,022 | 61,279,190 | 96.18% | 22,206 |
| 7895 | Pregnant | 8.3 | 63,594,128 | 61,764,160 | 59,331,566 | 96.06% | 22,727 |
| 8283 | Pregnant | 6.9 | 63,075,410 | 61,662,042 | 59,330,732 | 96.22% | 21,199 |
|  |  |  |  |  |  |  |  |
