# Supplementary material for: The endometrial transcriptomic response to pregnancy is altered in cows after uterine infection
Source: PLoS One. 2022 Mar 31;17(3):e0265062. doi: 10.1371/journal.pone.0265062 (PMC8970397; doi:10.1371/journal.pone.0265062)
Supplement: S7 Table — (DOCX) [file pone.0265062.s010.docx]

**S7 Table. Most abundantly expressed endometrial genes in the cows after intrauterine infusion of pathogenic bacteria.**

| Gene ID | Symbol | Total read count | Type | Description |
| --- | --- | --- | --- | --- |
| 112444681 | *LOC112444681* | 5671146 | pseudo | 28S ribosomal RNA |
| 493779 | *RN18S1* | 4984803 | rRNA | 18S ribosomal RNA |
| 112442408 | *LOC112442408* | 850197 | protein-coding | translation initiation factor IF-2-like |
| 281850 | *IGHG1* | 767866 | other | immunoglobulin heavy constant gamma 1 |
| 100498812 | *MIR2887-1* | 715869 | ncRNA | microRNA 2887-1 |
| 104976804 | *LOC104976804* | 681736 | ncRNA | uncharacterized LOC104976804 |
| 282220 | *EEF1A1* | 613568 | protein-coding | eukaryotic translation elongation factor 1 alpha 1 |
| 510833 | *COL3A1* | 519490 | protein-coding | collagen type III alpha 1 chain |
| 539515 | *SRRM2* | 476649 | protein-coding | serine/arginine repetitive matrix 2 |
| 511422 | *COL6A1* | 463255 | protein-coding | collagen type VI alpha 1 chain |
| 281387 | *PENK* | 374814 | protein-coding | proenkephalin |
| 326599 | *TPT1* | 365374 | protein-coding | tumor protein, translationally-controlled 1 |
| 282194 | *COL6A2* | 341241 | protein-coding | collagen type VI alpha 2 chain |
| 282187 | *COL1A1* | 338835 | protein-coding | collagen type I alpha 1 chain |
| 786966 | *PLEC* | 334900 | protein-coding | plectin |
