# Supplementary material for: The endometrial transcriptomic response to pregnancy is altered in cows after uterine infection
Source: PLoS One. 2022 Mar 31;17(3):e0265062. doi: 10.1371/journal.pone.0265062 (PMC8970397; doi:10.1371/journal.pone.0265062)
Supplement: S8 Table — (DOCX) [file pone.0265062.s011.docx]

**S8 Table. Differentially expressed endometrial genes in pregnant cows compared to non-pregnant cows after intrauterine infusion with pathogenic bacteria.**

| Gene ID | Symbol | Type | Log_2_FC | Adj *P* Value |
| --- | --- | --- | --- | --- |
| 508284 | *ACBD7* | protein-coding | 1.485 | 4.37E-02 |
| 505134 | *ADAR* | protein-coding | 1.471 | 3.62E-07 |
| 327662 | *ANXA1* | protein-coding | 1.301 | 4.05E-03 |
| 506045 | *ATAD1* | protein-coding | 1.056 | 4.82E-02 |
| 100137953 | *ATP8B4* | protein-coding | 2.088 | 4.83E-06 |
| 522469 | *BATF2* | protein-coding | 1.678 | 3.54E-04 |
| 280734 | *BPI* | protein-coding | 1.664 | 1.14E-02 |
| 511581 | *C1R* | protein-coding | 1.152 | 1.07E-02 |
| 280678 | *C4A* | protein-coding | 1.145 | 1.13E-02 |
| 529166 | *CBLN3* | protein-coding | 1.598 | 1.10E-08 |
| 100848428 | *CCDC188* | protein-coding | -1.294 | 3.74E-02 |
| 101904723 | *CCDC194* | protein-coding | 1.54 | 9.64E-03 |
| 522998 | *CCDC6* | protein-coding | 0.894 | 2.54E-02 |
| 281044 | *CCL8* | protein-coding | 1.943 | 4.31E-04 |
| 104971522 | *CD160* | protein-coding | -0.982 | 4.52E-02 |
| 338319 | *CEBPB* | protein-coding | 0.796 | 2.57E-02 |
| 782472 | *CGAS* | protein-coding | 1.478 | 4.05E-03 |
| 784304 | *CMPK2* | protein-coding | 2.905 | 2.10E-13 |
| 509620 | *CMTR1* | protein-coding | 0.876 | 4.10E-02 |
| 281702 | *CNGB1* | protein-coding | 2.352 | 6.72E-06 |
| 280752 | *CNP* | protein-coding | 1.11 | 3.73E-02 |
| 519501 | *COL20A1* | protein-coding | -1.237 | 2.43E-02 |
| 513281 | *CPM* | protein-coding | 1.524 | 1.69E-02 |
| 534505 | *CPXM2* | protein-coding | 1.369 | 2.61E-02 |
| 281105 | *CTSB* | protein-coding | 0.919 | 4.55E-02 |
| 504760 | *DDX58* | protein-coding | 3.146 | 1.75E-21 |
| 508378 | *DHX58* | protein-coding | 2.279 | 1.27E-09 |
| 504445 | *DKK1* | protein-coding | 1.502 | 6.30E-04 |
| 515051 | *DTX3L* | protein-coding | 2.24 | 1.53E-06 |
| 522462 | *EFHD1* | protein-coding | 0.77 | 3.04E-02 |
| 347700 | *EIF2AK2* | protein-coding | 2.497 | 3.64E-11 |
| 614555 | *EPSTI1* | protein-coding | 2.896 | 2.17E-18 |
| 618755 | *FAM135B* | protein-coding | -1.917 | 4.41E-06 |
| 514701 | *FAM3B* | protein-coding | 2.058 | 2.06E-06 |
| 515085 | *FCRL3* | protein-coding | 1.506 | 3.83E-02 |
| 508090 | *FGL1* | protein-coding | 1.127 | 6.53E-03 |
| 788007 | *FLRT1* | protein-coding | -1.865 | 2.05E-04 |

S8 Table. Continued.

| Gene ID | Symbol | Type | Log_2_FC | Adj *P* Value |
| --- | --- | --- | --- | --- |
| 281797 | *GNGT2* | protein-coding | 1.736 | 8.23E-04 |
| 516026 | *GPRC5A* | protein-coding | 1.297 | 3.13E-02 |
| 510225 | *GRINA* | protein-coding | 1.169 | 7.79E-03 |
| 514373 | *HERC5* | protein-coding | 1.994 | 9.73E-07 |
| 527520 | *HERC6* | protein-coding | 2.781 | 2.57E-14 |
| 112442653 | *HOXB5* | protein-coding | -0.887 | 4.88E-02 |
| 506759 | *IFI16* | protein-coding | 2.261 | 2.44E-14 |
| 507138 | *IFI27* | protein-coding | 2.51 | 3.64E-11 |
| 508348 | *IFI44* | protein-coding | 3.223 | 9.88E-18 |
| 512913 | *IFI6* | protein-coding | 2.925 | 9.25E-13 |
| 535490 | *IFIH1* | protein-coding | 2.128 | 7.60E-09 |
| 515091 | *IFIT5* | protein-coding | 2.25 | 1.46E-11 |
| 777594 | *IFITM3* | protein-coding | 1.717 | 4.39E-07 |
| 282255 | *IFITM3(1-8U)* | protein-coding | 2.097 | 4.64E-06 |
| 509855 | *IRF9* | protein-coding | 1.757 | 2.12E-06 |
| 617420 | *ISG12(B)* | protein-coding | 1.723 | 7.25E-03 |
| 531137 | *LGALS3BP* | protein-coding | 1.307 | 1.37E-03 |
| 510813 | *LGALS9* | protein-coding | 1.424 | 5.38E-04 |
| 520564 | *LITAF* | protein-coding | 0.961 | 4.52E-02 |
| 100138660 | *LOC100138660* | ncRNA | -1.265 | 1.38E-02 |
| 100139670 | *LOC100139670* | protein-coding | 3.996 | 2.42E-21 |
| 100141258 | *LOC100141258* | protein-coding | 1.501 | 2.56E-02 |
| 100297676 | *LOC100297676* | protein-coding | 1.364 | 2.43E-02 |
| 100336669 | *LOC100336669* | protein-coding | 1.525 | 2.55E-03 |
| 100848246 | *LOC100848246* | ncRNA | -1.425 | 8.92E-03 |
| 100848263 | *LOC100848263* | protein-coding | 1.512 | 3.72E-02 |
| 101903402 | *LOC101903402* | ncRNA | 1.503 | 1.43E-03 |
| 101903765 | *LOC101903765* | pseudo | 1.96 | 7.33E-04 |
| 101904136 | *LOC101904136* | pseudo | -1.451 | 3.94E-02 |
| 101905897 | *LOC101905897* | ncRNA | -0.943 | 2.57E-02 |
| 101907799 | *LOC101907799* | ncRNA | 3.292 | 1.54E-15 |
| 104974749 | *LOC104974749* | ncRNA | -1.88 | 1.62E-03 |
| 104974750 | *LOC104974750* | ncRNA | -1.485 | 3.42E-02 |
| 104975106 | *LOC104975106* | pseudo | -1.658 | 5.13E-03 |
| 104975612 | *LOC104975612* | ncRNA | -1.596 | 1.21E-02 |
| 107132327 | *LOC107132327* | protein-coding | 1.721 | 1.34E-03 |
| 107132911 | *LOC107132911* | ncRNA | 1.427 | 1.69E-02 |
| 107133045 | *LOC107133045* | ncRNA | -1.494 | 2.30E-02 |

S8 Table. Continued.

| Gene ID | Symbol | Type | Log_2_FC | Adj *P* Value |
| --- | --- | --- | --- | --- |
| 112441507 | *LOC112441507* | protein-coding | 3.974 | 1.88E-22 |
| 112441868 | *LOC112441868* | ncRNA | -1.109 | 5.45E-03 |
| 112442254 | *LOC112442254* | ncRNA | -1.465 | 3.90E-02 |
| 112442264 | *LOC112442264* | ncRNA | -1.749 | 4.86E-03 |
| 112446427 | *LOC112446427* | protein-coding | 1.532 | 1.30E-04 |
| 112447832 | *LOC112447832* | ncRNA | -1.168 | 8.11E-03 |
| 112449099 | *LOC112449099* | protein-coding | -1.562 | 2.25E-02 |
| 509283 | *LOC509283* | protein-coding | 2.527 | 1.00E-13 |
| 510382 | *LOC510382* | pseudo | 3.738 | 4.77E-17 |
| 511531 | *LOC511531* | protein-coding | 1.532 | 1.30E-02 |
| 511937 | *LOC511937* | pseudo | 1.342 | 3.28E-02 |
| 512672 | *LOC512672* | protein-coding | 1.366 | 5.69E-03 |
| 514978 | *LOC514978* | protein-coding | 3.008 | 4.60E-14 |
| 614402 | *LOC614402* | protein-coding | 2.056 | 2.73E-04 |
| 618409 | *LOC618409* | protein-coding | 1.251 | 1.69E-02 |
| 618737 | *LOC618737* | protein-coding | 3.015 | 4.40E-11 |
| 789503 | *LOC789503* | protein-coding | -1.441 | 2.94E-03 |
| 790255 | *LOC790255* | protein-coding | -1.506 | 2.30E-02 |
| 515494 | *LRP10* | protein-coding | 0.883 | 1.88E-02 |
| 510977 | *LY6E* | protein-coding | 1.364 | 1.88E-02 |
| 505805 | *LY6G6C* | protein-coding | 1.506 | 2.57E-02 |
| 613856 | *LY86* | protein-coding | 1.281 | 5.10E-03 |
| 507845 | *MAPRE1* | protein-coding | 0.938 | 3.55E-02 |
| 100271851 | *MEF2B* | protein-coding | -1.662 | 1.15E-02 |
| 507921 | *MFSD5* | protein-coding | 1.084 | 1.61E-02 |
| 280857 | *MIA* | protein-coding | -1.276 | 4.31E-03 |
| 533051 | *MIC1* | protein-coding | 0.923 | 1.15E-02 |
| 790225 | *MLKL* | protein-coding | 1.5 | 5.10E-03 |
| 512308 | *MMRN2* | protein-coding | 1.202 | 5.38E-04 |
| 514667 | *MST1* | protein-coding | -0.941 | 2.09E-02 |
| 280872 | *MX1* | protein-coding | 3.544 | 1.19E-27 |
| 280873 | *MX2* | protein-coding | 4.386 | 1.58E-30 |
| 535092 | *MYO7A* | protein-coding | -1.475 | 4.96E-03 |
| 100125264 | *NUP210* | protein-coding | -1.036 | 1.10E-02 |
| 347699 | *OAS1X* | protein-coding | 3.194 | 3.10E-21 |
| 654488 | *OAS1Y* | protein-coding | 3.182 | 2.09E-21 |
| 519922 | *OAS1Z* | protein-coding | 2.883 | 9.71E-13 |
| 529660 | *OAS2* | protein-coding | 3.435 | 2.17E-19 |

S8 Table. Continued.

| Gene ID | Symbol | Type | Log_2_FC | Adj *P* Value |
| --- | --- | --- | --- | --- |
| 534150 | *OPTN* | protein-coding | 1.251 | 9.33E-03 |
| 514720 | *OSMR* | protein-coding | 1.088 | 2.89E-02 |
| 513185 | *PARP12* | protein-coding | 2.088 | 1.98E-10 |
| 540789 | *PARP14* | protein-coding | 2.742 | 3.54E-16 |
| 510532 | *PARP9* | protein-coding | 1.731 | 2.01E-07 |
| 538371 | *PAX5* | protein-coding | 1.707 | 8.08E-03 |
| 767910 | *PLAC8B* | protein-coding | 1.988 | 2.05E-04 |
| 510748 | *PLEKHA4* | protein-coding | 1.318 | 1.06E-02 |
| 280981 | *PLIN2* | protein-coding | 1.145 | 1.65E-02 |
| 100138545 | *PML* | protein-coding | 1.681 | 1.39E-05 |
| 538575 | *PRSS23* | protein-coding | 0.883 | 6.37E-03 |
| 617807 | *PSMF1* | protein-coding | 1.687 | 9.18E-06 |
| 541148 | *PTX3* | protein-coding | 1.677 | 3.06E-04 |
| 521304 | *RBFOX1* | protein-coding | -1.564 | 2.11E-02 |
| 617625 | *RBM43* | protein-coding | 1.101 | 7.42E-05 |
| 513479 | *RNF114* | protein-coding | 1.284 | 5.13E-03 |
| 281898 | *RPSA* | protein-coding | 1.244 | 2.57E-02 |
| 506415 | *RSAD2* | protein-coding | 4.446 | 2.98E-32 |
| 511675 | *RSPO1* | protein-coding | 1.27 | 4.97E-02 |
| 532442 | *RTP4* | protein-coding | 2.728 | 1.32E-12 |
| 514205 | *SAMD9* | protein-coding | 2.524 | 2.69E-09 |
| 504467 | *SASS6* | protein-coding | 0.903 | 4.06E-02 |
| 521133 | *SEC14L3* | protein-coding | 1.391 | 3.64E-02 |
| 286871 | *SERPINA14* | protein-coding | 1.516 | 3.41E-02 |
| 539321 | *SERTAD1* | protein-coding | 1.377 | 2.97E-03 |
| 617336 | *SHISA2* | protein-coding | 1.493 | 4.15E-02 |
| 616861 | *SHISA5* | protein-coding | 1.477 | 1.66E-04 |
| 539759 | *SIGLEC1* | protein-coding | 2.05 | 4.04E-07 |
| 317704 | *SLCO2B1* | protein-coding | 0.933 | 1.14E-02 |
| 616050 | *SMYD3* | protein-coding | 0.87 | 4.88E-02 |
| 514207 | *SNED1* | protein-coding | -1.047 | 1.13E-02 |
| 515204 | *SP110* | protein-coding | 2.19 | 2.54E-14 |
| 510377 | *SP140* | protein-coding | 1.602 | 7.42E-05 |
| 100139208 | *SP140L* | protein-coding | 1.408 | 5.15E-03 |
| 539299 | *SPATS2L* | protein-coding | 1.065 | 3.85E-02 |
| 784460 | *SPIB* | protein-coding | 1.663 | 1.05E-02 |
| 531014 | *SPTBN5* | protein-coding | -0.95 | 4.64E-02 |
| 510814 | *STAT1* | protein-coding | 1.639 | 1.50E-03 |

S8 Table. Continued.

| Gene ID | Symbol | Type | Log_2_FC | Adj *P* Value |
| --- | --- | --- | --- | --- |
| 511023 | *STAT2* | protein-coding | 1.08 | 4.99E-03 |
| 540573 | *STC2* | protein-coding | 1.527 | 5.13E-03 |
| 540438 | *STOML1* | protein-coding | 0.703 | 3.83E-02 |
| 506702 | *TDRD7* | protein-coding | 1.397 | 3.57E-04 |
| 783855 | *TIFA* | protein-coding | 1.524 | 1.21E-02 |
| 445425 | *TKT* | protein-coding | 0.834 | 3.64E-02 |
| 515475 | *TMEM140* | protein-coding | 1.427 | 2.88E-03 |
| 101902667 | *TMEM210* | protein-coding | -1.267 | 3.92E-02 |
| 507215 | *TNFSF10* | protein-coding | 1.632 | 1.52E-04 |
| 510923 | *TRIM25* | protein-coding | 1.266 | 1.37E-02 |
| 539820 | *TRIM34* | protein-coding | 1.368 | 2.13E-03 |
| 514896 | *TRIM56* | protein-coding | 1.186 | 4.32E-03 |
| 497204 | *UBA7* | protein-coding | 2.442 | 4.37E-09 |
| 509471 | *UBE2L6* | protein-coding | 1.473 | 6.39E-03 |
| 282113 | *UPK1B* | protein-coding | 2.032 | 1.81E-04 |
| 515202 | *USP18* | protein-coding | 3.589 | 7.16E-28 |
| 509740 | *XAF1* | protein-coding | 2.597 | 4.21E-13 |
| 508333 | *ZBP1* | protein-coding | 2.565 | 3.04E-09 |
| 787099 | *ZCCHC2* | protein-coding | 1.284 | 2.01E-07 |
| 539807 | *ZNFX1* | protein-coding | 2.72 | 2.16E-16 |
