# Supplementary material for: The endometrial transcriptomic response to pregnancy is altered in cows after uterine infection
Source: PLoS One. 2022 Mar 31;17(3):e0265062. doi: 10.1371/journal.pone.0265062 (PMC8970397; doi:10.1371/journal.pone.0265062)
Supplement: S9 Table — (DOCX) [file pone.0265062.s012.docx]

**S9 Table. Canonical pathways and related genes altered in the endometrium of pregnant cows compared to the non-pregnant cows after intrauterine infusion of pathogenic bacteria.**

| Canonical Pathways | -Log  (*P* value) | z-score | Differentially expressed genes in pathway |
| --- | --- | --- | --- |
| Interferon Signaling | 10.4 | 2.646 | *IFI6, IFIT1, IFITM3, IRF9, MX1, OAS1, STAT1, STAT2* |
| Activation of IRF by Cytosolic Pattern Recognition Receptors | 8.35 | 1.414 | *ADAR, DDX58, DHX58, IFIH1, IRF9, STAT1, STAT2, ZBP1* |
| Role of Pattern Recognition Receptors in Recognition of Bacteria and Viruses | 4.35 | nd^a^ | *DDX58, EIF2AK2, IFIH1, OAS1, OAS2, PTX3, TNFSF10* |
| Necroptosis Signaling Pathway | 4.3 | 2.646 | *EIF2AK2, IRF9, MLKL, STAT1, STAT2, TNFSF10, ZBP1* |
| Role of PKR in Interferon Induction and Antiviral Response | 4.09 | 2.449 | *DDX58, EIF2AK2, IFIH1, IRF9, STAT1, STAT2* |
| Role of RIG1-like Receptors in Antiviral Innate Immunity | 3.83 | 1 | *DDX58, DHX58, IFIH1, TRIM25* |
| Retinoic acid Mediated Apoptosis Signaling | 3.31 | 2 | *PARP12, PARP14, PARP9, TNFSF10* |
| Death Receptor Signaling | 2.63 | 2 | *PARP12, PARP14, PARP9, TNFSF10* |
| UVA-Induced MAPK Signaling | 2.52 | nd | *PARP12, PARP14, PARP9, STAT1* |
| T Cell Exhaustion Signaling Pathway | 2.36 | 1.342 | *IRF4, IRF9, LGALS9, STAT1, STAT2* |
| Acute Phase Response Signaling | 2.32 | nd | *C1R, C4A/C4B, CEBPB, LBP, OSMR* |
| Role of JAK1, JAK2 and TYK2 in Interferon Signaling | 2.03 | nd | *STAT1, STAT2* |
| Role of JAK family kinases in IL-6-type Cytokine Signaling | 2 | nd | *OSMR, STAT1* |
| JAK/Stat Signaling | 1.89 | nd | *CEBPB, STAT1, STAT2* |
| Complement System | 1.67 | nd | *C1R, C4A/C4B* |
| Systemic Lupus Erythematosus In B Cell Signaling Pathway | 1.58 | 2.236 | *IFIH1, IRF9, STAT1, STAT2, TNFSF10* |
| Oncostatin M Signaling | 1.55 | nd | *OSMR, STAT1* |

S9 Table. Continued.

| Canonical Pathways | -Log  (*P* value) | z-score | Differentially expressed genes in pathway |
| --- | --- | --- | --- |
| BAG2 Signaling Pathway | 1.55 | nd | *CTSB, PSMF1* |
| iNOS Signaling | 1.51 | nd | *LBP, STAT1* |
| p38 MAPK Signaling | 1.45 | nd | *MEF2B, STAT1, TIFA* |
| Pentose Phosphate Pathway (Non-oxidative Branch) | 1.44 | nd | *TKT* |
| Phototransduction Pathway | 1.38 | nd | *CNGB1, GNGT2* |
| IL-12 Signaling and Production in Macrophages | 1.33 | nd | *CEBPB, MST1, STAT1* |

^a^nd means Ingenuity Pathway Analysis could not determine a z-score.
