# Supplementary material for: The endometrial transcriptomic response to pregnancy is altered in cows after uterine infection
Source: PLoS One. 2022 Mar 31;17(3):e0265062. doi: 10.1371/journal.pone.0265062 (PMC8970397; doi:10.1371/journal.pone.0265062)
Supplement: S10 Table — (DOCX) [file pone.0265062.s013.docx]

**S10 Table. Gene networks altered in the endometrium of pregnant cows compared to the non-pregnant cows after intrauterine infusion of pathogenic bacteria.**

| Gene network^a^ | Score^b^ | Molecules in network |
| --- | --- | --- |
| Connective Tissue Disorders, Immunological Disease, Inflammatory Disease | 60 | ADAR, BPI, CCL8, CD160, CGAS, CMTR1, cytochrome C, DTX3L, EPSTI1, HERC5, HERC6, Hsp27, Ifi27, IFITM3, Ifn gamma, Interferon alpha, LGALS9, LITAF, LRP10, MLKL, NFkB (complex), OPTN, PARP, PARP12, PARP14, RNF114, SAMD9, SEC14L3, SPATS2L, TMEM140, Tnf (family), TRIM56, UBE2L6, USP18, XAF1 |
| Dermatological Diseases and Conditions, Immunological Disease, Organismal Injury and Abnormalities | 46 | Akt, BCR (complex), Collagen type II, CYP2J2, DDX58, FAM3B, GBP1, GBP2, IFI27, IFI44, IFI6, IFIT1, IFN Beta, IgG1, IgG2b, Igm, Ikb, Interferon-α Induced, IRF4, JAK, JAK1/2, LY86, MHC CLASS I (family), MX1, MX2, PARP9, PAX5, PLAC8, SIGLEC1, SP110, SP140, SPIB, TIFA, TKT, TNFSF10 |
| Antimicrobial Response, Infectious Diseases, Inflammatory Response | 38 | 2' 5' oas, Alpha 1 antitrypsin, C1R, C4A/C4B, CCDC6, Complement component 1, DHX58, ERK1/2, IFIH1, IFN alpha/beta, IFN type 1, Ifnar, IRF9, Isg, ISGF3, LBP, MIA, MST1, MUC5B, nucleotidyltransferase, Oas, OAS1, OAS2, OSMR, PRSS23, PTX3, RSAD2, RTP4, SAA, Serine Protease, STAT-1/2, Stat1-Stat2, STAT2, UBA7, ZBP1 |
| Antimicrobial Response, Cell Signaling, Inflammatory Response | 26 | 26s Proteasome, Alp, AMPK, BATF2, BST2, caspase, Cbp/p300, CEBPB, CMPK2, Creb, CTSB, Cyclin A, Cyclin E, EIF2AK2, ERK, Hdac, HISTONE, Hsp70, IFI16, IFIT5, Ifn, IRF, MICB, PDGF BB, Pias, PML, PP2A, PSMF1, Rb, RBFOX1, RPSA, STAT1, STAT5a/b, TRIM6-TRIM34, Ubiquitin |
| Inflammatory Response, Molecular Transport, Small Molecule Biochemistry | 26 | ACBD7, ADGRF3, ATAD1, beta-estradiol, chemokine, CX3CR1, FAM135B, FGL1, GRINA, H2-T24, IFNG, IL1B, IL6, KAT5, LILRA4, LOC100911216/Pcsk1, MEF2B, MFSD5, MMRN2, MX, MX2, PLEKHA4, progesterone, SASS6, SIGLEC1, SNED1, SPAG17, SRC, TCFL5, TNFRSF1A, TRIM25, TRPV1, VDR, WDPCP, Wfdc17 |
| Cell Cycle, Cell Death and Survival, Nervous System Development and Function | 22 | APP, CALML3, CBLN1, CBLN3, CHURC1, CNP, CNTN3, CPM, E2F1, EFHD1, EPB41L4A, ERMN, ESR2, FLRT1, FLRT3, GIMAP8, HAGH, HSPD1, LY6G6C, MAPRE1, MIB1, PCDH17, RBM43, RNF123, RNF213, SERTAD1, SLFN13, SNX31, STOML1, TBC1D30, TDRD7, TP63, UPK1B, UPK2, ZNF740 |

S10 Table. Continued.

| Gene network^a^ | Score^b^ | Molecules in network |
| --- | --- | --- |
| Lipid Metabolism, Molecular Transport, Small Molecule Biochemistry | 20 | Alpha catenin, CG, Ck2, CNGB1, DKK1, FSH, GPRC5A, Gsk3, Histone h3, Histone h4, HOXB5, IgG, IKK (complex), Insulin, LGALS3BP, Lh, MYO7A, PI3K (complex), PI3K (family), Pka, Pkc(s), PRC2, RAS, RNA polymerase II, Rnr, RSPO1, SHISA2, SLCO2B1, SMYD3, SRC (family), STAT, STC2, TCR, Vegf, ZNFX1 |
| Connective Tissue Disorders, Lipid Metabolism, Small Molecule Biochemistry | 10 | ANXA1, Ap1, CD3, COL20A1, collagen, collagen type i (family), Collagen(s), cytokine, estrogen receptor, FCRL3, Growth hormone, Hsp90, Ige, IL1, IL12 (complex), IL12 (family), Immunoglobulin, Jnk, LDL, LY6E, Mapk, Mek, MHC Class I (complex), MHC Class II (complex), N-cor, Nfat (family), Nr1h, P38 MAPK, PLAAT3, PLIN2, Pro-inflammatory Cytokine, Rxr, SHISA5, Tgf beta, Tlr |
| Infectious Diseases, Molecular Transport, Post-Translational Modification | 10 | ADGRL1, ATP11A, ATP1B3, ATP8B4, CANX, CNNM2, DDX60L, GNB1, GNGT2, GNPTAB, HS6ST1, MCF2L, NDC1, NUP210, NUP37, OPRD1, PIGT, PRSS21, SDF2, SECTM1, SLFN12, SPATS2L, SPCS3, SPTBN5, ST3GAL2, STT3A, TEX2, TMED7, TMEM30A, TMTC3, TOR1A, UBE4A, UQCRB, VIRMA, ZCCHC2 |
| Endocrine System Development and Function, Endocrine System Disorders, Nervous System Development and Function | 2 | ARNT2, CPXM2, OTX2, SIM1 |

^a^ Enriched gene networks determined by Ingenuity Pathway Analysis using significantly differentially expressed genes only.

^b^ Network score is derived from a P value and indicates the likelihood of the genes in a network being found together due to random chance. A network score of 2 or greater gives a 99% confidence the network and genes not being generated by random chance alone.
