# Supplementary material for: The endometrial transcriptomic response to pregnancy is altered in cows after uterine infection
Source: PLoS One. 2022 Mar 31;17(3):e0265062. doi: 10.1371/journal.pone.0265062 (PMC8970397; doi:10.1371/journal.pone.0265062)
Supplement: S11 Table — (DOCX) [file pone.0265062.s014.docx]

**S11 Table. Predicted upstream regulators identified in the endometrium of pregnant cows compared to the non-pregnant cows after intrauterine infusion of pathogenic bacteria.**

| Upstream Regulator | Type of molecule | Predicted state | z-score | *P*-value | Target molecules in dataset |
| --- | --- | --- | --- | --- | --- |
| IFNG | cytokine | Activated | 7.075 | 2.54E-26 | *BATF2, BST2, C1R, C4A/C4B, CCL8, CEBPB, CGAS, CMPK2, CTSB, DDX58, DKK1, DTX3L, EIF2AK2, GBP1, GBP2, HERC6, IFI16, IFI27, IFI44, IFI6, IFIH1, IFIT1, IFIT5, IFITM3, IRF4, IRF9, LGALS3BP, LGALS9, LY6E, MLKL, MX1, MX2, OAS1, OAS2, OPTN, PARP14, PARP9, PLAAT3, PML, PSMF1, PTX3, RNF114, RSAD2, RTP4, SAMD9, SP110, STAT1, STAT2, TNFSF10, UBE2L6, USP18, XAF1* |
| IFNA2 | cytokine | Activated | 6.207 | 1.60E-47 | *ANXA1, BST2, C1R, CCL8, CMPK2, CNP, DDX58, EIF2AK2, GBP1, GBP2, HERC5, HERC6, IFI16, IFI27, IFI44, IFI6, IFIH1, IFIT1, IFIT5, IFITM3, IRF9, LGALS3BP, LY6E, MX1, MX2, OAS1, OAS2, PARP12, PARP9, PML, RSAD2, SAMD9, SP110, STAT1, TDRD7, TNFSF10, UBA7, UBE2L6, USP18, XAF1, ZBP1* |
| PRL | cytokine | Activated | 6.037 | 1.79E-38 | *ADAR, BST2, CMPK2, CTSB, DDX58, DHX58, DTX3L, EIF2AK2, EPSTI1, HERC5, HERC6, IFI44, IFI6, IFIH1, IFIT1, IFIT5, IRF9, LY6E, MLKL, MX2, OAS1, OAS2, PARP12, PARP14, RPSA, RSAD2, SAMD9, SHISA5, SP110, STAT1, STAT2, TDRD7, TMEM140, TRIM25, USP18, XAF1, ZCCHC2* |

S11 Table. Continued.

| Upstream Regulator | Type of molecule | Predicted state | z-score | *P*-value | Target molecules in dataset |
| --- | --- | --- | --- | --- | --- |
| IRF7 | transcription regulator | Activated | 5.605 | 3.68E-40 | *ADAR, CCL8, CMPK2, DDX58, DHX58, GBP1, HERC5, IFI16, IFI44, IFI6, IFIH1, IFIT1, IFITM3, IRF9, MICB, MX1, MX2, OAS1, OAS2, PARP12, PARP14, PLAC8, RSAD2, RTP4, STAT1, STAT2, TDRD7, TNFSF10, UBA7, UBE2L6, USP18, XAF1, ZBP1* |
| Interferon alpha | group | Activated | 5.589 | 2.68E-44 | *ADAR, BST2, CGAS, CMTR1, DDX58, DHX58, EIF2AK2, EPSTI1, GBP1, GBP2, HERC5, HERC6, IFI16, IFI27, Ifi27, IFI44, IFI6, IFIH1, IFIT1, IFITM3, IRF4, IRF9, LGALS9, MX1, MX2, OAS1, OAS2, PARP12, PARP14, PARP9, PML, RNF213, RSAD2, RTP4, SAMD9, SEC14L3, SIGLEC1, SP110, STAT1, STAT2, TDRD7, TMEM140, TNFSF10, UBA7, UBE2L6, USP18, ZBP1* |
| IFNL1 | cytokine | Activated | 5.536 | 2.17E-49 | *BST2, CMPK2, DDX58, EIF2AK2, GBP1, HERC5, HERC6, IFI27, IFI44, IFI6, IFIH1, IFIT1, IFIT5, IFITM3, IRF9, LGALS3BP, MLKL, MX1, OAS1, OAS2, PML, RSAD2, RTP4, SAMD9, SP110, STAT1, STAT2, TDRD7, TMEM140, UBE2L6, USP18, XAF1* |
| IRF3 | transcription regulator | Activated | 5.062 | 2.56E-28 | *ADAR, CMPK2, DDX58, DHX58, EIF2AK2, GBP1, IFI16, IFI27, IFI44, IFI6, IFIH1, IFIT1, IFITM3, OAS1, OAS2, PARP12, PARP14, PLAC8, PML, RSAD2, STAT1, STAT2, TDRD7, TNFSF10, UBE2L6, USP18, ZBP1* |

S11 Table. Continued.

| Upstream Regulator | Type of molecule | Predicted state | z-score | *P*-value | Target molecules in dataset |
| --- | --- | --- | --- | --- | --- |
| STAT1 | transcription regulator | Activated | 4.872 | 8.70E-29 | *BATF2, BST2, C1R, C4A/C4B, CMPK2, EIF2AK2, EPSTI1, GBP1, GBP2, HERC6, IFI16, IFI27, IFI44, IFI6, IFIH1, IFIT1, IFITM3, IRF9, LY6E, MX1, OAS1, OAS2, PARP9, RNF213, RSAD2, RTP4, SP110, STAT1, STAT2, TNFSF10, USP18, XAF1, ZBP1* |
| IFNB1 | cytokine | Activated | 4.804 | 3.61E-25 | *BST2, CMPK2, DDX58, DHX58, EIF2AK2, GBP2, HERC5, IFI16, IFI27, IFI6, IFIH1, IFIT1, IRF9, MX1, OAS1, OAS2, PARP12, PARP14, PML, RSAD2, STAT1, STAT2, TNFSF10, TRIM6-TRIM34, UBA7, USP18, XAF1, ZBP1* |
| TNF | cytokine | Activated | 4.73 | 2.55E-14 | *ANXA1, BST2, C4A/C4B, CEBPB, CNP, CTSB, DDX58, DKK1, EIF2AK2, GBP1, GBP2, HERC5, IFI16, IFI27, IFI6, IFIH1, IFIT1, IFIT5, IRF4, LBP, LGALS9, LITAF, MIA, MST1, MX1, OAS1, OAS2, OPTN, OSMR, PARP14, PLAAT3, PLIN2, PML, PRSS23, PTX3, RPSA, SAMD9, SLCO2B1, STAT1, TDRD7, TIFA, TNFSF10, TRIM56* |
| IFN Beta | group | Activated | 4.687 | 5.50E-26 | *BST2, CEBPB, DDX58, EIF2AK2, HERC5, IFI16, IFI27, IFI44, IFI6, IFIH1, IFIT1, IRF9, MX1, MX2, OAS1, OAS2, RSAD2, STAT1, STAT2, TNFSF10, USP18, XAF1, ZBP1* |
| IRF1 | transcription regulator | Activated | 4.679 | 2.95E-26 | *C1R, CMPK2, DDX58, EIF2AK2, GBP2, IFI27, IFI44, IFI6, IFIH1, IFIT1, IFIT5, IFITM3, IRF4, IRF9, MX1, OAS1, OAS2, PLAAT3, PML, RSAD2, SP110, STAT1, STAT2, TNFSF10, XAF1* |

S11 Table. Continued.

| Upstream Regulator | Type of molecule | Predicted state | z-score | *P*-value | Target molecules in dataset |
| --- | --- | --- | --- | --- | --- |
| Ifnar | group | Activated | 4.165 | 4.37E-22 | *DDX58, EIF2AK2, GBP2, IFI16, IFIH1, IFITM3, IRF9, OAS1, OAS2, RNF213, RSAD2, STAT1, STAT2, TNFSF10, UBE2L6, USP18, XAF1, ZBP1* |
| IFNA1/IFNA13 | cytokine | Activated | 3.914 | 1.51E-22 | *CCL8, DHX58, EIF2AK2, IFI27, IFI6, IFIH1, IFIT1, MX1, OAS1, OAS2, RSAD2, SIGLEC1, STAT1, STAT2, UBE2L6, ZBP1* |
| APP | other | Activated | 3.906 | 1.97E-06 | *C1R, C4A/C4B, CMPK2, CTSB, DDX58, DKK1, GBP2, HERC6, IFI16, IFIH1, IRF4, LBP, PARP14, PAX5, RNF213, RSAD2, RTP4, TNFSF10, USP18, XAF1* |
| RNY3 | other | Activated | 3.873 | 6.86E-27 | *BATF2, EPSTI1, HERC5, IFI44, IFIT1, IFITM3, LY6E, MX1, OAS1, OAS2, RSAD2, RTP4, SIGLEC1, SPATS2L, XAF1* |
| TGM2 | enzyme | Activated | 3.86 | 1.63E-11 | *IFI6, IFIT1, IFIT5, IRF9, LGALS9, LY6E, OAS1, OAS2, PARP14, PARP9, RNF213, SP110, STAT1, UBA7, XAF1* |
| IRF5 | transcription regulator | Activated | 3.791 | 2.34E-19 | *CMPK2, DDX58, DHX58, IFI44, IFIH1, IFIT1, IFITM3, OAS1, OAS2, PARP12, RSAD2, SP110, STAT1, STAT2, TNFSF10, UBE2L6* |
| EIF2AK2 | kinase | Activated | 3.705 | 4.96E-17 | *CEBPB, DDX58, EIF2AK2, IFI27, IFI6, IFIT1, IFIT5, LGALS3BP, OAS1, PARP12, PARP9, SP140, STAT1, UBE2L6, USP18* |

S11 Table. Continued.

| Upstream Regulator | Type of molecule | Predicted state | z-score | *P*-value | Target molecules in dataset |
| --- | --- | --- | --- | --- | --- |
| SPI1 | transcription regulator | Activated | 3.695 | 1.27E-11 | *CMPK2, IFI27, IFI44, IFI6, IFIT1, IFITM3, IRF4, IRF9, LY6E, MX1, PARP12, PML, RSAD2, SP110, TNFSF10, USP18* |
| MAVS | other | Activated | 3.691 | 2.27E-19 | *ADAR, CGAS, CMPK2, DDX58, DHX58, IFIT1, IFITM3, OAS1, OAS2, PARP12, RSAD2, STAT1, STAT2, UBE2L6, USP18* |
| IL1B | cytokine | Activated | 3.669 | 5.79E-11 | *ANXA1, C1R, CCL8, CEBPB, CMPK2, CTSB, GBP1, GBP2, HERC5, IFI16, IFIT1, IRF4, LBP, LGALS9, MEF2B, MIA, MUC5B, MX1, OAS2, OSMR, PTX3, RPSA, RSAD2, STAT1, TNFSF10, UBE2L6, USP18* |
| Ifn | group | Activated | 3.53 | 7.25E-13 | *DDX58, DHX58, EIF2AK2, IFI16, IFIH1, IFIT1, IFITM3, MX1, OAS2, PML, RSAD2, STAT1, ZBP1* |
| PML | transcription regulator | Activated | 3.44 | 6.13E-10 | *BST2, EPSTI1, HERC6, IFI27, IFI44, IFIH1, IFIT1, MX1, OAS1, OAS2, PML, STAT1* |
| TLR9 | transmembrane receptor | Activated | 3.302 | 4.84E-09 | *CPM, IFI16, IFIT1, IRF4, IRF9, MX1, MX2, OAS2, RSAD2, STAT1, STAT2, TNFSF10, USP18* |
| IFN type 1 | group | Activated | 3.264 | 5.10E-18 | *BST2, CGAS, DDX58, DHX58, EIF2AK2, IFI16, IFIH1, IFIT1, PML, STAT1, STAT2, TNFSF10, UBA7* |
| TLR3 | transmembrane receptor | Activated | 3.26 | 1.89E-17 | *CMPK2, CPM, DDX58, DHX58, EIF2AK2, GBP2, HERC5, IFI16, IFI44, IFI6, IFIH1, IFIT1, MX1, MX2, OAS1, PTX3, RSAD2, STAT1, TNFSF10, USP18, ZNFX1* |

S11 Table. Continued.

| Upstream Regulator | Type of molecule | Predicted state | z-score | *P*-value | Target molecules in dataset |
| --- | --- | --- | --- | --- | --- |
| SMARCA4 | transcription regulator | Activated | 3.24 | 4.59E-05 | *CEBPB, CNP, CPM, CTSB, GBP1, IFI16, IFI27, IFIT1, IFITM3, MICB, PLIN2, PTX3, SERTAD1, TNFSF10* |
| TLR7 | transmembrane receptor | Activated | 3.138 | 1.28E-08 | *CEBPB, DKK1, IFI44, IFIT1, IRF9, MX1, MX2, OAS2, PTX3, RSAD2, STAT1, STAT2* |
| IL6 | cytokine | Activated | 3.073 | 1.33E-04 | *ANXA1, BST2, CEBPB, FGL1, GBP2, IFI16, IFIT1, IFITM3, IRF4, IRF9, LBP, LY86, SP110, STAT1, TNFSF10* |
| CD3 | complex | Activated | 3.022 | 2.69E-06 | *ANXA1, BST2, C1R, GBP1, GPRC5A, IFIT1, IRF4, IRF9, MST1, PLIN2, PSMF1, STAT1, TNFSF10, TRIM25, UBE2L6, XAF1* |
| ELAVL1 | other | Activated | 3 | 6.78E-09 | *CCL8, IFI16, Ifi27, IFI44, IFIH1, IFITM3, IRF9, LGALS3BP, OAS1, OAS2, STAT1, USP18* |
| IFNAR1 | transmembrane receptor | Activated | 2.969 | 9.90E-17 | *CGAS, CMPK2, EIF2AK2, IFI16, IFI44, IFI6, IFIH1, MX2, OAS1, OAS2, PARP12, RSAD2, RTP4, STAT1, TNFSF10, USP18, XAF1* |
| TNFSF10 | cytokine | Activated | 2.95 | 1.62E-08 | *CTSB, EIF2AK2, IFI16, IFI27, IFI6, IFIT1, IRF9, STAT1, TNFSF10* |
| IFNA4 | cytokine | Activated | 2.93 | 1.05E-11 | *CCL8, GBP2, H2-T24, IFIH1, IFIT1, MX1, RSAD2, USP18, ZBP1* |
| FADD | other | Activated | 2.828 | 9.01E-08 | *DDX58, DHX58, EIF2AK2, IFIH1, LY6E, RNF114, STAT1, STAT2* |

S11 Table. Continued.

| Upstream Regulator | Type of molecule | Predicted state | z-score | *P*-value | Target molecules in dataset |
| --- | --- | --- | --- | --- | --- |
| JAK | group | Activated | 2.828 | 2.36E-10 | *DDX58, EIF2AK2, IFI6, IFIH1, IFIT1, IFITM3, RSAD2, STAT1* |
| IFNE | cytokine | Activated | 2.795 | 2.36E-10 | *BST2, HERC5, IFIH1, IFITM3, MX2, STAT1, USP18, ZBP1* |
| IL27 | cytokine | Activated | 2.788 | 3.98E-06 | *BST2, EIF2AK2, GBP2, MX1, OAS1, STAT1, STAT2, TNFSF10* |
| Ifn gamma | complex | Activated | 2.777 | 2.07E-07 | *ADAR, EIF2AK2, GBP1, LGALS9, PML, STAT1, TNFSF10, XAF1* |
| TLR4 | transmembrane receptor | Activated | 2.752 | 1.09E-06 | *BPI, CCL8, CMPK2, GBP2, IFI16, IFITM3, MX1, PML, PTX3, RSAD2, STAT1, STAT2, TNFSF10* |
| IL21 | cytokine | Activated | 2.714 | 1.59E-08 | *CMPK2, EIF2AK2, HERC6, IFI16, IFIT1, IRF4, OAS2, PAX5, RSAD2, STAT2, USP18* |
| PAF1 | other | Activated | 2.646 | 2.26E-08 | *DDX58, HERC5, IFI44, IFITM3, OAS2, SERTAD1, ZNFX1* |
| IFNL4 | cytokine | Activated | 2.624 | 3.87E-14 | *DDX58, DHX58, IFIH1, MX1, OAS1, OAS2, STAT1* |
| NFkB (complex) | complex | Activated | 2.591 | 7.71E-04 | *C1R, CCL8, CEBPB, GBP2, HERC5, IRF4, LITAF, MUC5B, PTX3, RSAD2, SPIB, TNFSF10* |
| STING1 | other | Activated | 2.586 | 4.35E-09 | *CGAS, IFI16, IFI44, IFITM3, OAS1, PLEKHA4, RSAD2, USP18* |
| DDX58 | enzyme | Activated | 2.541 | 7.35E-13 | *DDX58, EIF2AK2, IFI27, IFI44, IFIH1, IFIT1, OAS1, RSAD2, STAT1, STAT2, TNFSF10* |

S11 Table. Continued.

| Upstream Regulator | Type of molecule | Predicted state | z-score | *P*-value | Target molecules in dataset |
| --- | --- | --- | --- | --- | --- |
| IFNAR2 | transmembrane receptor | Activated | 2.449 | 1.12E-18 | *DDX58, HERC5, IFI44, IFI6, IFIH1, MX2, OAS1, OAS2, TNFSF10, UBA7, UBE2L6, USP18, XAF1* |
| IFNK | cytokine | Activated | 2.449 | 5.05E-09 | *EIF2AK2, IFIH1, MX1, OAS1, STAT1, ZBP1* |
| JAK1 | kinase | Activated | 2.449 | 2.83E-07 | *EIF2AK2, IRF9, MX1, OSMR, STAT1, STAT2, USP18* |
| MSC | transcription regulator | Activated | 2.449 | 1.52E-06 | *EPSTI1, IFI27, IFI44, IFIT1, PAX5, XAF1* |
| SMARCB1 | transcription regulator | Activated | 2.449 | 7.68E-04 | *C4A/C4B, EIF2AK2, IFI16, LBP, MX1, OAS1* |
| IKBKG | kinase | Activated | 2.438 | 2.38E-04 | *CEBPB, CTSB, GBP2, IFI16, PTX3, TNFSF10* |
| STAT4 | transcription regulator | Activated | 2.433 | 5.57E-04 | *DDX58, IFIH1, IRF4, PLAC8, SERTAD1, STAT1, STC2* |
| IKBKB | kinase | Activated | 2.423 | 9.91E-03 | *CEBPB, CTSB, GBP2, IFI16, MX1, PTX3* |
| TICAM1 | other | Activated | 2.408 | 3.11E-04 | *CMPK2, DDX58, IFI16, IFIT1, RSAD2, TNFSF10* |
| IFN alpha/ beta | group | Activated | 2.39 | 1.21E-05 | *IFI16, LY6E, RSAD2, STAT1, STAT2, TNFSF10* |
| OSM | cytokine | Activated | 2.268 | 1.29E-05 | *ANXA1, C1R, CPM, GBP1, GBP2, IRF9, LBP, LITAF, LY6G6C, MX1, OAS1, OSMR, STAT1, UBE2L6* |
| DUSP1 | phosphatase | Activated | 2.236 | 8.30E-04 | *CMPK2, DKK1, IFIT1, MX1, PLIN2* |
| SAMSN1 | other | Activated | 2.236 | 2.73E-04 | *CMPK2, PML, RSAD2, STAT1, STAT2* |

S11 Table. Continued.

| Upstream Regulator | Type of molecule | Predicted state | z-score | *P*-value | Target molecules in dataset |
| --- | --- | --- | --- | --- | --- |
| SASH1 | other | Activated | 2.236 | 8.44E-05 | *CMPK2, PML, RSAD2, STAT1, STAT2* |
| SNCA | enzyme | Activated | 2.236 | 5.48E-02 | *CTSB, GBP2, MX1, PLAC8, RSAD2* |
| IFNL3 | cytokine | Activated | 2.23 | 9.50E-07 | *DDX58, MX1, RSAD2, STAT1, USP18* |
| IL1A | cytokine | Activated | 2.219 | 2.09E-02 | *C4A/C4B, CCL8, GBP1, LGALS9, PTX3* |
| JAK1/2 | group | Activated | 2.219 | 8.44E-05 | *EIF2AK2, GBP2, MX1, PLAC8, RSAD2* |
| PARP9 | enzyme | Activated | 2.219 | 7.25E-08 | *IFI44, IFIT1, OAS2, SP110, STAT1* |
| IRF9 | transcription regulator | Activated | 2.213 | 9.41E-15 | *GBP1, IFI27, IFIT1, IFITM3, MX1, OAS2, RTP4, STAT1, STAT2, TNFSF10, ZBP1* |
| CHUK | kinase | Activated | 2.211 | 1.11E-02 | *CEBPB, CTSB, GBP2, IFI16, PTX3* |
| IFNA5 | cytokine | Activated | 2.2 | 1.73E-08 | *CCL8, IFIH1, IFIT1, MX1, ZBP1* |
| IFNA6 | cytokine | Activated | 2.2 | 1.73E-08 | *CCL8, IFIH1, IFIT1, MX1, ZBP1* |
| IFNA7 | cytokine | Activated | 2.2 | 1.73E-08 | *CCL8, IFIH1, IFIT1, MX1, ZBP1* |
| IFNA8 | cytokine | Activated | 2.2 | 2.58E-08 | *CCL8, IFIH1, IFIT1, MX1, ZBP1* |
| IFNA10 | cytokine | Activated | 2.2 | 1.73E-08 | *CCL8, IFIH1, IFIT1, MX1, ZBP1* |
| IFNA14 | cytokine | Activated | 2.2 | 1.73E-08 | *CCL8, IFIH1, IFIT1, MX1, ZBP1* |
| IFNA16 | cytokine | Activated | 2.2 | 2.58E-08 | *CCL8, IFIH1, IFIT1, MX1, ZBP1* |
| IFNA21 | cytokine | Activated | 2.2 | 1.73E-08 | *CCL8, IFIH1, IFIT1, MX1, ZBP1* |
| CGAS | enzyme | Activated | 2.199 | 4.34E-07 | *IFI44, IFIT1, OAS1, RSAD2, USP18* |

S11 Table. Continued.

| Upstream Regulator | Type of molecule | Predicted state | z-score | *P*-value | Target molecules in dataset |
| --- | --- | --- | --- | --- | --- |
| REL | transcription regulator | Activated | 2.169 | 3.95E-03 | *IRF4, PARP14, PLAAT3, RSAD2, SASS6, TNFSF10* |
| P38 MAPK | group | Activated | 2.024 | 2.61E-03 | *BATF2, CCL8, CEBPB, GBP1, MUC5B, PML, STAT1, TNFSF10* |
| Brd4 | kinase | Activated | 2 | 1.21E-03 | *C1R, H2-T24, LY86, TNFSF10* |
| DOCK8 | other | Activated | 2 | 9.34E-04 | *CMPK2, RSAD2, STAT1, STAT2* |
| SYVN1 | transporter | Activated | 2 | 1.24E-02 | *GPRC5A, HERC5, IFI44, LGALS3BP* |
| TNK1 | kinase | Activated | 2 | 3.31E-06 | *IFI16, IFIH1, OAS2, TNFSF10* |
| MAPK1 | kinase | Inhibited | -4.919 | 8.52E-27 | *ADAR, BST2, CTSB, DDX58, EIF2AK2, GBP1, GBP2, HERC5, IFI16, IFI27, IFI44, IFI6, IFIH1, IFIT1, IFIT5, IFITM3, IRF9, LGALS3BP, MX2, NUP210, OAS1, OAS2, PARP12, PML, SP110, STAT1, STAT2, TDRD7, TNFSF10, TRIM25, UBE2L6, USP18* |
| NKX2-3 | transcription regulator | Inhibited | -4.796 | 1.05E-21 | *BATF2, CMPK2, DDX58, DHX58, EIF2AK2, GBP1, GBP2, LY6E, PARP12, PARP14, PARP9, PLEKHA4, RNF213, RTP4, SAMD9, SP110, STAT1, STAT2, UBA7, UBE2L6, USP18, XAF1, ZNFX1* |
| IL1RN | cytokine | Inhibited | -4.583 | 5.76E-24 | *DDX58, GBP1, HERC6, IFI27, IFI44, IFI6, IFIH1, IFIT5, IRF9, LGALS9, MX1, MX2, OAS1, OAS2, PML, RSAD2, RTP4, SAMD9, STAT2, TNFSF10, USP18* |

S11 Table. Continued.

| Upstream Regulator | Type of molecule | Predicted state | z-score | *P*-value | Target molecules in dataset |
| --- | --- | --- | --- | --- | --- |
| TRIM24 | transcription regulator | Inhibited | -4.416 | 1.05E-23 | *CMPK2, DDX58, DHX58, EPSTI1, GBP2, HERC6, IFI44, IFIH1, IRF9, LGALS3BP, OAS1, PARP12, PLAC8, RTP4, SHISA5, STAT1, STAT2, TRIM6-TRIM34, UBA7, USP18* |
| PNPT1 | enzyme | Inhibited | -4.333 | 1.02E-29 | *CMPK2, DDX58, EIF2AK2, GBP2, IFI16, IFI44, IFIH1, LGALS3BP, OAS1, PARP12, PARP14, PARP9, RNF213, RTP4, STAT1, STAT2, UBE2L6, USP18, XAF1* |
| RC3H1 | enzyme | Inhibited | -4.243 | 1.70E-24 | *BST2, DDX58, IFI16, IFI27, IFI44, IFI6, IFIT1, IFITM3, IRF9, MX1, OAS1, OAS2, PARP9, RSAD2, STAT1, STAT2, TRIM25, TRIM56* |
| SIRT1 | transcription regulator | Inhibited | -4.121 | 1.16E-14 | *ADAR, CEBPB, CMPK2, DDX58, DHX58, DKK1, IFI44, IFITM3, LGALS3BP, LY6E, OAS1, OAS2, PARP12, PARP14, PML, RNF213, RSAD2, RTP4, SP110, STAT1, UBA7, USP18* |
| PTGER4 | G-protein coupled receptor | Inhibited | -3.568 | 1.26E-11 | *CMPK2, DDX58, GBP2, HERC6, IFI16, IFIH1, PARP14, RNF213, RSAD2, RTP4, TNFSF10, USP18, XAF1* |
| SOCS1 | other | Inhibited | -3.552 | 5.75E-12 | *DDX58, H2-T24, IFI16, IFI27, IFI44, IFIH1, IFIT1, MX1, OAS1, OAS2, RSAD2, STAT1, USP18* |
| ACKR2 | G-protein coupled receptor | Inhibited | -3.464 | 2.97E-17 | *ADAR, DDX58, DHX58, EIF2AK2, IFI16, IFI44, OAS1, OAS2, RSAD2, STAT1, STAT2, USP18* |

S11 Table. Continued.

| Upstream Regulator | Type of molecule | Predicted state | z-score | *P*-value | Target molecules in dataset |
| --- | --- | --- | --- | --- | --- |
| SP110 | transcription regulator | Inhibited | -3.317 | 1.46E-09 | *BST2, CTSB, IFI27, IFI6, IFIH1, IFIT1, IFITM3, IRF9, MX1, OAS1, STAT1* |
| mir-21 | microRNA | Inhibited | -3.111 | 1.31E-05 | *C1R, DHX58, GBP2, IFI16, OAS2, SIGLEC1, STAT1, STAT2, UBA7, UBE2L6* |
| IL4 | cytokine | Inhibited | -2.977 | 1.68E-05 | *ANXA1, CCL8, CMPK2, DHX58, EIF2AK2, GBP2, IFI16, IFI44, IFIH1, IFITM3, IRF4, IRF9, LGALS3BP, PAX5, PLIN2, RNF213, STAT1, STAT2, ZBP1* |
| IRF4 | transcription regulator | Inhibited | -2.971 | 6.18E-08 | *GBP1, IRF4, IRF9, MAPRE1, OAS1, PAX5, SPIB, STAT1, STAT2, TNFSF10* |
| NRAS | enzyme | Inhibited | -2.804 | 5.56E-07 | *GBP2, IFI16, Ifi27, IFIH1, IFIT1, LBP, LY86, PTX3, STAT1, USP18* |
| IKZF3 | transcription regulator | Inhibited | -2.646 | 3.12E-07 | *DDX58, DKK1, IFI27, IFI6, IFIT5, RNF213, RTP4* |
| USP18 | peptidase | Inhibited | -2.588 | 7.61E-10 | *IFI6, IFIH1, IFITM3, IRF9, MX1, OAS1, TNFSF10* |
| IL10RA | transmembrane receptor | Inhibited | -2.53 | 2.85E-05 | *BATF2, GBP2, IFI16, MLKL, PLAAT3, RNF213, RSAD2, SLCO2B1, STAT1, ZBP1* |
| KRAS | enzyme | Inhibited | -2.514 | 2.14E-05 | *ADAR, CEBPB, EIF2AK2, IFI6, IFIT1, IFITM3, IRF9, MICB, MX1, MX2, OAS1, RPSA, STAT1, STAT2, TNFSF10* |
| STAT6 | transcription regulator | Inhibited | -2.492 | 1.56E-09 | *ANXA1, CCL8, CMPK2, CTSB, DHX58, EIF2AK2, GBP2, IFI16, IFI44, IFIH1, IFITM3, IRF4, IRF9, LGALS3BP, RNF213, STAT2, ZBP1* |

S11 Table. Continued.

| Upstream Regulator | Type of molecule | Predicted state | z-score | *P*-value | Target molecules in dataset |
| --- | --- | --- | --- | --- | --- |
| miR-199a-5p (and other miRNAs w/seed CCAGUGU) | mature microRNA | Inhibited | -2.449 | 7.13E-06 | *CMPK2, GPRC5A, IFI27, MX2, RSAD2, ZBP1* |
| TAB1 | enzyme | Inhibited | -2.433 | 3.54E-08 | *GBP1, GBP2, IFIH1, IFIT1, TNFSF10, XAF1* |
| IKZF1 | transcription regulator | Inhibited | -2.419 | 6.69E-08 | *CYP2J2, DKK1, DTX3L, EPSTI1, IFI16, IFI27, IFI6, IFIT5, IRF4, PAX5, RNF213, RTP4* |
| TREX1 | enzyme | Inhibited | -2.405 | 1.97E-09 | *IFI16, IFI44, IFIT1, MX1, OAS1, USP18* |
| NGLY1 | enzyme | Inhibited | -2.386 | 7.64E-08 | *IFI27, IFI44, IFIT1, OAS1, RSAD2, USP18* |
| MYC | transcription regulator | Inhibited | -2.261 | 1.45E-07 | *CNP, CTSB, DKK1, GBP2, HERC5, IFI16, IFI27, IFI44, IFI6, IFIH1, IFIT1, IFIT5, IRF4, IRF9, MX1, MX2, OAS1, PAX5, PML, RPSA, RSAD2, STAT1, TKT, TNFSF10, USP18* |
| PIK3CG | kinase | Inhibited | -2.236 | 5.51E-04 | *GBP2, OAS2, STAT1, TNFSF10, ZBP1* |
| ISG15 | other | Inhibited | -2.222 | 1.88E-10 | *DDX58, IFI6, IFITM3, MX1, OAS1* |
| GAPDH | enzyme | Inhibited | -2.2 | 2.91E-06 | *IFI6, OAS1, OAS2, STAT1, UBE2L6* |
| mir-155 | microRNA | Inhibited | -2.198 | 9.31E-04 | *CEBPB, IRF4, IRF9, MX1, STAT1* |
| PRDM1 | transcription regulator | Inhibited | -2.184 | 2.30E-04 | *CD160, IRF4, PAX5, PLAC8, RSAD2, SLCO2B1, SPIB, TNFSF10* |

S11 Table. Continued.

| Upstream Regulator | Type of molecule | Predicted state | z-score | *P*-value | Target molecules in dataset |
| --- | --- | --- | --- | --- | --- |
| CLDN7 | other | Inhibited | -2.157 | 1.10E-06 | *DKK1, GRINA, IFI44, IFI6, MX1, PLAAT3, PRSS23, UBE2L6* |
| BCL6 | transcription regulator | Inhibited | -2.156 | 1.69E-05 | *CGAS, HERC5, HERC6, IRF4, IRF9, LITAF, PRSS23, STAT1, UBA7* |
| BTK | kinase | Inhibited | -2.121 | 7.03E-07 | *CEBPB, IFIT1, IRF4, IRF9, MX1, MX2, OAS2, STAT1* |
| Irgm1 | other | Inhibited | -2 | 1.97E-04 | *IFI16, OAS2, RSAD2, USP18* |
| SAMHD1 | enzyme | Inhibited | -2 | 1.01E-06 | *DDX58, IFI27, IFI6, MX1* |
