# Supplementary material for: The endometrial transcriptomic response to pregnancy is altered in cows after uterine infection
Source: PLoS One. 2022 Mar 31;17(3):e0265062. doi: 10.1371/journal.pone.0265062 (PMC8970397; doi:10.1371/journal.pone.0265062)
Supplement: S12 Table — (DOCX) [file pone.0265062.s015.docx]

**S12 Table. Differentially expressed endometrial genes in pregnant cows compared to non-pregnant cows after intrauterine infusion with pathogenic bacteria (Log_2_FC ≥ 1.5 or ≤ -1.5).**

| Gene ID | Symbol | Type | Log2FC | *Adj P Value* |
| --- | --- | --- | --- | --- |
| 100137953 | *ATP8B4* | protein-coding | 2.088 | 4.83E-06 |
| 522469 | *BATF2* | protein-coding | 1.678 | 3.54E-04 |
| 280734 | *BPI* | protein-coding | 1.664 | 1.14E-02 |
| 529166 | *CBLN3* | protein-coding | 1.598 | 1.10E-08 |
| 101904723 | *CCDC194* | protein-coding | 1.54 | 9.64E-03 |
| 281044 | *CCL8* | protein-coding | 1.943 | 4.31E-04 |
| 784304 | *CMPK2* | protein-coding | 2.905 | 2.10E-13 |
| 281702 | *CNGB1* | protein-coding | 2.352 | 6.72E-06 |
| 513281 | *CPM* | protein-coding | 1.524 | 1.69E-02 |
| 504760 | *DDX58* | protein-coding | 3.146 | 1.75E-21 |
| 508378 | *DHX58* | protein-coding | 2.279 | 1.27E-09 |
| 504445 | *DKK1* | protein-coding | 1.502 | 6.30E-04 |
| 515051 | *DTX3L* | protein-coding | 2.24 | 1.53E-06 |
| 347700 | *EIF2AK2* | protein-coding | 2.497 | 3.64E-11 |
| 614555 | *EPSTI1* | protein-coding | 2.896 | 2.17E-18 |
| 618755 | *FAM135B* | protein-coding | -1.917 | 4.41E-06 |
| 514701 | *FAM3B* | protein-coding | 2.058 | 2.06E-06 |
| 515085 | *FCRL3* | protein-coding | 1.506 | 3.83E-02 |
| 788007 | *FLRT1* | protein-coding | -1.865 | 2.05E-04 |
| 281797 | *GNGT2* | protein-coding | 1.736 | 8.23E-04 |
| 514373 | *HERC5* | protein-coding | 1.994 | 9.73E-07 |
| 527520 | *HERC6* | protein-coding | 2.781 | 2.57E-14 |
| 506759 | *IFI16* | protein-coding | 2.261 | 2.44E-14 |
| 507138 | *IFI27* | protein-coding | 2.51 | 3.64E-11 |
| 508348 | *IFI44* | protein-coding | 3.223 | 9.88E-18 |
| 512913 | *IFI6* | protein-coding | 2.925 | 9.25E-13 |
| 535490 | *IFIH1* | protein-coding | 2.128 | 7.60E-09 |
| 515091 | *IFIT5* | protein-coding | 2.25 | 1.46E-11 |
| 777594 | *IFITM3* | protein-coding | 1.717 | 4.39E-07 |
| 282255 | *IFITM3(1-8U)* | protein-coding | 2.097 | 4.64E-06 |
| 509855 | *IRF9* | protein-coding | 1.757 | 2.12E-06 |
| 617420 | *ISG12(B)* | protein-coding | 1.723 | 7.25E-03 |
| 100139670 | *LOC100139670* | protein-coding | 3.996 | 2.42E-21 |
| 100141258 | *LOC100141258* | protein-coding | 1.501 | 2.56E-02 |
| 100336669 | *LOC100336669* | protein-coding | 1.525 | 2.55E-03 |
| 100848263 | *LOC100848263* | protein-coding | 1.512 | 3.72E-02 |
| 101903402 | *LOC101903402* | ncRNA | 1.503 | 1.43E-03 |

S12 Table. Continued.

| Gene ID | Symbol | Type | Log2FC | *Adj P Value* |
| --- | --- | --- | --- | --- |
| 101903765 | *LOC101903765* | pseudo | 1.96 | 7.33E-04 |
| 101907799 | *LOC101907799* | ncRNA | 3.292 | 1.54E-15 |
| 104974749 | *LOC104974749* | ncRNA | -1.88 | 1.62E-03 |
| 104975106 | *LOC104975106* | pseudo | -1.658 | 5.13E-03 |
| 104975612 | *LOC104975612* | ncRNA | -1.596 | 1.21E-02 |
| 107132327 | *LOC107132327* | protein-coding | 1.721 | 1.34E-03 |
| 112441507 | *LOC112441507* | protein-coding | 3.974 | 1.88E-22 |
| 112442264 | *LOC112442264* | ncRNA | -1.749 | 4.86E-03 |
| 112446427 | *LOC112446427* | protein-coding | 1.532 | 1.30E-04 |
| 112449099 | *LOC112449099* | protein-coding | -1.562 | 2.25E-02 |
| 509283 | *LOC509283* | protein-coding | 2.527 | 1.00E-13 |
| 510382 | *LOC510382* | pseudo | 3.738 | 4.77E-17 |
| 511531 | *LOC511531* | protein-coding | 1.532 | 1.30E-02 |
| 514978 | *LOC514978* | protein-coding | 3.008 | 4.60E-14 |
| 614402 | *LOC614402* | protein-coding | 2.056 | 2.73E-04 |
| 618737 | *LOC618737* | protein-coding | 3.015 | 4.40E-11 |
| 790255 | *LOC790255* | protein-coding | -1.506 | 2.30E-02 |
| 505805 | *LY6G6C* | protein-coding | 1.506 | 2.57E-02 |
| 100271851 | *MEF2B* | protein-coding | -1.662 | 1.15E-02 |
| 790225 | *MLKL* | protein-coding | 1.5 | 5.10E-03 |
| 280872 | *MX1* | protein-coding | 3.544 | 1.19E-27 |
| 280873 | *MX2* | protein-coding | 4.386 | 1.58E-30 |
| 347699 | *OAS1X* | protein-coding | 3.194 | 3.10E-21 |
| 654488 | *OAS1Y* | protein-coding | 3.182 | 2.09E-21 |
| 519922 | *OAS1Z* | protein-coding | 2.883 | 9.71E-13 |
| 529660 | *OAS2* | protein-coding | 3.435 | 2.17E-19 |
| 513185 | *PARP12* | protein-coding | 2.088 | 1.98E-10 |
| 540789 | *PARP14* | protein-coding | 2.742 | 3.54E-16 |
| 510532 | *PARP9* | protein-coding | 1.731 | 2.01E-07 |
| 538371 | *PAX5* | protein-coding | 1.707 | 8.08E-03 |
| 767910 | *PLAC8B* | protein-coding | 1.988 | 2.05E-04 |
| 100138545 | *PML* | protein-coding | 1.681 | 1.39E-05 |
| 617807 | *PSMF1* | protein-coding | 1.687 | 9.18E-06 |
| 541148 | *PTX3* | protein-coding | 1.677 | 3.06E-04 |
| 521304 | *RBFOX1* | protein-coding | -1.564 | 2.11E-02 |
| 506415 | *RSAD2* | protein-coding | 4.446 | 2.98E-32 |
| 532442 | *RTP4* | protein-coding | 2.728 | 1.32E-12 |
| 514205 | *SAMD9* | protein-coding | 2.524 | 2.69E-09 |

S12 Table. Continued.

| Gene ID | Symbol | Type | Log2FC | *Adj P Value* |
| --- | --- | --- | --- | --- |
| 286871 | *SERPINA14* | protein-coding | 1.516 | 3.41E-02 |
| 539759 | *SIGLEC1* | protein-coding | 2.05 | 4.04E-07 |
| 515204 | *SP110* | protein-coding | 2.19 | 2.54E-14 |
| 510377 | *SP140* | protein-coding | 1.602 | 7.42E-05 |
| 784460 | *SPIB* | protein-coding | 1.663 | 1.05E-02 |
| 510814 | *STAT1* | protein-coding | 1.639 | 1.50E-03 |
| 540573 | *STC2* | protein-coding | 1.527 | 5.13E-03 |
| 783855 | *TIFA* | protein-coding | 1.524 | 1.21E-02 |
| 507215 | *TNFSF10* | protein-coding | 1.632 | 1.52E-04 |
| 497204 | *UBA7* | protein-coding | 2.442 | 4.37E-09 |
| 282113 | *UPK1B* | protein-coding | 2.032 | 1.81E-04 |
| 515202 | *USP18* | protein-coding | 3.589 | 7.16E-28 |
| 509740 | *XAF1* | protein-coding | 2.597 | 4.21E-13 |
| 508333 | *ZBP1* | protein-coding | 2.565 | 3.04E-09 |
| 539807 | *ZNFX1* | protein-coding | 2.72 | 2.16E-16 |
