# Supplementary material for: The endometrial transcriptomic response to pregnancy is altered in cows after uterine infection
Source: PLoS One. 2022 Mar 31;17(3):e0265062. doi: 10.1371/journal.pone.0265062 (PMC8970397; doi:10.1371/journal.pone.0265062)
Supplement: S13 Table — (DOCX) [file pone.0265062.s016.docx]

**S13 Table. Differentially expressed genes in the endometrium of pregnant cows compared to non-pregnant cows in all studies (Log_2_FC compared to non-pregnant).**

| Gene ID | Symbol | Type | Infected | Healthy | Healthy | Healthy |
| --- | --- | --- | --- | --- | --- | --- |
|  |  |  | d 16 | d 15 | d 16 | d 17 |
| 504445 | *DKK1* | protein-coding | 1.5 | 2.05 | 2.79 | 1.98 |
| 347700 | *EIF2AK2* | protein-coding | 2.49 | 2.98 | 5.51 | 2.97 |
| 506759 | *IFI16* | protein-coding | 2.26 | 2.48 | 5.18 | 2.85 |
| 507138 | *IFI27* | protein-coding | 2.51 | 3.08 | 6.38 | 2.55 |
| 512913 | *IFI6* | protein-coding | 2.93 | 2.46 | 8.77 | 2.79 |
| 535490 | *IFIH1* | protein-coding | 2.13 | 2.44 | 5.57 | 2.71 |
| 515091 | *IFIT5* | protein-coding | 2.25 | 2.2 | 5.41 | 2.25 |
| 509855 | *IRF9* | protein-coding | 1.76 | 2.56 | 4.87 | 2.73 |
| 509283 | *LOC509283* | protein-coding | 3.74 | 2.92 | 5.98 | 3.48 |
| 280872 | *MX1* | protein-coding | 3.54 | 3.64 | 14.69 | 3.18 |
| 280873 | *MX2* | protein-coding | 4.39 | 5.32 | 52.59 | 7.81 |
| 513185 | *PARP12* | protein-coding | 2.09 | 2.12 | 3.2 | 2.39 |
| 540789 | *PARP14* | protein-coding | 2.74 | 2.53 | 6.32 | 3.13 |
| 510532 | *PARP9* | protein-coding | 1.73 | 1.8 | 3.65 | 2.26 |
| 767910 | *PLAC8B* | protein-coding | 1.99 | 2.28 | 6.86 | 3.09 |
| 506415 | *RSAD2* | protein-coding | 4.45 | 4.84 | 36.62 | 5 |
| 532442 | *RTP4* | protein-coding | 2.73 | 3.34 | 5.5 | 3.83 |
| 539759 | *SIGLEC1* | protein-coding | 2.05 | 2.27 | 4.86 | 4.91 |
| 510814 | *STAT1* | protein-coding | 1.64 | 1.69 | 3.72 | 1.51 |
| 783855 | *TIFA* | protein-coding | 1.52 | 1.63 | 3.3 | 2.96 |
| 497204 | *UBA7* | protein-coding | 2.44 | 3.27 | 8.62 | 3.8 |
| 515202 | *USP18* | protein-coding | 3.59 | 3.64 | 14.55 | 4.33 |
| 509740 | *XAF1* | protein-coding | 2.6 | 2.69 | 6.62 | 3.25 |
| 539807 | *ZNFX1* | protein-coding | 2.72 | 2.77 | 5.26 | 3.01 |
| 101904723 | *CCDC194* | protein-coding | 1.54 |  |  |  |

S13 Table. Continued.

| Gene ID | Symbol | Type | Infected | Healthy | Healthy | Healthy |
| --- | --- | --- | --- | --- | --- | --- |
|  |  |  | d 16 | d 15 | d 16 | d 17 |
| 281702 | *CNGB1* | protein-coding | 2.35 |  |  |  |
| 513281 | *CPM* | protein-coding | 1.52 |  |  |  |
| 618755 | *FAM135B* | protein-coding | -1.92 |  |  |  |
| 515085 | *FCRL3* | protein-coding | 1.51 |  |  |  |
| 788007 | *FLRT1* | protein-coding | -1.87 |  |  |  |
| 777594 | *IFITM3* | protein-coding | 1.72 |  |  |  |
| 282255 | *IFITM3(1-8U)* | protein-coding | 2.1 |  |  |  |
| 100141258 | *LOC100141258* | protein-coding (uncharacterized) | 1.5 |  |  |  |
| 100336669 | *LOC100336669* | protein-coding (GBP4) | 1.53 |  |  |  |
| 100848263 | *LOC100848263* | protein-coding (SLFN12) | 1.51 |  |  |  |
| 101903402 | *LOC101903402* | ncRNA | 1.5 |  |  |  |
| 101903765 | *LOC101903765* | pseudo | 1.96 |  |  |  |
| 101907799 | *LOC101907799* | ncRNA | 3.29 |  |  |  |
| 104974749 | *LOC104974749* | ncRNA | -1.88 |  |  |  |
| 104975106 | *LOC104975106* | pseudo | -1.66 |  |  |  |
| 104975612 | *LOC104975612* | ncRNA | -1.6 |  |  |  |
| 107132327 | *LOC107132327* | protein-coding (CYP2J2L) | 1.72 |  |  |  |
| 112442264 | *LOC112442264* | ncRNA | -1.75 |  |  |  |
| 112446427 | *LOC112446427* | protein-coding (uncharacterized) | 1.53 |  |  |  |
| 112449099 | *LOC112449099* | protein-coding (uncharacterized) | -1.56 |  |  |  |
| 614402 | *LOC614402* | protein-coding (HRASLS3) | 2.06 |  |  |  |
| 790255 | *LOC790255* | protein-coding (LILRA6) | -1.51 |  |  |  |
| 100271851 | *MEF2B* | protein-coding | -1.66 |  |  |  |
| 538371 | *PAX5* | protein-coding | 1.71 |  |  |  |
| 521304 | *RBFOX1* | protein-coding | -1.56 |  |  |  |
| 784460 | *SPIB* | protein-coding | 1.66 |  |  |  |

S13 Table. Continued.

| Gene ID | Symbol | Type | Infected | | Healthy | Healthy | Healthy |
| --- | --- | --- | --- | --- | --- | --- | --- |
|  |  |  | d 16 | | d 15 | d 16 | d 17 |
| 540573 | *STC2* | protein-coding | 1.53 | |  |  |  |
| 510774 | *ABHD1* | protein-coding | |  | 1.51 | 2.31 | 3.21 |
| 505134 | *ADAR* | protein-coding | |  | 1.65 | 2.81 | 2.08 |
| 511001 | *CLEC4F* | protein-coding | |  | 2.93 | 77.35 | 8.66 |
| 615107 | *CXCL10* | protein-coding | |  | 2.95 | 4.55 | 3.53 |
| 613313 | *GBP4* | protein-coding | |  | 2.59 | 7.11 | 2.69 |
| 508347 | *IFI44L* | protein-coding | |  | 3.83 | 18.09 | 5.09 |
| 281871 | *ISG15* | protein-coding | |  | 5.24 | 54.69 | 5.07 |
| 506604 | *ISG20* | protein-coding | |  | 3.19 | 13.44 | 7.14 |
| 508877 | *PNPT1* | protein-coding | |  | 2.39 | 3.41 | 2.95 |
| 280701 | *PPA1* | protein-coding | |  | 1.64 | 2.45 | 2.38 |
| 507549 | *TIMD4* | protein-coding | |  | 1.64 | 3.01 | 4.14 |
| 509859 | *TRANK1* | protein-coding | |  | 1.57 | 2.57 | 2.35 |
