# Supplementary material for: The endometrial transcriptomic response to pregnancy is altered in cows after uterine infection
Source: PLoS One. 2022 Mar 31;17(3):e0265062. doi: 10.1371/journal.pone.0265062 (PMC8970397; doi:10.1371/journal.pone.0265062)
Supplement: S14 Table — Infected day 16, Healthy day 15, Healthy day 16, Healthy day 17. (DOCX) [file pone.0265062.s017.docx]

**S14 Table. Altered canonical pathways in the endometrium of pregnant cows compared to non-pregnant cows in all studies. Infected day 16, Healthy day 15, Healthy day 16, Healthy day 17.**

|  | Bacteria infused day 16 | | Healthy day 15 | | Healthy day 16 | | Healthy day 17 | |
| --- | --- | --- | --- | --- | --- | --- | --- | --- |
| Canonical Pathways | -Log_10_ (*P*)  z-score | Molecules | -Log_10_ (*P*)  z-score | Molecules | -Log_10_ (*P*)  z-score | Molecules | -Log_10_ (*P*)  z-score | Molecules |
| Interferon Signaling | 10.7  2.45 | *IFI6, IFIT1, IFITM3, IRF9, MX1, OAS1, STAT1* | 13.00  2.65 | *IFI6, IFIT1, IFITM1, IRF9, ISG15, MX1, OAS1, STAT1* | 13.8  3.16 | *IFI35, IFI6, IFIT1, IFIT3, IRF9, ISG15, MX1, OAS1, STAT1, STAT2, TAP1* | 14  2.53 | *IFI35, IFI6, IFITM1, IRF9, ISG15, MX1, OAS1, SOCS1, STAT1, STAT2, TAP1* |
| Activation of IRF by Cytosolic Pattern Recognition Receptors | 7.32  1.63 | *DDX58, DHX58, IFIH1, IRF9, STAT1, ZBP1* | 12.70  1.00 | *ADAR, DDX58, DHX58, IFIH1, IRF7, IRF9, ISG15, STAT1, ZBP1* | 9.54  1.90 | *ADAR, CD40, IFIH1, IFIT2, IRF3, IRF9, ISG15, STAT1, STAT2, ZBP1* | 11.1  1.51 | *ADAR, CD40, DDX58, DHX58, IFIH1, IFIT2, IRF7, IRF9, ISG15, STAT1, STAT2* |
| Role of PKR in Interferon Induction and Antiviral Response | 4.45  2.24 | *DDX58, EIF2AK2, IFIH1, IRF9, STAT1* | 4.61  2.24 | *DDX58, EIF2AK2, IFIH1, IRF9, STAT1* | 4.89  2.83 | *CASP8, EIF2AK2, FCGR1A, IFIH1, IRF3, IRF9, STAT1, STAT2* | 6.02  2.84 | *ATF3, DDX58, EIF2AK2, FCGR1A, IFIH1, IRF9, PYCARD, STAT1, STAT2* |
| Role of Pattern Recognition Receptors in Recognition of Bacteria and Viruses | 6.24  nd^a^ | *DDX58, EIF2AK2, IFIH1, OAS1, OAS2, PTX3, TNFSF10* | 5.21  2.00 | *DDX58, EIF2AK2, IFIH1, IRF7, OAS1, TNFSF10* | 6.71  2.24 | *C1QB, C1QC, C3AR1, EIF2AK2, IFIH1, IRF3, OAS1, OAS2, RNASEL, TNFSF10, TNFSF13B* | 5.03  2.45 | *C1QB, C1QC, DDX58, EIF2AK2, IFIH1, IRF7, OAS1, PTX3, TNFSF13B* |
| Systemic Lupus Erythematosus In B Cell Signaling Pathway | 2.74  2.24 | *IFIH1, IRF9, LILRA6, STAT1, TNFSF10* | 4.79  2.65 | *IFIH1, IRF7, IRF9, ISG15, ISG20, STAT1, TNFSF10* | 5.76  3.05 | *CCND1, CD40, IFIH1, IFIT2, IFIT3, IRF3, IRF9, ISG15, ISG20, STAT1, STAT2, TNFSF10, TNFSF13B* | 3.77  3.16 | *CD40, IFIH1, IFIT2, IRF7, IRF9, ISG15, ISG20, STAT1, STAT2, TNFSF13B* |

S14 Table. Continued.

|  | Bacteria infused day 16 | | Healthy day 15 | | Healthy day 16 | | Healthy day 17 | |
| --- | --- | --- | --- | --- | --- | --- | --- | --- |
| Canonical Pathways | -Log_10_ (*P*)  z-score | Molecules | -Log_10_ (*P*)  z-score | Molecules | -Log_10_ (*P*)  z-score | Molecules | -Log_10_ (*P*)  z-score | Molecules |
| Role of RIG1-like Receptors in Antiviral Innate Immunity | 3.44  nd | *DDX58, DHX58, IFIH1* | 5.07  1.00 | *DDX58, DHX58, IFIH1, IRF7* | 3.15  2.00 | *CASP8, IFIH1, IRF3, TRIM25* | 3.22  1.00 | *DDX58, DHX58, IFIH1, IRF7* |
| Coronavirus Pathogenesis Pathway | 1.92  nd | *DDX58, IRF9, STAT1* | 4.09  -2.24 | *DDX58, EIF4E, IRF7, IRF9, STAT1* | 3.31  -1.13 | *CASP8, CCND1, IRF3, IRF9, STAT1, STAT2, TRIM25* | 2.67  -1.63 | *DDX58, IRF7, IRF9, PYCARD, STAT1, STAT2* |
| Necroptosis Signaling Pathway | 4.97  2.45 | *EIF2AK2, IRF9, MLKL, STAT1, TNFSF10, ZBP1* | 4.00  2.24 | *EIF2AK2, IRF9, STAT1, TNFSF10, ZBP1* | 5.70  2.53 | *CASP8, EIF2AK2, IRF3, IRF9, MLKL, RIPK3, STAT1, STAT2, TNFSF10, ZBP1* | 2.57  2.45 | *EIF2AK2, IRF9, MLKL, PYCARD, STAT1, STAT2* |
| UVA-Induced MAPK Signaling | 3.57  nd | *PARP12, PARP14, PARP9, STAT1* | 3.69  nd | *PARP12, PARP14, PARP9, STAT1* | 4.47  nd | *PARP10, PARP12, PARP14, PARP9, PLCL2, STAT1, TIPARP* | 1.96  nd | *PARP12, PARP14, PARP9, STAT1* |
| Retinoic acid Mediated Apoptosis Signaling | 4.4  2.00 | *PARP12, PARP14, PARP9, TNFSF10* | 4.53  2.00 | *PARP12, PARP14, PARP9, TNFSF10* | 5.89  2.65 | *CASP8, PARP10, PARP12, PARP14, PARP9, TIPARP, TNFSF10* | 1.80  nd | *PARP12, PARP14, PARP9* |
| Death Receptor Signaling | 3.70  2.00 | *PARP12, PARP14, PARP9, TNFSF10* | 3.82  2.00 | *PARP12, PARP14, PARP9, TNFSF10* | 4.68  2.65 | *CASP8, PARP10, PARP12, PARP14, PARP9, TIPARP, TNFSF10* | 1.33  nd | *PARP12, PARP14, PARP9* |

S14 Table. Continued.

|  | Bacteria infused day 16 | | Healthy day 15 | | Healthy day 16 | | | Healthy day 17 | | |
| --- | --- | --- | --- | --- | --- | --- | --- | --- | --- | --- |
| Canonical Pathways | -Log_10_ (*P*)  z-score | Molecules | -Log_10_ (*P*)  z-score | Molecules | -Log_10_ (*P*)  z-score | | Molecules | -Log_10_ (*P*)  z-score | | Molecules |
| iNOS Signaling | 2.05  nd | *LBP, STAT1* |  |  |  | |  |  | |  |
| Phototransduction Pathway | 1.91  nd | *CNGB1, GNGT2* |  |  |  | |  |  | |  |
| Toll-like receptor signaling | 1.62  nd | *EIF2AK2, LBP* |  |  |  | |  |  | |  |
| IL-7 signaling pathway | 1.60  nd | *PAX5, STAT1* |  |  |  | |  |  |  |  |
| Salvage pathways of pyrimidine ribonucleotides | 1.41  nd | *CMPK2, EIF2AK2* |  |  |  |  |  |  |  |  |

^a^nd means Ingenuity Pathway Analysis could not determine a z-score.
