# Supplementary material for: The endometrial transcriptomic response to pregnancy is altered in cows after uterine infection
Source: PLoS One. 2022 Mar 31;17(3):e0265062. doi: 10.1371/journal.pone.0265062 (PMC8970397; doi:10.1371/journal.pone.0265062)
Supplement: S15 Table — Predicted activation state was either activated (+) or inhibited (-). (DOCX) [file pone.0265062.s018.docx]

**S15 Table. Predicted upstream regulators in the endometrium of pregnant cows compared to non-pregnant cows using all studies. Predicted activation state was either activated (+) or inhibited (-).**

|  | Bacteria infused day 16 | | Healthy day 15 | | Healthy day 16 | | Healthy day 17 | |
| --- | --- | --- | --- | --- | --- | --- | --- | --- |
| Predicted upstream regulator | +/-  z -score  *P* value | Molecules | +/-  z -score  *P* value | Molecules | +/-  z -score  *P* value | Molecules | +/-  z -score  *P* value | Molecules |
| ACKR2 | -  -3.16  1.21E-16 | DDX58, DHX58, EIF2AK2, IFI16, IFI44, OAS1, OAS2, RSAD2, STAT1, USP18 | -  -3.74  9.28E-26 | ADAR, CXCL10, DDX58, DHX58, EIF2AK2, IFI16, IFI44, IRF7, ISG15, ISG20, OAS1, RSAD2, STAT1, USP18 | -  -3.87  3.53E-20 | ADAR, CCL11, CXCL10, EIF2AK2, IFI16, IFIT2, IFIT3, ISG15, ISG20, OAS1, OAS2, RSAD2, STAT1, STAT2, USP18 | -  -4.00  2.30E-22 | ADAR, CXCL10, DDX58, DHX58, EIF2AK2, IFI16, IFI44, IFIT2, IRF7, ISG15, ISG20, OAS1, RSAD2, STAT1, STAT2, USP18 |
| APP | +  3.78  5.41E-08 | CMPK2, DDX58, DKK1, GBP2, HERC6, IFI16, IFIH1, LBP, PARP14, PAX5, RNF213, RSAD2, RTP4, TNFSF10, USP18, XAF1 | +  3.90  3.35E-11 | C4A/C4B, CRYM, CXCL10, DDX58, DKK1, FABP3, GBP4, HERC6, IFI16, IFIH1, IRF7, ISG20, PARP14, RNF213, RSAD2, RTP4, TNFSF10, USP18, XAF1 | +  3.90  7.10E-12 | C1S, C4A/C4B, CCL11, CCND1, CD40, CMPK2, COL13A1, CXCL10, DKK1, FCGR1A, GBP2, GBP4, GBP6, HERC6, IDO1, IFI16, IFI35, IFIH1, IFIT2, IRF3, IRF4, ISG20, KYNU, MAPT, NAMPT, PARP14, RNASEL, RNF213, RNF24, RSAD2, RTP4, TNFSF10, TRIM21, USP18, XAF1 | +  3.49  6.42E-10 | CD40, CD69, CD86, CRYM, CXCL10, DDX58, DKK1, EPAS1, FABP3, FAP, FCGR1A, GBP4, H19, IDO1, IFI16, IFI35, IFIH1, IFIT2, IRF7, ISG20, MCM10, NCDN, PARP14, PYCARD, RNF213, RSAD2, RTP4, SHISA3, SOCS1, USP18, XAF1 |

S15 Table. Continued.

|  | Bacteria infused day 16 | | Healthy day 15 | | Healthy day 16 | | Healthy day 17 | |
| --- | --- | --- | --- | --- | --- | --- | --- | --- |
| Predicted upstream regulator | +/-  z -score  *P* value | Molecules | +/-  z -score  *P* value | Molecules | +/-  z -score  *P* value | Molecules | +/-  z -score  *P* value | Molecules |
| BTK | -  -2.45  2.13E-06 | IFIT1, IRF9, MX1, MX2, OAS2, STAT1 | -  -3.16  2.03E-12 | CXCL10, IFI44L, IFIT1, IFITM1, IRF9, ISG15, ISG20, MX1, MX2, STAT1 | -  -3.86  3.49E-13 | CD274, CD40, CXCL10, IFI35, IFI44L, IFIT1, IFIT3, IRF4, IRF9, ISG15, ISG20, MX1, MX2, OAS2, STAT1 | -  -3.42  2.76E-12 | CCR7, CD40, CD69, CD86, CXCL10, IFI35, IFI44L, IFITM1, IRF9, ISG15, ISG20, MX1, MX2, STAT1 |
| CD3 | +  2.55  1.08E-03 | BST2, GBP1, IFIT1, IRF9, PSMF1, STAT1, TNFSF10, XAF1 | +  2.52  4.25E-07 | BST2, CXCL10, EIF4E, GBP1, IFIT1, IFITM1, IRF9, PPA1, SP100, STAT1, TNFSF10, XAF1 | +  3.20  5.24E-08 | ANXA1, C2, CASP8, CCND1, CD274, CD53, COX7A1, CXCL10, GBP1, IFI35, IFIT1, IRF4, IRF9, MAP3K8, NAMPT, PPA1, PSMF1, STAT1, SYT7, TNFSF10, TRIM25, UBE2L6, XAF1 | +  3.18  2.16E-05 | ALAS1, C2, CD69, CD86, CREM, CXCL10, GBP1, H19, IFI35, IFITM1, IRF9, PPA1, PSMF1, SMPDL3B, SOCS1, STAT1, UBE2L6, XAF1 |
| CGAS | +  2.20  1.59E-08 | IFI44, IFIT1, OAS1, RSAD2, USP18 | +  2.97  2.40E-17 | CXCL10, IFI44, IFI44L, IFIT1, IRF7, ISG15, OAS1, RSAD2, USP18 | +  2.95  9.80E-13 | CXCL10, IFI44L, IFIT1, IFIT2, IFIT3, ISG15, OAS1, RSAD2, USP18 | +  2.97  6.04E-13 | CXCL10, IFI44, IFI44L, IFIT2, IRF7, ISG15, OAS1, RSAD2, USP18 |
| DDX58 | +  2.38  2.70E-14 | DDX58, EIF2AK2, IFI27, IFI44, IFIH1, IFIT1, OAS1, RSAD2, STAT1, TNFSF10 | +  3.04  2.68E-22 | CXCL10, DDX58, EIF2AK2, IFI27, IFI44, IFIH1, IFIT1, IRF7, ISG15, ISG20, OAS1, RSAD2, STAT1, TNFSF10 | +  3.63  1.52E-19 | CASP4, CXCL10, EIF2AK2, IFI27, IFI35, IFIH1, IFIT1, IFIT2, IFIT3, IRF3, ISG15, ISG20, OAS1, RSAD2, STAT1, STAT2, TNFSF10 | +  3.17  1.51E-21 | CASP4, CXCL10, DDX58, EIF2AK2, IFI27, IFI35, IFI44, IFIH1, IFIT2, IRF7, ISG15, ISG20, NMI, OAS1, RSAD2, SOCS1, STAT1, STAT2 |

S15 Table. Continued.

|  | Bacteria infused day 16 | | Healthy day 15 | | Healthy day 16 | | Healthy day 17 | |
| --- | --- | --- | --- | --- | --- | --- | --- | --- |
| Predicted upstream regulator | +/-  z -score  *P* value | Molecules | +/-  z -score  *P* value | Molecules | +/-  z -score  *P* value | Molecules | +/-  z -score  *P* value | Molecules |
| EIF2AK2 | +  3.43  3.46E-16 | DDX58, EIF2AK2, IFI27, IFI6, IFIT1, IFIT5, OAS1, PARP12, PARP9, SP140, STAT1, USP18 | +  3.82  4.61E-22 | DDX58, EIF2AK2, IFI27, IFI6, IFIT1, IFIT5, IFITM1, ISG15, ISG20, OAS1, PARP12, PARP9, SP140, STAT1, USP18 | +  3.82  1.25E-15 | CCND1, EIF2AK2, IFI27, IFI35, IFI6, IFIT1, IFIT5, ISG15, ISG20, LGALS3BP, OAS1, PARP12, PARP9, STAT1, UBE2L6, USP18 | +  4.28  2.84E-20 | ATF3, DDX58, EIF2AK2, IFI27, IFI35, IFI6, IFIT5, IFITM1, ISG15, ISG20, LGALS3BP, NMI, OAS1, PARP12, PARP9, SP140, STAT1, UBE2L6, USP18 |
| Ifn | +  3.53  1.07E-16 | DDX58, DHX58, EIF2AK2, IFI16, IFIH1, IFIT1, IFITM3, MX1, OAS2, PML, RSAD2, STAT1, ZBP1 | +  3.92  2.90E-24 | CXCL10, DDX58, DHX58, EIF2AK2, IFI16, IFIH1, IFIT1, IFITM1, IRF7, ISG15, ISG20, MX1, PML, RSAD2, SP100, STAT1, ZBP1 | +  4.57  1.44E-23 | B2M, CASP8, CD40, CXCL10, EIF2AK2, IDO1, IFI16, IFIH1, IFIT1, IL23A, IRF3, ISG15, ISG20, MX1, OAS2, PML, RNASEL, RSAD2, STAT1, TAP1, TNFSF13B, TRIM21, ZBP1 | +  4.15  3.16E-21 | CD40, CD58, CD69, CD86, CXCL10, DDX58, DHX58, EIF2AK2, IDO1, IFI16, IFIH1, IFITM1, IRF7, ISG15, ISG20, MX1, RSAD2, SOCS1, STAT1, TAP1, TNFSF13B |
| IFN Beta | +  4.59  2.71E-31 | BST2, CMPK2, DDX58, EIF2AK2, HERC5, IFI16, IFI27, IFI44, IFI6, IFIH1, IFIT1, IRF9, MX1, MX2, OAS1, OAS2, RSAD2, STAT1, TNFSF10, USP18, XAF1, ZBP1 | +  4.70  4.87E-36 | BST2, CXCL10, DDX58, EIF2AK2, IFI16, IFI27, IFI44, IFI6, IFIH1, IFIT1, IFITM1, IRF7, IRF9, ISG15, MX1, MX2, OAS1, PNPT1, RSAD2, STAT1, TNFSF10, USP18, XAF1, ZBP1 | +  5.45  1.32E-34 | C3AR1, CASP4, CD274, CD40, CMPK2,CXCL10,EIF2AK2,HERC5,IDO1, IFI16,IFI27, IFI35,IFI6,IFIH1,IFIT1,IFIT2,IFIT3,IRF9,ISG15,MX1,MX2,OAS1,OAS2,PNPT1,RSAD2,STAT1,STAT2,TNFSF10, USP18, USP25, XAF1, ZBP1 | +  5.01  7.85E-34 | ATF3, CASP4, CCR7, CD40, CD69, CD86, CXCL10, DDX58, EIF2AK2, IDO1,IFI16,IFI27,IFI35,IFI44,IFI6,IFIH1,IFIT2,IFITM1,IRF7,IRF9,ISG15,MX1,MX2,OAS1,PNPT1,RSAD2,SOCS1,STAT1,STAT2,USP18,XAF1 |

S15 Table. Continued.

|  | Bacteria infused day 16 | | Healthy day 15 | | Healthy day 16 | | Healthy day 17 | |
| --- | --- | --- | --- | --- | --- | --- | --- | --- |
| Predicted upstream regulator | +/-  z -score  *P* value | Molecules | +/-  z -score  *P* value | Molecules | +/-  z -score  *P* value | Molecules | +/-  z -score  *P* value | Molecules |
| Ifn gamma | +  2.42  8.41E-07 | EIF2AK2, GBP1, PML, STAT1, TNFSF10, XAF1 | +  2.95  1.70E-11 | ADAR,CXCL10,EIF2AK2,GBP1,IFI44L,PML,STAT1,TNFSF10,XAF1 | +  3.26  2.75E-09 | ADAR,CXCL10,EIF2AK2,GBP1,IFI44L,LGALS9,PML,PSME2,STAT1,TNFSF10,XAF1 | +  2.48  2.24E-08 | ADAR,CD86,CXCL10,EIF2AK2,GBP1,IFI44L,LGALS9,SOCS1,STAT1,XAF1 |
| IFN type 1 | +  3.11  8.25E-18 | BST2, DDX58, DHX58, EIF2AK2, IFI16, IFIH1, IFIT1, PML, STAT1, TNFSF10, UBA7 | +  3.37  2.26E-22 | BST2,CXCL10,DDX58,DHX58,EIF2AK2,IFI16,IFIH1,IFIT1,ISG15,PML,STAT1,TNFSF10,UBA7 | +  3.38  6.95E-19 | CGAS,CXCL10,EIF2AK2,IDO1,IFI16,IFIH1,IFIT1,IFIT2,ISG15,PML,STAT1,STAT2,TNFSF10,TNFSF13B,UBA7 | +  3.38  1.47E-17 | CD69,CXCL10,DDX58,DHX58,EIF2AK2,IDO1,IFI16,IFIH1,IFIT2,ISG15,STAT1,STAT2,TNFSF13B,UBA7 |
| IFNA1/ IFNA13 | +  3.66  3.47E-23 | CCL8, DHX58, EIF2AK2, IFI27, IFI6, IFIH1, IFIT1, MX1, OAS1, OAS2, RSAD2, SIGLEC1, STAT1, ZBP1 | +  4.01  2.97E-30 | CCL8,CXCL10,DHX58,EIF2AK2,IFI27,IFI6,IFIH1,IFIT1,IFITM1,IRF7,ISG15,MX1,OAS1,RSAD2,SIGLEC1,STAT1,ZBP1 | +  4.27  1.21E-24 | CD274,CD40,CXCL10,EIF2AK2,IFI27,IFI6,IFIH1,IFIT1,IFIT2,ISG15,MX1,OAS1,OAS2,RSAD2,SIGLEC1,STAT1,STAT2,UBE2L6,ZBP1 | +  4.57  1.39E-30 | CCL8,CD40,CD69,CD86,CXCL10,DHX58,EIF2AK2,IFI27,IFI6,IFIH1,IFIT2,IFITM1,IRF7,ISG15,MX1,OAS1,RSAD2,SIGLEC1,SOCS1,STAT1,STAT2,UBE2L6 |
| IFNA10 | +  2.20  6.16E-10 | CCL8, IFIH1 ,IFIT1, MX1, ZBP1 | +  2.59  5.69E-15 | CCL8,CXCL10,IFIH1,IFIT1,ISG15,MX1,ZBP1 | +  2.40  1.97E-09 | CXCL10,IFIH1,IFIT1,ISG15,MX1,ZBP1 | +  2.19  1.07E-07 | CCL8,CXCL10,IFIH1,ISG15,MX1 |
| IFNA14 | +  2.20  6.16E-10 | CCL8, IFIH1 ,IFIT1, MX1, ZBP1 | +  2.59  5.69E-15 | CCL8,CXCL10,IFIH1,IFIT1,ISG15,MX1,ZBP1 | +  2.40  1.97E-09 | CXCL10,IFIH1,IFIT1,ISG15,MX1,ZBP1 | +  2.19  1.07E-07 | CCL8,CXCL10,IFIH1,ISG15,MX1 |
| IFNA16 | +  2.20  9.21E-10 | CCL8, IFIH1 ,IFIT1, MX1, ZBP1 | +  2.59  1.06E-14 | CCL8,CXCL10,IFIH1,IFIT1,ISG15,MX1,ZBP1 | +  2.40  3.26E-09 | CXCL10,IFIH1,IFIT1,ISG15,MX1,ZBP1 | +  2.19  1.60E-07 | CCL8,CXCL10,IFIH1,ISG15,MX1 |

S15 Table. Continued.

|  | Bacteria infused day 16 | | Healthy day 15 | | Healthy day 16 | | Healthy day 17 | |
| --- | --- | --- | --- | --- | --- | --- | --- | --- |
| Predicted upstream regulator | +/-  z -score  *P* value | Molecules | +/-  z -score  *P* value | Molecules | +/-  z -score  *P* value | Molecules | +/-  z -score  *P* value | Molecules |
| IFNA2 | +  5.71  5.88E-48 | BST2, CCL8, CMPK2, DDX58, EIF2AK2, GBP1, GBP2, HERC5, HERC6, IFI16, IFI27, IFI44, IFI6, IFIH1, IFIT1, IFIT5, IFITM3, IRF9, MX1, MX2, OAS1, OAS2, PARP12, PARP9, PML, RSAD2, SAMD9, SP110, STAT1, TNFSF10, UBA7, USP18, XAF1, ZBP1 | +  5.90  1.73E-53 | BST2, CCL8, CXCL10,DDX58, EIF2AK2,GBP1, GBP4,HERC6, IFI16,IFI27,IFI44, IFI44L,IFI6,IFIH1, IFIT1,IFIT5,IFITM1,IRF7,IRF9,ISG15,ISG20,MX1,MX2,OAS1,PARP12, PARP9,PML, RSAD2,SAMD9, SP100,STAT1, TNFSF10,UBA7, USP18,XAF1, ZBP1 | +  7.02  9.08E-56 | ANXA1,B2M,C1S,CD274,CMPK2, CXCL10,EIF2AK2,GBP1,GBP2,GBP4,HERC5,HERC6, HSH2D,IDO1,IFI16,IFI27,IFI35,IFI44L,IFI6,IFIH1,IFIT1, IFIT2,IFIT3,IFIT5, IRF9,ISG15,ISG20,LAMP3,LGALS3BP,LY6E,MX1,MX2, OAS1,OAS2,PARP12,PARP9,PML, RSAD2,SHFL, SLC15A3,SP110, STAT1,TAP1, TDRD7,TNFSF10,TREX1,TRIM21, UBA7,UBE2L6,USP18,XAF1,ZBP1 | +  6.45  3.03E-43 | BCL2L14,CCL8,CD69,CD86,CNP,CXCL10,DDX58,EIF2AK2,GBP1,GBP4,IDO1,IFI16,IFI27,IFI35,IFI44,IFI44L,IFI6,IFIH1,IFIT2,IFIT5,IFITM1,IRF7,IRF9,ISG15,ISG20,LGALS3BP,LY6E,MX1,MX2,OAS1,PARP12,PARP9,RSAD2,SAMD9,SOCS1,STAT1,TAP1,TDRD7,TREX1,UBA7,UBE2L6,USP18,XAF1 |
| IFNA21 | +  2.20  6.16E-10 | CCL8, IFIH1, IFIT1, MX1, ZBP1 | +  2.59  5.69E-15 | CCL8, CXCL10, IFIH1, IFIT1, ISG15,MX1,ZBP1 | +  2.40  1.97E-09 | CXCL10,IFIH1, IFIT1,ISG15,MX1, ZBP1 | +  2.18  1.07E-07 | CCL8,CXCL10, IFIH1,ISG15,MX1 |
| IFNA4 | +  2.76  2.37E-12 | CCL8, GBP2, IFIH1, IFIT1, MX1, RSAD2, USP18, ZBP1 | +  3.06  1.52E-16 | CCL8, CXCL10, GBP5, IFIH1, IFIT1,ISG15,MX1,RSAD2,USP18, ZBP1 | +  3.17  8.57E-18 | CD274,CXCL10, GBP2, H2-T24, IFIH1,IFIT1,IFIT2,ISG15,MAP3K8,MX1,PMEPA1,RSAD2, USP18,ZBP1 | +  3.37  8.41E-15 | CCL8,CD69,CD86,CXCL10,GBP5, H2-T24, IFIH1,IFIT2,ISG15,MX1,RSAD2, USP18 |

S15 Table. Continued.

|  | Bacteria infused day 16 | | Healthy day 15 | | Healthy day 16 | | Healthy day 17 | |
| --- | --- | --- | --- | --- | --- | --- | --- | --- |
| Predicted upstream regulator | +/-  z -score  *P* value | Molecules | +/-  z -score  *P* value | Molecules | +/-  z -score  *P* value | Molecules | +/-  z -score  *P* value | Molecules |
| IFNA5 | +  2.20  6.16E-10 | CCL8, IFIH1, IFIT1, MX1, ZBP1 | +  2.59  5.69E-15 | CCL8,CXCL10, IFIH1,IFIT1,ISG15, MX1,ZBP1 | +  2.40  1.97E-09 | CXCL10,IFIH1, IFIT1,ISG15,MX1, ZBP1 | +  2.19  1.07E-07 | CCL8,CXCL10, IFIH1,ISG15,MX1 |
| IFNA6 | +  2.20  6.16E-10 | CCL8, IFIH1, IFIT1, MX1, ZBP1 | +  2.59  5.69E-15 | CCL8, CXCL10, IFIH1, IFIT1, ISG15, MX1, ZBP1 | +  2.40  1.97E-09 | CXCL10, IFIH1, IFIT1, ISG15, MX1, ZBP1 | +  2.19  1.07E-07 | CCL8, CXCL10, IFIH1, ISG15, MX1 |
| IFNA7 | +  2.20  6.16E-10 | CCL8, IFIH1, IFIT1, MX1, ZBP1 | +  2.59  5.69E-15 | CCL8, CXCL10, IFIH1, IFIT1, ISG15, MX1, ZBP1 | +  2.40  1.97E-09 | CXCL10, IFIH1, IFIT1, ISG15, MX1, ZBP1 | +  2.19  1.07E-07 | CCL8, CXCL10, IFIH1, ISG15, MX1 |
| IFNA8 | +  2.20  9.21E-10 | CCL8, IFIH1, IFIT1, MX1, ZBP1 | +  2.60  1.06E-14 | CCL8, CXCL10, IFIH1, IFIT1, ISG15, MX1, ZBP1 | +  2.41  3.26E-09 | CXCL10, IFIH1, IFIT1, ISG15, MX1, ZBP1 | +  2.20  1.60E-07 | CCL8, CXCL10, IFIH1, ISG15, MX1 |
| Ifnar | +  3.92  1.41E-23 | DDX58, EIF2AK2, GBP2, IFI16, IFIH1, IFITM3, IRF9, OAS1, OAS2, RNF213, RSAD2, STAT1, TNFSF10, USP18, XAF1, ZBP1 | +  4.13  4.31E-28 | CXCL10, DDX58, EIF2AK2, IFI16, IFIH1, IRF7, IRF9, ISG15, ISG20, OAS1, PNPT1, RNF213, RSAD2, STAT1, TNFSF10, USP18, XAF1, ZBP1 | +  5.28  6.08E-36 | B2M, CD274, CD40, CXCL10, EIF2AK2, GBP2, IDO1, IFI16, IFI35, IFIH1, IFIT2, IFIT3, IRF9, ISG15, ISG20, OAS1, OAS2, PNPT1, RNF213, RSAD2, STAT1, STAT2, TAP1, TNFSF10, TRIM21, UBE2L6, USP18, XAF1, ZBP1 | +  4.89  7.33E-30 | CD40, CD86, CXCL10, DDX58, EIF2AK2, IDO1, IFI16, IFI35, IFIH1, IFIT2, IRF7, IRF9, ISG15, ISG20, NLRC5, OAS1, PNPT1, RNF213, RSAD2, STAT1, STAT2, TAP1, UBE2L6, USP18, XAF1 |

S15 Table. Continued.

|  | Bacteria infused day 16 | | Healthy day 15 | | Healthy day 16 | | Healthy day 17 | |
| --- | --- | --- | --- | --- | --- | --- | --- | --- |
| Predicted upstream regulator | +/-  z -score  *P* value | Molecules | +/-  z -score  *P* value | Molecules | +/-  z -score  *P* value | Molecules | +/-  z -score  *P* value | Molecules |
| IFNAR1 | +  2.80  5.42E-20 | CMPK2, EIF2AK2, IFI16, IFI44, IFI6, IFIH1, MX2, OAS1, OAS2, PARP12, RSAD2, RTP4, STAT1, TNFSF10, USP18, XAF1 | +  2.96  8.61E-26 | CXCL10, EIF2AK2, GBP4, IFI16, IFI44, IFI6, IFIH1, IFITM1, IRF7, ISG15, MX2, OAS1, PARP12, RSAD2, RTP4, STAT1, TNFSF10, USP18, XAF1 | +  3.47  1.48E-30 | B2M, CD274, CD40, CGAS, CMPK2, CXCL10, EIF2AK2, F2, GBP4, HSH2D, IDO1, IFI16, IFI6, IFIH1, IFIT2, IFIT3, ISG15, LAMP3, MX2, OAS1, OAS2, PARP12, RSAD2, RTP4, STAT1, TNFSF10, TNFSF13B, USP18, USP25, XAF1 | +  3.55  1.68E-25 | ATF3, BCL2L14, CD40, CD86, CXCL10, EIF2AK2, GBP4, IDO1, IFI16, IFI44, IFI6, IFIH1, IFIT2, IFITM1, IRF7, ISG15, MX2, OAS1, PARP12, RSAD2, RTP4, SOCS1, STAT1, TNFSF13B, USP18, XAF1 |
| IFNAR2 | +  2.24  1.89E-20 | DDX58, HERC5, IFI44, IFI6, IFIH1, MX2, OAS1, OAS2, TNFSF10, UBA7, USP18, XAF1 | +  2.24  2.82E-25 | CXCL10, DDX58, GBP4, IFI44, IFI6, IFIH1, IFITM1, ISG15, MX2, OAS1, TNFSF10, UBA7, USP18, XAF1 | +  2.65  4.51E-25 | CXCL10, GBP4, HERC5, HSH2D, IDO1, IFI6, IFIH1, ISG15, LAMP3, MX2, OAS1, OAS2, PSME2, TNFSF10, UBA7, UBE2L6, USP18, XAF1 | +  2.45  8.39E-22 | BCL2L14, CXCL10, DDX58, GBP4, IDO1, IFI44, IFI6, IFIH1, IFITM1, ISG15, MX2, OAS1, UBA7, UBE2L6, USP18, XAF1 |

S15 Table. Continued.

|  | Bacteria infused day 16 | | Healthy day 15 | | Healthy day 16 | | Healthy day 17 | |
| --- | --- | --- | --- | --- | --- | --- | --- | --- |
| Predicted upstream regulator | +/-  z -score  *P* value | Molecules | +/-  z -score  *P* value | Molecules | +/-  z -score  *P* value | Molecules | +/-  z -score  *P* value | Molecules |
| IFNB1 | +  4.70  8.18E-31 | BST2, CMPK2, DDX58, DHX58, EIF2AK2, GBP2, HERC5, IFI16, IFI27, IFI6, IFIH1, IFIT1, IRF9, MX1, OAS1, OAS2, PARP12, PARP14, PML, RSAD2, STAT1, TNFSF10, UBA7, USP18, XAF1, ZBP1 | +  5.06  4.65E-37 | BST2, CXCL10, DDX58, DHX58, EIF2AK2, GBP4, GBP5, IFI16, IFI27, IFI6, IFIH1, IFIT1, IFITM1, IRF7, IRF9, ISG15, ISG20, MX1, OAS1, PARP12, PARP14, PML, RSAD2, STAT1, TNFSF10, UBA7, USP18, XAF1, ZBP1 | +  5.67  6.60E-32 | CASP8, CD274, CD40, CMPK2, CXCL10, EIF2AK2, GBP2, GBP4, GBP6, HERC5, IDO1, IFI16, IFI27, IFI6, IFIH1, IFIT1, IFIT2, IFIT3, IRF3, IRF9, ISG15, ISG20, MX1, OAS1, OAS2, PARP12, PARP14, PML, RNASEL, RSAD2, STAT1, STAT2, TNFSF10, TRIM21, UBA7, USP18, XAF1, ZBP1 | +  5.35  5.44E-29 | CD40, CD86, CREM, CRYAB, CXCL10, DDX58, DHX58, EIF2AK2, GBP4, GBP5, IDO1, IFI16, IFI27, IFI6, IFIH1, IFIT2, IFITM1, IRF7, IRF9, ISG15, ISG20, MCM10, MX1, NMI, NT5C3A, OAS1, PARP12, PARP14, RSAD2, SOCS1, STAT1, STAT2,UBA7, USP18,XAF1 |
| IFNE | +  2.80  1.10E-12 | BST2, HERC5, IFIH1, IFITM3, MX2, STAT1, USP18, ZBP1 | +  3.13  4.39E-19 | BST2, CXCL10, EIF4E, IFIH1, ISG15, ISG20, MX2, SLC7A2, STAT1, USP18, ZBP1 | +  3.13  2.00E-13 | CD40, CXCL10, HERC5, IFIH1, IFIT2, ISG15, ISG20, MX2, STAT1, USP18, ZBP1 | +  2.97  4.28E-12 | CD40, CD86, CXCL10, IFIH1, IFIT2, ISG15, ISG20, MX2, STAT1, USP18 |

S15 Table. Continued.

|  | Bacteria infused day 16 | | Healthy day 15 | | Healthy day 16 | | Healthy day 17 | |
| --- | --- | --- | --- | --- | --- | --- | --- | --- |
| Predicted upstream regulator | +/-  z -score  *P* value | Molecules | +/-  z -score  *P* value | Molecules | +/-  z -score  *P* value | Molecules | +/-  z -score  *P* value | Molecules |
| IFNG | +  6.13  1.93E-27 | BATF2, BST2, CCL8, CMPK2, DDX58, DKK1, DTX3L, EIF2AK2, GBP1, GBP2, HERC6, IFI16, IFI27, IFI44, IFI6, IFIH1, IFIT1, IFIT5, IFITM3, IRF9, MLKL, MX1, MX2, OAS1, OAS2,PARP14, PARP9,PLAAT3, PML,PSMF1, PTX3,RSAD2, RTP4, SAMD9,SP110, STAT1,TNFSF10, USP18,XAF1 | +  6.16  4.54E-29 | BST2, C4A/C4B, CCL8,CXCL10,DDX58,DKK1,DTX3L,EIF2AK2,GBP1, GBP4,GBP5, HERC6,IFI16, IFI27,IFI44,IFI44L, IFI6,IFIH1,IFIT1, IFIT5,IFITM1,IRF7,IRF9,ISG15,ISG20,MX1,MX2,OAS1, PARP14,PARP9, PML,RSAD2,RTP4,SAMD9,SP100, STAT1,TNFSF10,USP18,XAF1 | +  7.69  3.22E-46 | AGRN,B2M,BATF2,C1QB,C1QC,C2,C4A/C4B,CASP4,CASP8,CCL11,CCND1,CD274,CD40,CGAS,CMPK2,CXCL10,DKK1,DTX3L,EDNRB,EIF2AK2,ESM1,FCGR1A,GBP1,GBP2,GBP4,GBP6,HERC6,IDO1,IFI16,IFI27,IFI35,IFI44L,IFI6,IFIH1,IFIT1,IFIT2,IFIT3,IFIT5,IL23A,IRF3,IRF4,IRF9,ISG15,ISG20,KRT17,KYNU,LAMP3,LGALS3BP,LGALS9,LY6E,MAP3K8,MLKL,MST1R,MX1,MX2,NAMPT,OAS1,OAS2,OPTN,P2RY14,PARP14,PARP9,PLAUR,PML,PSMA2,PSME2,PSMF1,RNF114,RSAD2,RTP4,SCLY,SLC15A3,SLC40A1,SOAT1,SP110,STAT1,STAT2,SYT7,TAP1,TNFSF10,TNFSF13B,TREM2,TRIM21,UBE2L6, USP18,XAF1 | +  7.42  8.60E-33 | ABLIM3,ALOX12, ALOX5AP,ARG2, ATF3,C1QB,C1QC,C2,CALHM6, CASP4,CCL8, CD40,CD86,CFB, CREM,CXCL10, DDX58,DKK1, EIF2AK2,FBP1, FCGR1A,GBP1, GBP4,GBP5,IDO1, IFI16,IFI27,IFI35, IFI44,IFI44L,IFI6, IFIH1,IFIT2,IFIT5, IFITM1,IRF7,IRF9, ISG15,ISG20, KRT17,LGALS3BP,LGALS9,LY6E, MLKL,MST1R,MX1,MX2,NCF2,NLRC5,NMI,OAS1, PARP14,PARP9, PIGR,PSMF1, PTX3,RSAD2, RTP4,SAMD9, SCNN1A,SLC12A2,SOCS1,STAT1,STAT2,TAP1,TNFSF13B,UBE2L6,USP18,WARS1,XAF1 |

S15 Table. Continued.

|  | Bacteria infused day 16 | | Healthy day 15 | | Healthy day 16 | | Healthy day 17 | |
| --- | --- | --- | --- | --- | --- | --- | --- | --- |
| Predicted upstream regulator | +/-  z -score  *P* value | Molecules | +/-  z -score  *P* value | Molecules | +/-  z -score  *P* value | Molecules | +/-  z -score  *P* value | Molecules |
| IFNK | +  2.45  9.20E-11 | EIF2AK2,IFIH1,MX1,OAS1,STAT1,ZBP1 | +  2.45  3.98E-13 | CXCL10,EIF2AK2,IFIH1,MX1,OAS1,STAT1,ZBP1 | +  2.65  2.59E-11 | CD40,CXCL10,EIF2AK2,IFIH1,MX1,OAS1,STAT1,ZBP1 | +  2.65  1.69E-11 | CD40,CD86,CXCL10,EIF2AK2,IFIH1,MX1,OAS1,STAT1 |
| IFNL1 | +  5.08  3.49E-48 | BST2,CMPK2,DDX58,EIF2AK2,GBP1,HERC5,HERC6,IFI27,IFI44,IFI6,IFIH1,IFIT1,IFIT5,IFITM3,IRF9,MLKL,MX1,OAS1,OAS2,PML,RSAD2,RTP4,SAMD9,SP110,STAT1,USP18,XAF1 | +  5.19  1.21E-51 | BST2,CXCL10,DDX58,EIF2AK2,GBP1,GBP5,HERC6,IFI27,IFI44,IFI44L,IFI6,IFIH1,IFIT1,IFIT5,IFITM1,IRF9,ISG15,ISG20,MX1,OAS1,PML,RSAD2,RTP4,SAMD9,SP100,STAT1,USP18,XAF1 | +  6.04  1.24E-54 | CD40,CMPK2,CXCL10,EIF2AK2,GBP1,HERC5,HERC6,IFI27,IFI35,IFI44L,IFI6,IFIH1,IFIT1,IFIT2,IFIT3,IFIT5,IRF9,ISG15,ISG20,LAMP3,LGALS3BP,MLKL,MX1,OAS1,OAS2,PML,RSAD2,RTP4,SHFL,SLC15A3,SP110,STAT1,STAT2,TDRD7,TMEM140,UBE2L6,USP18,XAF1 | +  5.71  1.16E-47 | ATF3,CCR7,CD40,CXCL10,DDX58,EIF2AK2,GBP1,GBP5,IFI27,IFI35,IFI44,IFI44L,IFI6,IFIH1,IFIT2,IFIT5,IFITM1,IRF9,ISG15,ISG20,LGALS3BP,MLKL,MX1,OAS1,RSAD2,RTP4,SAMD9,STAT1,STAT2,TDRD7,TMEM140,UBE2L6,USP18,XAF1 |
| IFNL3 | +  2.42  6.57E-10 | DDX58,IFIH1,MX1,RSAD2,STAT1,USP18 | +  2.60  4.19E-12 | DDX58,IFIH1,ISG20,MX1,RSAD2,STAT1,USP18 | +  2.76  3.90E-10 | IFIH1,IL23A,ISG20,MX1,RNASEL,RSAD2,STAT1,USP18 | +  2.00  8.17E-06 | CXCL10,IRF7,MX1,RSAD2 |
| IFNL4 | +  2.80  4.08E-18 | DDX58,DHX58,IFIH1,IFIT1,MX1,OAS1,OAS2,STAT1 | +  2.97  2.66E-21 | CXCL10,DDX58,DHX58,IFIH1,IFIT1,ISG15,MX1,OAS1,STAT1 | +  2.80  2.86E-14 | CXCL10,IFIH1,IFIT1,ISG15,MX1,OAS1,OAS2,STAT1 | +  2.79  2.55E-10 | DDX58,IFIH1,ISG20,MX1,RSAD2,SOCS1,STAT1,USP18 |
| IKBKG | +  2.00  1.07E-03 | GBP2,IFI16,PTX3,TNFSF10 | +  2.20  6.20E-05 | CXCL10,IFI16,IRF7,ISG15,TNFSF10 | +  2.21  1.05E-02 | CXCL10,GBP2,IFI16,ISG15,TNFSF10 | +  2.80  1.85E-14 | CXCL10,DDX58,DHX58,IFIH1,ISG15,MX1,OAS1,STAT1 |

S15 Table. Continued.

|  | Bacteria infused day 16 | | Healthy day 15 | | Healthy day 16 | | | Healthy day 17 | |
| --- | --- | --- | --- | --- | --- | --- | --- | --- | --- |
| Predicted upstream regulator | +/-  z -score  *P* value | Molecules | +/-  z -score  *P* value | Molecules | +/-  z -score  *P* value | Molecules | | +/-  z -score  *P* value | Molecules |
| IKZF1 | -  -2.75  5.43E-10 | CYP2J2,DKK1,DTX3L,EPSTI1,IFI16,IFI27,IFI6,IFIT5,PAX5,RNF213,RTP4 | -  -2.95  6.70E-08 | DKK1,DTX3L,EPSTI1,IFI16,IFI27,IFI6,IFIT5,RNF213,RTP4 | -  -3.09  9.75E-07 | B2M,DIPK1A,DKK1,DTX3L,EPSTI1,IFI16,IFI27,IFI6,IFIT3,IFIT5,IRF4,RNF213,RTP4 | -  -2.61  8.16E-03 | | DKK1,IFI16,IFI27,IFI6,IFIT5,RNF213,RTP4 |
| IKZF3 | -  -2.65  3.20E-09 | DDX58,DKK1,IFI27,IFI6,IFIT5,RNF213,RTP4 | -  -2.83  4.19E-11 | DDX58,DKK1,IFI27,IFI6,IFIT5,IRF7,RNF213,RTP4 | -  -2.63  5.13E-06 | DKK1,IFI27,IFI6,IFIT3,IFIT5,RNF213,RTP4 | | -  -2.83  2.52E-07 | DDX58,DKK1,IFI27,IFI6,IFIT5,IRF7,RNF213,RTP4 |
| IL10RA | -  -3.00  7.59E-07 | BATF2,GBP2,IFI16,MLKL,PLAAT3,RNF213,RSAD2,STAT1,ZBP1 | -  -2.65  4.36E-05 | GBP5,IFI16,IRF7,RNF213,RSAD2,STAT1,ZBP1 | -  -3.76  1.30E-09 | BATF2,CD40,CLEC12A,DRAM1,EDNRB,GBP2,GBP6,GDA,IFI16,IL23A,MLKL,NAMPT,RIPK3,RNF213,RSAD2,STAT1,TAP1,ZBP1 | | -  -4.12  3.98E-09 | ARG2,CALHM6,CCR7,CD40,CD69,CFB,COL14A1,DRAM1,GBP5,IFI16,IRF7,MLKL,NLRC5,RNF213,RSAD2,STAT1,TAP1 |
| IL1B | +  3.20  6.99E-08 | CCL8,CMPK2,GBP1,GBP2,HERC5,IFI16,IFIT1,LBP,MEF2B,MX1,OAS2,PTX3,RSAD2,STAT1,TNFSF10,USP18 | +  3.32  1.00E-06 | CCL8,CXCL10,EIF4E,GBP1,IFI16,IFIT1,IRF7,ISG15,ISG20,MX1,RSAD2,STAT1,TNFSF10,USP18 | +  5.39  5.10E-13 | ANXA1,B2M,CASP4,CCL11,CD274,CD40,CMPK2,CXCL10,GBP1,GBP2,GBP6,HERC5,IDO1,IFI16,IFIT1,IFIT3,IL23A,IRF4,ISG15,ISG20,LGALS9,MAP3K8,MAPT,MX1,NAMPT,OAS2,OXTR,PSME2,RSAD2,SCLY,STAT1,TCIM,TNFSF10,TNFSF13B,TREM2,UBE2L6,USP18 | | +  4.66  1.10E-11 | ATF3,CASP4,CCL8,CCR7,CD40,CD69,CD86,CFB,CREM,CRYAB,CXCL10,EPAS1,GBP1,H19,IDO1,IFI16,IRF7,ISG15,ISG20,LGALS9,MX1,NMI,OSMR,OXTR,PIGR,PTX3,RGS16,RSAD2,SCNN1A,SOCS1,STAT1,TNFSF13B,UBE2L6,USP18 |

S15 Table. Continued.

|  | Bacteria infused day 16 | | Healthy day 15 | | Healthy day 16 | | Healthy day 17 | |
| --- | --- | --- | --- | --- | --- | --- | --- | --- |
| Predicted upstream regulator | +/-  z -score  *P* value | Molecules | +/-  z -score  *P* value | Molecules | +/-  z -score  *P* value | Molecules | +/-  z -score  *P* value | Molecules |
| IL1RN | -  -4.36  9.22E-27 | DDX58,GBP1,HERC6,IFI27,IFI44,IFI6,IFIH1,IFIT5,IRF9,MX1,MX2,OAS1,OAS2,PML,RSAD2,RTP4,SAMD9,TNFSF10,USP18 | -  -4.69  2.77E-33 | DDX58,GBP1,HERC6,IFI27,IFI44,IFI44L,IFI6,IFIH1,IFIT5,IRF7,IRF9,ISG20,MX1,MX2,OAS1,PML,RSAD2,RTP4,SAMD9,SP100,TNFSF10,USP18 | -  -4.80  3.15E-24 | GBP1,HERC6,IFI27,IFI44L,IFI6,IFIH1,IFIT3,IFIT5,IL23A,IRF9,ISG20,LAMP3,LGALS9,MX1,MX2,OAS1,OAS2,PML,RSAD2,RTP4,SLC15A3,STAT2,TNFSF10,USP18 | -  -4.58  1.52E-20 | ATF3,DDX58,GBP1,IFI27,IFI44,IFI44L,IFI6,IFIH1,IFIT5,IRF7,IRF9,ISG20,LGALS9,MX1,MX2,OAS1,RSAD2,RTP4,SAMD9,STAT2,USP18 |
| IL21 | +  2.33  5.97E-09 | CMPK2,EIF2AK2,HERC6,IFI16,IFIT1,OAS2,PAX5,RSAD2,USP18 | +  2.53  1.39E-10 | CXCL10,EIF2AK2,GBP5,HERC6,IFI16,IFIT1,IRF7,ISG15,RSAD2,USP18 | +  2.93  5.12E-16 | CCL11,CMPK2,CXCL10,EIF2AK2,GBP6,HERC6,HSH2D,IDO1,IFI16,IFIT1,IFIT2,IFIT3,IL23A,IRF4,ISG15,OAS2,RSAD2,STAT2,TAP1,USP18 | +  3.29  3.94E-14 | ARG2,CALHM6,CCR7,CD69,CD86,CXCL10,EIF2AK2,GBP5,IDO1,IFI16,IFIT2,IRF7,ISG15,RSAD2,SOCS1,STAT2,TAP1,USP18 |
| IL27 | +  2.61  4.81E-07 | BST2,EIF2AK2,GBP2,MX1,OAS1,STAT1,TNFSF10 | +  2.61  2.94E-07 | BST2,CXCL10,EIF2AK2,MX1,OAS1,STAT1,TNFSF10 | +  3.41  1.65E-08 | B2M,CD274,CXCL10,EIF2AK2,GBP2,MX1,OAS1,STAT1,STAT2,TAP1,TNFSF10,TNFSF13B | +  3.11  9.41E-08 | CD69,CD86,CXCL10,EIF2AK2,MX1,OAS1,SOCS1,STAT1,STAT2,TAP1,TNFSF13B |

S15 Table. Continued.

|  | Bacteria infused day 16 | | Healthy day 15 | | Healthy day 16 | | Healthy day 17 | |
| --- | --- | --- | --- | --- | --- | --- | --- | --- |
| Predicted upstream regulator | +/-  z -score  *P* value | Molecules | +/-  z -score  *P* value | Molecules | +/-  z -score  *P* value | Molecules | +/-  z -score  *P* value | Molecules |
| Interferon alpha | +  5.05  1.09E- 41 | BST2,DDX58,DHX58,EIF2AK2,EPSTI1,GBP1,GBP2,HERC5,HERC6,IFI16,IFI27,IFI44,IFI6,IFIH1,IFIT1,IFITM3,IRF9,MX1,MX2,OAS1,OAS2,PARP12,PARP14,PARP9,PML,RNF213,RSAD2,RTP4,SAMD9,SIGLEC1,SP110,STAT1,TNFSF10,UBA7,USP18,ZBP1 | +  5.51  2.38E-54 | ADAR,BST2,CXCL10,DDX58,DHX58,EIF2AK2,EPSTI1,GBP1,GBP5,HERC6,IFI16,IFI27,IFI44,IFI44L,IFI6,IFIH1,IFIT1,IFITM1,IRF7,IRF9,ISG15,ISG20,MX1,MX2,OAS1,PARP12,PARP14,PARP9,PML,PNPT1,RNF213,RSAD2,RTP4,SAMD9,SIGLEC1,SP100,STAT1,TNFSF10,TRANK1,UBA7,USP18,ZBP1 | +  6.57  1.62E-59 | ADAR,B2M,C3AR1,CASP8,CCND1,CD274,CD40,CGAS,CMTR1,COX7A1,CXCL10,EIF2AK2,EPSTI1,FCGR1A,GBP1,GBP2,GBP6,HERC5,HERC6,IDO1,IFI16,IFI27,IFI35,IFI44L,IFI6,IFIH1,IFIT1,IFIT2,IFIT3,IRF4,IRF9,ISG15,ISG20,LAMP3,LGALS9,MX1,MX2,OAS1,OAS2,PARP10,PARP12,PARP14,PARP9,PML,PNPT1,RNASEL,RNF213,RSAD2,RTP4,SHFL,SIGLEC1,SP110,STAT1,STAT2,TAP1,TDRD7,TMEM140,TNFSF10,TNFSF13B,TRANK1,TRIM21,UBA7,UBE2L6,USP18,USP25,ZBP1 | +  6.28  3.71E-46 | ADAR,ATF3,CCR7,CD40,CD69,CD86,CXCL10,DDX58,DHX58,EIF2AK2,FCGR1A,GBP1,GBP5,IDO1,IFI16,IFI27,IFI35,IFI44,IFI44L,IFI6,IFIH1,IFIT2,IFITM1,IRF7,IRF9,ISG15,ISG20,LGALS9,MX1,MX2,NMI,NT5C3A,OAS1,PARP12,PARP14,PARP9,PNPT1,RNF213,RSAD2,RTP4,SAMD9,SIGLEC1,SLC5A1,SOCS1,STAT1,STAT2,TAP1,TDRD7,TMEM140,TNFSF13B,TRANK1,UBA7,UBE2L6,USP18,WARS1 |

S15 Table. Continued.

|  | Bacteria infused day 16 | | Healthy day 15 | | Healthy day 16 | | Healthy day 17 | |
| --- | --- | --- | --- | --- | --- | --- | --- | --- |
| Predicted upstream regulator | +/-  z -score  *P* value | Molecules | +/-  z -score  *P* value | Molecules | +/-  z -score  *P* value | Molecules | +/-  z -score  *P* value | Molecules |
| IRF1 | +  4.50  9.49E-29 | CMPK2,DDX58,EIF2AK2,GBP2,IFI27,IFI44,IFI6,IFIH1,IFIT1,IFIT5,IFITM3,IRF9,MX1,OAS1,OAS2,PLAAT3,PML,RSAD2,SP110,STAT1,TNFSF10,XAF1 | +  4.48  9.54E-28 | CXCL10,DDX58,EIF2AK2,IFI27,IFI44,IFI44L,IFI6,IFIH1,IFIT1,IFIT5,IFITM1,IRF7,IRF9,ISG15,MX1,OAS1,PML,RSAD2,STAT1,TNFSF10,XAF1 | +  5.47  1.31E-36 | B2M,CASP8,CCND1,CD274,CD40,CMPK2,CXCL10,EIF2AK2,GBP2,IDO1,IFI27,IFI35,IFI44L,IFI6,IFIH1,IFIT1,IFIT2,IFIT3,IFIT5,IRF4,IRF9,ISG15,MX1,OAS1,OAS2,PML,PSME2,RSAD2,SP110,STAT1,STAT2,TAP1,TNFSF10,TNFSF13B,TRIM21,XAF1 | +  4.53  4.58E-26 | CD40,CFB,CXCL10,DDX58,EIF2AK2,IDO1,IFI27,IFI35,IFI44,IFI44L,IFI6,IFIH1,IFIT2,IFIT5,IFITM1,IRF7,IRF9,ISG15,MX1,OAS1,PIGR,RSAD2,SOCS1,STAT1,STAT2,TAP1,TNFSF13B,XAF1 |
| IRF3 | +  4.67  9.84E-30 | CMPK2,DDX58,DHX58,EIF2AK2,GBP1,IFI16,IFI27,IFI44,IFI6,IFIH1,IFIT1,IFITM3,OAS1,OAS2,PARP12,PARP14,PLAC8,PML,RSAD2,STAT1,TNFSF10,USP18,ZBP1 | +  5.03  4.21E-38 | ADAR,CXCL10,DDX58,DHX58,EIF2AK2,GBP1,GBP5,IFI16,IFI27,IFI44,IFI44L,IFI6,IFIH1,IFIT1,IRF7,ISG15,ISG20,OAS1,PARP12,PARP14,PLAC8,PML,RSAD2,STAT1,TNFSF10,USP18,ZBP1 | +  5.73  7.33E-37 | ADAR,B2M,CD274,CD40,CMPK2,CXCL10,EIF2AK2,FCGR1A,GBP1,IFI16,IFI27,IFI44L,IFI6,IFIH1,IFIT1,IFIT2,IFIT3,IL23A,IRF3,ISG15,ISG20,OAS1,OAS2,PARP12,PARP14,PLAC8,PML,RSAD2,STAT1,STAT2,TAP1,TDRD7,TNFSF10,TREX1,UBE2L6,USP18,ZBP1 | +  5.89  2.92E-39 | ADAR,ARG2,CALHM6,CD40,CD58,CD69,CD86,CXCL10,DDX58,DHX58,EIF2AK2,FCGR1A,GBP1,GBP5,IFI16,IFI27,IFI44,IFI44L,IFI6,IFIH1,IFIT2,IRF7,ISG15,ISG20,NLRC5,NT5C3A,OAS1,PARP12,PARP14,PLAC8,RSAD2,STAT1,STAT2,TAP1,TDRD7,TREX1,UBE2L6,USP18 |

S15 Table. Continued.

|  | Bacteria infused day 16 | | Healthy day 15 | | Healthy day 16 | | Healthy day 17 | |
| --- | --- | --- | --- | --- | --- | --- | --- | --- |
| Predicted upstream regulator | +/-  z -score  *P* value | Molecules | +/-  z -score  *P* value | Molecules | +/-  z -score  *P* value | Molecules | +/-  z -score  *P* value | Molecules |
| IRF4 | -  -2.62  2.53E-06 | GBP1,IRF9,OAS1,PAX5,SPIB,STAT1,TNFSF10 | -  -2.42  5.07E-09 | CXCL10,GBP1,IRF7,IRF9,ISG15,ISG20,OAS1,STAT1,TNFSF10 | -  -3.10  3.60E-10 | B2M,CXCL10,GBP1,IRF4,IRF9,ISG15,ISG20,OAS1,PLAUR,PSMA2,STAT1,STAT2,TNFSF10,TNFSF13B,TRIM21 | -  -2.63  8.18E-06 | CXCL10,GBP1,IRF7,IRF9,ISG15,ISG20,OAS1,STAT1,STAT2,TNFSF13B |
| IRF5 | +  3.53  2.04E-20 | CMPK2,DDX58,DHX58,IFI44,IFIH1,IFIT1,IFITM3,OAS1,OAS2,PARP12,RSAD2,SP110,STAT1,TNFSF10 | +  3.52  6.89E-21 | CXCL10,DDX58,DHX58,IFI44,IFIH1,IFIT1,IRF7,ISG15,ISG20,OAS1,PARP12,RSAD2,STAT1,TNFSF10 | +  4.14  8.85E-21 | CMPK2,CXCL10,IFIH1,IFIT1,IFIT2,IFIT3,IL23A,ISG15,ISG20,NAMPT,OAS1,OAS2,PARP12,RSAD2,SP110,STAT1,STAT2,TNFSF10,UBE2L6 | +  3.91  8.81E-17 | CXCL10,DDX58,DHX58,IFI44,IFIH1,IFIT2,IRF7,ISG15,ISG20,NT5C3A,OAS1,PARP12,RSAD2,STAT1,STAT2,UBE2L6 |
| IRF7 | +  5.16  4.75E-41 | CCL8,CMPK2,DDX58,DHX58,GBP1,HERC5,IFI16,IFI44,IFI6,IFIH1,IFIT1,IFITM3,IRF9,MX1,MX2,OAS1,OAS2,PARP12,PARP14,PLAC8,RSAD2,RTP4,STAT1,TNFSF10,UBA7,USP18,XAF1,ZBP1 | +  5.59  8.71E-53 | ADAR,CCL8,CXCL10,DDX58,DHX58,GBP1,GBP4,GBP5,IFI16,IFI44,IFI44L,IFI6,IFIH1,IFIT1,IFITM1,IRF7,IRF9,ISG15,ISG20,MX1,MX2,OAS1,PARP12,PARP14,PLAC8,RSAD2,RTP4,STAT1,TNFSF10,UBA7,USP18,XAF1,ZBP1 | +  6.60  5.54E-56 | ADAR,CASP4,CD40,CMPK2,CXCL10,FCGR1A,GBP1,GBP4,HERC5,IDO1,IFI16,IFI35,IFI44L,IFI6,IFIH1,IFIT1,IFIT2,IFIT3,IRF9,ISG15,ISG20,MAP3K8,MX1,MX2,NAMPT,OAS1,OAS2,PARP12,PARP14,PLAC8,PSME2,RSAD2,RTP4,STAT1,STAT2,TAP1,TDRD7,TNFSF10,TNFSF13B,TREX1,TRIM21,UBA7,UBE2L6,USP18,USP25,XAF1,ZBP1 | +  6.68  3.41E-57 | ADAR,CALHM6,CASP4,CCL8,CD40,CD69,CXCL10,DDX58,DHX58,FCGR1A,GBP1,GBP4,GBP5,IDO1,IFI16,IFI35,IFI44,IFI44L,IFI6,IFIH1,IFIT2,IFITM1,IRF7,IRF9,ISG15,ISG20,MX1,MX2,NMI,NT5C3A,OAS1,PARP12,PARP14,PLAC8,RSAD2,RTP4,SOCS1,STAT1,STAT2,TAP1,TDRD7,TNFSF13B,TREX1,UBA7,UBE2L6,USP18,XAF1 |

S15 Table. Continued.

|  | Bacteria infused day 16 | | Healthy day 15 | | Healthy day 16 | | Healthy day 17 | |
| --- | --- | --- | --- | --- | --- | --- | --- | --- |
| Predicted upstream regulator | +/-  z -score  *P* value | Molecules | +/-  z -score  *P* value | Molecules | +/-  z -score  *P* value | Molecules | +/-  z -score  *P* value | Molecules |
| Irgm1 | -  -2.00  1.38E-05 | IFI16,OAS2,RSAD2,USP18 | -  -2.24  2.43E-07 | CXCL10,IFI16,IRF7,RSAD2,USP18 | -  -2.65  2.16E-07 | CXCL10,IFI16,IFIT2,IFIT3,OAS2,RSAD2,USP18 | -  -2.45  3.07E-06 | CXCL10,IFI16,IFIT2,IRF7,RSAD2,USP18 |
| JAK | +  2.83  1.10E-12 | DDX58,EIF2AK2,IFI6,IFIH1,IFIT1,IFITM3,RSAD2,STAT1 | +  3.00  6.34E-15 | CXCL10,DDX58,EIF2AK2,IFI6,IFIH1,IFIT1,ISG15,RSAD2,STAT1 | +  3.16  2.00E-13 | CD40,CXCL10,EIF2AK2,IFI6,IFIH1,IFIT1,IFIT2,IFIT3,ISG15,RSAD2,STAT1 | +  3.00  1.11E-13 | CD40,CXCL10,DDX58,EIF2AK2,IFI6,IFIH1,IFIT2,ISG15,RSAD2,SOCS1,STAT1 |
| JAK1 | +  2.24  3.23E-06 | EIF2AK2,IRF9,MX1,STAT1,USP18 | +  2.24  6.89E-08 | EIF2AK2,IRF7,IRF9,MX1,STAT1,USP18 | +  2.99  1.21E-09 | CD40,EIF2AK2,IFIT2,IRF9,MAP3K8,MX1,STAT1,STAT2,TAP1,USP18 | +  2.99  3.38E-11 | CD40,EIF2AK2,IFIT2,IRF7,IRF9,MX1,OSMR,STAT1,STAT2,TAP1,USP18 |
| JAK1/2 | +  2.22  3.47E-06 | EIF2AK2,GBP2,MX1,PLAC8,RSAD2 | +  2.63  1.92E-09 | EIF2AK2,GBP5,IRF7,ISG15,MX1,PLAC8,RSAD2 | +  2.63  5.13E-06 | EIF2AK2,GBP2,GDA,ISG15,MX1,PLAC8,RSAD2 | +  2.81  2.52E-07 | CD69,EIF2AK2,GBP5,IRF7,ISG15,MX1,PLAC8,RSAD2 |
| KRAS | -  -3.15  7.65E-06 | CPM,CYP2J2,EIF2AK2,IFI6,IFIT1,IFITM3,IRF9,MX1,MX2,OAS1,STAT1,TNFSF10 | -  -3.30  1.72E-09 | ADAR,CXCL10,EIF2AK2,EIF4E,IFI6,IFIT1,IFITM1,IRF9,ISG15,MX1,MX2,OAS1,SP100,STAT1,TNFSF10 | -  -2.86  5.59E-10 | ADAR,AGRN,B2M,BTC,BZW2,CCND1,CD274,CXCL10,EIF2AK2,IDO1,IFI6,IFIT1,IRF9,ISG15,LAMP3,MX1,MX2,OAS1,P2RY14,PGAM2,SLC34A2,SOAT1,STAT1,STAT2,TAP1,TNFSF10,XRN2 | -  -3.05  1.31E-06 | ADAR,ATF3,BCL2L14,BZW2,CRYAB,CXCL10,EIF2AK2,H1-2,IDO1,IFI6,IFITM1,IRF9,ISG15,MX1,MX2,NCF2,OAS1,PYCARD,STAT1,STAT2,TAP1 |

S15 Table. Continued.

|  | Bacteria infused day 16 | | Healthy day 15 | | Healthy day 16 | | Healthy day 17 | |
| --- | --- | --- | --- | --- | --- | --- | --- | --- |
| Predicted upstream regulator | +/-  z -score  *P* value | Molecules | +/-  z -score  *P* value | Molecules | +/-  z -score  *P* value | Molecules | +/-  z -score  *P* value | Molecules |
| MAPK1 | -  -4.77  1.18E-24 | BST2,DDX58,EIF2AK2,GBP1,GBP2,HERC5,IFI16,IFI27,IFI44,IFI6,IFIH1,IFIT1,IFIT5,IFITM3,IRF9,MX2,OAS1,OAS2,PARP12,PML,SP110,STAT1,TNFSF10,USP18 | -  -5.07  3.11E-30 | ADAR,BST2,DDX58,EIF2AK2,GBP1,GBP5,IFI16,IFI27,IFI44,IFI6,IFIH1,IFIT1,IFIT5,IFITM1,IRF7,IRF9,ISG15,ISG20,MX2,OAS1,PARP12,PML,SP100,STAT1,TNFSF10,TRANK1,USP18 | -  -6.28  6.80E-31 | ADAR,C1S,CCND1,EIF2AK2,GBP1,GBP2,GRAMD1B,HERC5,IFI16,IFI27,IFI35,IFI6,IFIH1,IFIT1,IFIT2,IFIT3,IFIT5,IRF9,ISG15,ISG20,KRT17,LAMP3,LGALS3BP,MX2,OAS1,OAS2,PARP12,PML,PSME2,SP110,STAT1,STAT2,TAP1,TDRD7,TNFSF10,TRANK1,TRIM21,TRIM25,TRIM34,UBE2L6,USP18 | -  -4.58  2.59E-28 | ADAR,ARG2,ATF3,CD69,CFB,DDX58,EIF2AK2,GBP1,GBP5,IFI16,IFI27,IFI35,IFI44,IFI6,IFIH1,IFIT2,IFIT5,IFITM1,IRF7,IRF9,ISG15,ISG20,KRT17,LGALS3BP,MX2,NMI,OAS1,PARP12,RGS16,SCNN1A,STAT1,STAT2,TAP1,TDRD7,TRANK1,TRIM34,UBE2L6,USP18 |
| MAVS | +  3.27  3.57E-16 | CMPK2,DDX58,DHX58,IFIT1,IFITM3,OAS1,OAS2,PARP12,RSAD2,STAT1,USP18 | +  3.39  2.16E-20 | ADAR,CXCL10,DDX58,DHX58,IFIT1,IRF7,ISG15,ISG20,OAS1,PARP12,RSAD2,STAT1,USP18 | +  3.92  1.17E-19 | ADAR,CGAS,CMPK2,CXCL10,IFIT1,IFIT2,IFIT3,ISG15,ISG20,OAS1,OAS2,PARP12,RSAD2,STAT1,STAT2,UBE2L6,USP18 | +  3.91  4.63E-20 | ADAR,CXCL10,DDX58,DHX58,IFIT2,IRF7,ISG15,ISG20,NT5C3A,OAS1,PARP12,RSAD2,SOCS1,STAT1,STAT2,UBE2L6,USP18 |
| mir-21 | -  -2.60  3.99E-05 | DHX58,GBP2,IFI16,OAS2,SIGLEC1,STAT1,UBA7 | -  -2.78  2.37E-06 | CXCL10,DHX58,GBP5,IFI16,PPA1,SIGLEC1,STAT1,UBA7 | -  -4.30  4.01E-11 | CASP4,CCND1,CD274,CXCL10,FCGR1A,GBP2,GBP6,IDO1,IFI16,OAS2,PPA1,PSME2,SIGLEC1,STAT1,STAT2,TAP1,TREM2,UBA7,UBE2L6 | -  -3.83  5.58E-08 | CASP4,CXCL10,DHX58,FCGR1A,GBP5,IDO1,IFI16,NLRC5,PPA1,SIGLEC1,STAT1,STAT2,TAP1,UBA7,UBE2L6 |
| MSC | +  2.45  3.03E-08 | EPSTI1,IFI27,IFI44,IFIT1,PAX5,XAF1 | +  2.65  3.96E-10 | EPSTI1,IFI27,IFI44,IFI44L,IFIT1,IRF7,XAF1 | +  2.24  2.10E-04 | EPSTI1,IFI27,IFI44L,IFIT1,XAF1 | +  2.24  1.64E-04 | IFI27,IFI44,IFI44L,IRF7,XAF1 |

S15 Table. Continued.

|  | Bacteria infused day 16 | | Healthy day 15 | | Healthy day 16 | | Healthy day 17 | |
| --- | --- | --- | --- | --- | --- | --- | --- | --- |
| Predicted upstream regulator | +/-  z -score  *P* value | Molecules | +/-  z -score  *P* value | Molecules | +/-  z -score  *P* value | Molecules | +/-  z -score  *P* value | Molecules |
| NFATC2 | +  2.00  4.72E-03 | CMPK2,PML,RSAD2,STAT1 | +  2.65  2.15E-07 | CXCL10,GBP4,IRF7,ISG15,ISG20,PML,RSAD2,STAT1 | +  2.70  1.72E-11 | CASP4,CD274,CD40,CMPK2,CXCL10,GBP4,IFIT2,IFIT3,IRF4,ISG15,ISG20,PML,RSAD2,STAT1,STAT2,TNFSF13B,USP25 | +  2.50  7.29E-12 | CASP4,CCR7,CD40,CD86,CRYAB,CXCL10,GBP4,IFIT2,IRF7,ISG15,ISG20,NMI,RSAD2,SOCS1,STAT1,STAT2,TNFSF13B |
| NFkB (complex) | +  2.40  5.00E-03 | CCL8,GBP2,HERC5,PTX3,RSAD2,SPIB,TNFSF10 | +  2.16  1.35E-02 | CCL8,CXCL10,IRF7,ISG15,RSAD2,TNFSF10 | +  3.92  3.21E-09 | CASP4,CASP8,CCL11,CCND1,CD274,CD40,CXCL10,EDNRB,FCGR1A,GBP2,HERC5,IDO1,IL23A,IRF3,IRF4,ISG15,KRT17,KYNU,MAP3K8,NAMPT,RSAD2,SCLY,SOAT1,TAP1,TNFSF10 | +  4.01  2.41E-08 | ATF3,CASP4,CCL8,CCR7,CD40,CD69,CD86,CFB,CREM,CXCL10,EPAS1,FCGR1A,IDO1,IRF7,ISG15,KRT17,MSTN,NCF2,PTX3,RGS16,RSAD2,SOCS1,TAP1 |
| NGLY1 | -  -2.39  1.43E-09 | IFI27,IFI44,IFIT1,OAS1,RSAD2,USP18 | -  -2.76  1.01E-13 | CXCL10,IFI27,IFI44,IFI44L,IFIT1,OAS1,RSAD2,USP18 | -  -2.91  3.12E-11 | CXCL10,IFI27,IFI44L,IFIT1,IFIT2,IFIT3,OAS1,RSAD2,USP18 | -  -2.76  7.37E-10 | CXCL10,IFI27,IFI44,IFI44L,IFIT2,OAS1,RSAD2,USP18 |
| NKX2-3 | -  -4.36  3.22E-22 | BATF2,CMPK2,DDX58,DHX58,EIF2AK2,GBP1,GBP2,PARP12,PARP14,PARP9,RNF213,RTP4,SAMD9,SP110,STAT1,UBA7,USP18,XAF1,ZNFX1 | -  -4.00  4.04E-18 | DDX58,DHX58,EIF2AK2,GBP1,PARP12,PARP14,PARP9,PNPT1,RNF213,RTP4,SAMD9,STAT1,UBA7,USP18,XAF1,ZNFX1 | -  -4.75  6.23E-26 | BATF2,CMPK2,EIF2AK2,GBP1,GBP2,IDO1,LY6E,MFNG,PARP10,PARP12,PARP14,PARP9,PNPT1,PTPRE,RNF213,RTP4,SHFL,SLC40A1,SP110,STAT1,STAT2,TAP1,TCIM,TIPARP,TRIM21,UBA7,UBE2L6,USP18,XAF1,ZNFX1 | -  -4.60  2.39E-20 | C2CD4B,CRYAB,DDX58,DHX58,EIF2AK2,GBP1,IDO1,LY6E,NT5C3A,PARP12,PARP14,PARP9,PLEKHA4,PNPT1,RNF213,RTP4,SAMD9,STAT1,STAT2,TAP1,UBA7,UBE2L6,USP18,XAF1,ZNFX1 |

S15 Table. Continued.

|  | Bacteria infused day 16 | | Healthy day 15 | | Healthy day 16 | | Healthy day 17 | |
| --- | --- | --- | --- | --- | --- | --- | --- | --- |
| Predicted upstream regulator | +/-  z -score  *P* value | Molecules | +/-  z -score  *P* value | Molecules | +/-  z -score  *P* value | Molecules | +/-  z -score  *P* value | Molecules |
| NRAS | -  -2.62  2.80E-07 | GBP2,IFI16,IFIH1,IFIT1,LBP,PTX3,STAT1,USP18 | -  -2.21  3.19E-05 | IFI16,IFIH1,IFIT1,ISG15,STAT1,USP18 | -  -2.74  8.25E-09 | AGRN,B2M,CCND1,GBP2,IFI16,IFI35,IFIH1,IFIT1,ISG15,RNASE6,SLC66A3,STAT1,TAP1,USP18 | -  -2.18  2.95E-07 | CD86,EPAS1,IFI16,IFI35,IFIH1,ISG15,NCF2,PTX3,RNASE6,STAT1,TAP1,USP18 |
| P38 MAPK | +  2.40  1.40E-03 | BATF2,CCL8,GBP1,PML,STAT1,TNFSF10 | +  2.60  1.40E-04 | CCL8,CXCL10,GBP1,IRF7,PML,STAT1,TNFSF10 | +  2.87  7.01E-05 | BATF2,CCL11,CCND1,CD40,CXCL10,EDNRB,GBP1,IL23A,PLAUR,PML,SLC6A12,STAT1,TNFSF10 | +  2.77  8.90E-06 | ARG2,ATF3,CCL8,CCR7,CD40,CD69,CD86,CXCL10,DDC,GBP1,IRF7,S100A12,SCNN1A,STAT1 |
| PAF1 | +  2.45  1.19E-08 | DDX58,HERC5,IFI44,IFITM3,OAS2,ZNFX1 | +  2.45  7.72E-09 | DDX58,IFI44,IFI44L,ISG15,ISG20,ZNFX1 | +  3.16  2.97E-11 | HERC5,IDO1,IFI44L,IFIT3,ISG15,ISG20,OAS2,PLAUR,SERTAD1,ZNFX1 | +  2.83  1.27E-08 | DDX58,IDO1,IFI44,IFI44L,ISG15,ISG20,SERTAD1,ZNFX1 |
| PARP9 | +  2.22  2.61E-09 | IFI44,IFIT1,OAS2,SP110,STAT1 | +  2.21  1.81E-09 | IFI44,IFIT1,IRF7,ISG15,STAT1 | +  2.63  1.88E-10 | IFIT1,IFIT2,IFIT3,ISG15,OAS2,SP110,STAT1 | +  2.21  4.46E-07 | IFI44,IFIT2,IRF7,ISG15,STAT1 |
| PIK3CG | -  -2.24  2.47E-05 | GBP2,OAS2,STAT1,TNFSF10,ZBP1 | -  -2.45  8.23E-07 | CXCL10,GBP4,GBP5,STAT1,TNFSF10,ZBP1 | -  -3.29  5.73E-09 | B2M,C2,CXCL10,GBP2,GBP4,GBP6,OAS2,STAT1,TAP1,TNFSF10,ZBP1 | -  -2.65  5.07E-05 | C2,CXCL10,GBP4,GBP5,NLRC5,STAT1,TAP1 |
| PML | +  3.44  2.03E-13 | BST2,EPSTI1,HERC6,IFI27,IFI44,IFIH1,IFIT1,MX1,OAS1,OAS2,PML,STAT1 | +  3.99  3.50E-20 | BST2,EPSTI1,HERC6,IFI27,IFI44,IFI44L,IFIH1,IFIT1,IFITM1,IRF7,ISG15,ISG20,MX1,OAS1,PML,STAT1 | +  4.08  4.00E-13 | CCND1,EPSTI1,HERC6,IFI27,IFI35,IFI44L,IFIH1,IFIT1,IFIT3,ISG15,ISG20,MX1,OAS1,OAS2,PML,STAT1,TAP1 | +  3.59  3.61E-09 | IFI27,IFI35,IFI44,IFI44L,IFIH1,IFITM1,IRF7,ISG15,ISG20,MX1,OAS1,STAT1,TAP1 |

S15 Table. Continued.

|  | Bacteria infused day 16 | | Healthy day 15 | | Healthy day 16 | | Healthy day 17 | |
| --- | --- | --- | --- | --- | --- | --- | --- | --- |
| Predicted upstream regulator | +/-  z -score  *P* value | Molecules | +/-  z -score  *P* value | Molecules | +/-  z -score  *P* value | Molecules | +/-  z -score  *P* value | Molecules |
| PNPT1 | -  -3.97  1.17E-28 | CMPK2,DDX58,EIF2AK2,GBP2,IFI16,IFI44,IFIH1,OAS1,PARP12,PARP14,PARP9,RNF213,RTP4,STAT1,USP18,XAF1 | -  -4.22  6.26E-34 | CXCL10,DDX58,EIF2AK2,GBP4,IFI16,IFI44,IFIH1,IRF7,ISG15,OAS1,PARP12,PARP14,PARP9,RNF213,RTP4,STAT1,USP18,XAF1 | -  -4.77  8.39E-34 | AGRN,CMPK2,CXCL10,EIF2AK2,GBP2,GBP4,GBP6,IFI16,IFIH1,IFIT3,ISG15,LGALS3BP,OAS1,PARP12,PARP14,PARP9,RNF213,RTP4,STAT1,STAT2,UBE2L6,USP18,XAF1 | -  -4.56  2.17E-30 | CXCL10,DDX58,EIF2AK2,GBP4,IFI16,IFI44,IFIH1,IRF7,ISG15,LGALS3BP,OAS1,PARP12,PARP14,PARP9,RNF213,RTP4,STAT1,STAT2,UBE2L6,USP18,XAF1 |
| PRL | +  5.20  5.94E-33 | BST2,CMPK2,DDX58,DHX58,DTX3L,EIF2AK2,EPSTI1,HERC5,HERC6,IFI44,IFI6,IFIH1,IFIT1,IFIT5,IRF9,MLKL,MX2,OAS1,OAS2,PARP12,PARP14,RSAD2,SAMD9,SP110,STAT1,USP18,XAF1 | +  5.45  2.18E-39 | ADAR,BST2,CXCL10,DDX58,DHX58,DTX3L,EIF2AK2,EPSTI1,HERC6,IFI44,IFI44L,IFI6,IFIH1,IFIT1,IFIT5,IFITM1,IRF7,IRF9,ISG15,MX2,OAS1,PARP12,PARP14,PNPT1,RSAD2,SAMD9,SP100,STAT1,USP18,XAF1 | +  5.68  1.43E-40 | ADAR,B2M,CCND1,CD40,CMPK2,CXCL10,DTX3L,EIF2AK2,EPSTI1,HERC5,HERC6,IFI35,IFI44L,IFI6,IFIH1,IFIT1,IFIT3,IFIT5,IRF9,ISG15,LAMP3,LY6E,MLKL,MX2,OAS1,OAS2,PARM1,PARP10,PARP12,PARP14,PNPT1,PSME2,RSAD2,SHFL,SHISA5,SP110,STAT1,STAT2,TDRD7,TMEM140,TNFSF13B,TRIM25,USP18,XAF1 | +  5.46  1.42E-29 | ADAR,CD40,CD69,CXCL10,DDX58,DHX58,EIF2AK2,IFI35,IFI44,IFI44L,IFI6,IFIH1,IFIT5,IFITM1,IRF7,IRF9,ISG15,LY6E,MLKL,MX2,OAS1,PARP12,PARP14,PNPT1,RSAD2,SAMD9,SHISA5,SOCS1,STAT1,STAT2,TDRD7,TMEM140,TNFSF13B,USP18,XAF1 |

S15 Table. Continued.

|  | Bacteria infused day 16 | | Healthy day 15 | | Healthy day 16 | | Healthy day 17 | |
| --- | --- | --- | --- | --- | --- | --- | --- | --- |
| Predicted upstream regulator | +/-  z -score  *P* value | Molecules | +/-  z -score  *P* value | Molecules | +/-  z -score  *P* value | Molecules | +/-  z -score  *P* value | Molecules |
| PTGER4 | -  -3.57  2.42E-15 | CMPK2,DDX58,GBP2,HERC6,IFI16,IFIH1,PARP14,RNF213,RSAD2,RTP4,TNFSF10,USP18,XAF1 | -  -3.84  4.47E-19 | CXCL10,DDX58,GBP4,HERC6,IFI16,IFIH1,IRF7,ISG20,PARP14,RNF213,RSAD2,RTP4,TNFSF10,USP18,XAF1 | -  -3.59  3.43E-21 | CD40,CMPK2,CXCL10,GBP2,GBP4,GBP6,HERC6,IFI16,IFI35,IFIH1,IFIT2,IL23A,ISG20,PARP14,RNASEL,RNF213,RNF24,RSAD2,RTP4,TNFSF10,TRIM21,USP18,XAF1 | -  -4.32  8.56E-18 | CCR7,CD40,CD69,CXCL10,DDX58,GBP4,IFI16,IFI35,IFIH1,IFIT2,IRF7,ISG20,NCF2,PARP14,RNF213,RSAD2,RTP4,SHISA3,USP18,XAF1 |
| RC3H1 | -  -3.87  1.00E-23 | BST2,DDX58,IFI16,IFI27,IFI44,IFI6,IFIT1,IFITM3,IRF9,MX1,OAS1,OAS2,PARP9,RSAD2,STAT1 | -  -4.00  2.59E-26 | BST2,DDX58,IFI16,IFI27,IFI44,IFI44L,IFI6,IFIT1,IFITM1,IRF9,ISG15,MX1,OAS1,PARP9,RSAD2,STAT1 | -  -4.47  3.10E-24 | IFI16,IFI27,IFI44L,IFI6,IFIT1,IFIT2,IFIT3,IRF9,ISG15,MX1,OAS1,OAS2,PARP9,RSAD2,SHFL,STAT1,STAT2,TRIM21,TRIM25,TRIM56 | -  -4.00  7.85E-20 | CCR7,DDX58,IFI16,IFI27,IFI44,IFI44L,IFI6,IFIT2,IFITM1,IRF9,ISG15,MX1,OAS1,PARP9,RSAD2,STAT1,STAT2 |
| RNY3 | +  3.61  2.77E-26 | BATF2,EPSTI1,HERC5,IFI44,IFIT1,IFITM3,MX1,OAS1,OAS2,RSAD2,RTP4,SIGLEC1,XAF1 | +  3.46  3.50E-24 | CXCL10,EPSTI1,IFI44,IFI44L,IFIT1,ISG15,MX1,OAS1,RSAD2,RTP4,SIGLEC1,XAF1 | +  4.24  8.77E-31 | BATF2,CXCL10,EPSTI1,HERC5,HES4,IFI44L,IFIT1,IFIT3,ISG15,LAMP3,LY6E,MX1,OAS1,OAS2,RSAD2,RTP4,SIGLEC1,XAF1 | +  3.32  2.85E-16 | CXCL10,IFI44,IFI44L,ISG15,LY6E,MX1,OAS1,RSAD2,RTP4,SIGLEC1,XAF1 |
| SAMSN1 | +  2.00  2.21E-04 | CMPK2,PML,RSAD2,STAT1 | +  2.65  1.12E-08 | CXCL10,IRF7,ISG15,ISG20,PML,RSAD2,STAT1 | +  3.74  1.86E-13 | CD40,CMPK2,CXCL10,IFIT2,IFIT3,IL23A,ISG15,ISG20,PML,RSAD2,STAT1,STAT2,TRIM21,USP25 | +  3.32  6.27E-10 | CD40,CXCL10,IFIT2,IRF7,ISG15,ISG20,NMI,RSAD2,SOCS1,STAT1,STAT2 |
| SASH1 | +  2.00  8.45E-05 | CMPK2,PML,RSAD2,STAT1 | +  2.65  1.92E-09 | CXCL10,IRF7,ISG15,ISG20,PML,RSAD2,STAT1 | +  3.61  1.32E-13 | CD40,CMPK2,CXCL10,IFIT2,IFIT3,ISG15,ISG20,PML,RSAD2,STAT1,STAT2,TRIM21,USP25 | +  3.32  3.97E-11 | CD40,CXCL10,IFIT2,IRF7,ISG15,ISG20,NMI,RSAD2,SOCS1,STAT1,STAT2 |

S15 Table. Continued.

|  | Bacteria infused day 16 | | Healthy day 15 | | Healthy day 16 | | Healthy day 17 | |
| --- | --- | --- | --- | --- | --- | --- | --- | --- |
| Predicted upstream regulator | +/-  z -score  *P* value | Molecules | +/-  z -score  *P* value | Molecules | +/-  z -score  *P* value | Molecules | +/-  z -score  *P* value | Molecules |
| SIRT1 | -  -3.62  9.97E-16 | CMPK2,DDX58,DHX58,DKK1,IFI44,IFITM3,OAS1,OAS2,PARP12,PARP14,PML,RNF213,RSAD2,RTP4,SP110,STAT1,UBA7,USP18 | -  -3.34  8.21E-14 | ADAR,DDX58,DHX58,DKK1,IFI44,IRF7,OAS1,PARP12,PARP14,PML,RNF213,RSAD2,RTP4,STAT1,UBA7,USP18 | -  -3.84  1.31E-11 | ADAR,CCND1,CMPK2,DKK1,GBP6,IFIT3,LGALS3BP,LY6E,MAPT,OAS1,OAS2,PARP12,PARP14,PML,PRDM16,RNF213,RSAD2,RTP4,SP110,STAT1,TAP1,UBA7,USP18 | -  -4.03  7.30E-14 | ADAR,DDX58,DHX58,DKK1,EPAS1,IFI44,IRF7,LGALS3BP,LY6E,NEDD4L,NLRC5,OAS1,PARP12,PARP14,PCK2,RGS16,RNF213,RSAD2,RTP4,SCNN1A,STAT1,TACSTD2,TAP1,UBA7,USP18 |
| SOCS1 | -  -3.42  3.04E-14 | DDX58,IFI16,IFI27,IFI44,IFIH1,IFIT1,MX1,OAS1,OAS2,RSAD2,STAT1,USP18 | -  -3.82  2.61E-21 | CXCL10,DDX58,GBP5,IFI16,IFI27,IFI44,IFIH1,IFIT1,IRF7,ISG15,ISG20,MX1,OAS1,RSAD2,STAT1,USP18 | -  -3.76  1.01E-16 | CCND1,CD40,CXCL10,GBP6,H2-T24,IFI16,IFI27,IFIH1,IFIT1,IFIT2,IFIT3,ISG15,ISG20,MX1,OAS1,OAS2,RSAD2,STAT1,USP18 | -  -3.98  3.94E-21 | CCR7,CD40,CD69,CD86,CXCL10,DDX58,GBP5,H2-T24,IFI16,IFI27,IFI44,IFIH1,IFIT2,IRF7,ISG15,ISG20,MX1,OAS1,RSAD2,SOCS1,STAT1,USP18 |
| SP110 | -  -3.16  3.02E-11 | BST2,IFI27,IFI6,IFIH1,IFIT1,IFITM3,IRF9,MX1,OAS1,STAT1 | -  -3.32  4.47E-13 | BST2,CXCL10,IFI27,IFI6,IFIH1,IFIT1,IFITM1,IRF9,MX1,OAS1,STAT1 | -  -2.71  1.13E-07 | BCL2L12,CXCL10,IFI27,IFI6,IFIH1,IFIT1,IFIT3,IRF9,MX1,OAS1,STAT1 | -  -2.71  6.58E-08 | ATF3,BCL2L12,CXCL10,IFI27,IFI6,IFIH1,IFITM1,IRF9,MX1,OAS1,STAT1 |
| SPI1 | +  3.56  2.97E-13 | CMPK2,IFI27,IFI44,IFI6,IFIT1,IFITM3,IRF9,MX1,PARP12,PML,RSAD2,SP110,TNFSF10,USP18 | +  3.95  6.39E-18 | CXCL10,IFI27,IFI44,IFI44L,IFI6,IFIT1,IFITM1,IRF7,IRF9,ISG15,ISG20,MX1,PARP12,PML,RSAD2,TNFSF10,USP18 | +  4.53  3.20E-16 | C1QC,C3AR1,CCND1,CMPK2,CXCL10,IFI27,IFI44L,IFI6,IFIT1,IFIT2,IFIT3,IRF4,IRF9,ISG15,ISG20,LAMP3,LY6E,MX1,PARP12,PML,RSAD2,SP110,TNFSF10,USP18 | +  3.87  1.09E-11 | C1QC,CCR7,CXCL10,IFI27,IFI44,IFI44L,IFI6,IFIT2,IFITM1,IRF7,IRF9,ISG15,ISG20,LY6E,MX1,NCF2,PARP12,RSAD2,USP18 |

S15 Table. Continued.

|  | Bacteria infused day 16 | | Healthy day 15 | | Healthy day 16 | | Healthy day 17 | |
| --- | --- | --- | --- | --- | --- | --- | --- | --- |
| Predicted upstream regulator | +/-  z -score  *P* value | Molecules | +/-  z -score  *P* value | Molecules | +/-  z -score  *P* value | Molecules | +/-  z -score  *P* value | Molecules |
| STAT1 | +  4.56  4.49E-33 | BATF2,BST2,CMPK2,EIF2AK2,EPSTI1,GBP1,GBP2,HERC6,IFI16,IFI27,IFI44,IFI6,IFIH1,IFIT1,IFITM3,IRF9,MX1,OAS1,OAS2,PARP9,RNF213,RSAD2,RTP4,SP110,STAT1,TNFSF10,USP18,XAF1,ZBP1 | +  4.75  1.24E-37 | BST2,C4A/C4B,CXCL10,EIF2AK2,EPSTI1,GBP1,GBP4,GBP5,HERC6,IFI16,IFI27,IFI44,IFI44L,IFI6,IFIH1,IFIT1,IFITM1,IRF7,IRF9,ISG15,MX1,OAS1,PARP9,RNF213,RSAD2,RTP4,STAT1,TNFSF10,USP18,XAF1,ZBP1 | +  5.75  6.21E-46 | B2M,BATF2,C1S,C4A/C4B,CASP4,CASP8,CCND1,CD274,CD40,CMPK2,CXCL10,EIF2AK2,EPSTI1,FCGR1A,GBP1,GBP2,GBP4,GBP6,HERC6,IDO1,IFI16,IFI27,IFI35,IFI44L,IFI6,IFIH1,IFIT1,IFIT2,IFIT3,IL23A,IRF9,ISG15,LY6E,MX1,OAS1,OAS2,PARP9,PSME2,RNF213,RSAD2,RTP4,SOAT1,SP110,STAT1,STAT2,TAP1,TNFSF10,TNFSF13B,TRIM21,USP18,XAF1,ZBP1 | +  5.54  4.63E-34 | CALHM6,CASP4,CCR7,CD40,CD86,CFB,CREM,CXCL10,EIF2AK2,FCGR1A,GBP1,GBP4,GBP5,IDO1,IFI16,IFI27,IFI35,IFI44,IFI44L,IFI6,IFIH1,IFIT2,IFITM1,IRF7,IRF9,ISG15,LY6E,MX1,NLRC5,OAS1,PARP9,RNF213,RSAD2,RTP4,SOCS1,STAT1,STAT2,TAP1,TNFSF13B,USP18,WARS1,XAF1 |
| STAT4 | +  2.22  8.32E-04 | DDX58,IFIH1,PLAC8,STAT1,STC2 | +  2.63  5.29E-06 | CXCL10,DDX58,IFIH1,ISG15,ISG20,PLAC8,STAT1 | +  2.35  1.12E-05 | CXCL10,IFIH1,IFIT2,IRF4,ISG15,ISG20,MAP3K8,PLAC8,PRDM16,SERTAD1,STAT1 | +  3.29  6.77E-06 | ARHGAP15,CXCL10,DDX58,IFIH1,IFIT2,ISG15,ISG20,PLAC8,RGS16,SERTAD1,STAT1 |
| STING1 | +  2.20  3.37E-08 | IFI16,IFI44,IFITM3,OAS1,RSAD2,USP18 | +  2.93  9.79E-14 | CXCL10,GBP5,IFI16,IFI44,IRF7,ISG15,OAS1,RSAD2,USP18 | +  2.93  3.32E-09 | CGAS,CXCL10,IFI16,IFIT2,IFIT3,ISG15,OAS1,RSAD2,USP18 | +  3.52  3.12E-15 | CD86,CXCL10,GBP5,IFI16,IFI44,IFIT2,IRF7,ISG15,OAS1,PLEKHA4,RGS16,RSAD2,USP18 |
| TAB1 | -  -2.43  8.07E-10 | GBP1,GBP2,IFIH1,IFIT1,TNFSF10,XAF1 | -  -2.63  5.34E-12 | CXCL10,GBP1,IFIH1,IFIT1,IRF7,TNFSF10, XAF1 | -  -2.81  5.16E-10 | CXCL10,GBP1,GBP2,IFIH1,IFIT1,TNFSF10,TNFSF13B,XAF1 | -  -2.65  1.23E-08 | CFB,CXCL10,GBP1,IFIH1,IRF7,TNFSF13B,XAF1 |

S15 Table. Continued.

|  | Bacteria infused day 16 | | Healthy day 15 | | Healthy day 16 | | Healthy day 17 | |
| --- | --- | --- | --- | --- | --- | --- | --- | --- |
| Predicted upstream regulator | +/-  z -score  *P* value | Molecules | +/-  z -score  *P* value | Molecules | +/-  z -score  *P* value | Molecules | +/-  z -score  *P* value | Molecules |
| TGM2 | +  3.59  4.52E-13 | IFI6,IFIT1,IFIT5,IRF9,OAS1,OAS2,PARP14,PARP9,RNF213,SP110,STAT1,UBA7,XAF1 | +  3.05  1.71E-13 | CXCL10,IFI6,IFIT1,IFIT5,IRF9,OAS1,PARP14,PARP9,RNF213,SLC7A2,STAT1,UBA7,XAF1 | +  3.56  4.17E-17 | CCND1,CD93,CXCL10,IFI35,IFI6,IFIT1,IFIT2,IFIT3,IFIT5,IRF9,LGALS9,LY6E,OAS1,OAS2,PARP14,PARP9,PPP1R16B,RNF213,SP110,STAT1,TAP1,UBA7,XAF1 | +  3.29  2.20E-15 | CD86,CD93,CXCL10,IFI35,IFI6,IFIT2,IFIT5,IRF9,LGALS9,LY6E,NCF2,OAS1,PARP14,PARP9,PPP1R16B,RNF213,SLC16A1,STAT1,TAP1,UBA7,XAF1 |
| TICAM1 | +  2.41  7.46E-06 | CMPK2,DDX58,IFI16,IFIT1,RSAD2,TNFSF10 | +  3.09  1.79E-11 | CXCL10,DDX58,IFI16,IFIT1,IRF7,ISG15,ISG20,RSAD2,SLC7A2,TNFSF10 | +  3.77  8.56E-12 | CASP4,CD40,CMPK2,CXCL10,EDNRB,IFI16,IFIT1,IFIT2,IFIT3,IL23A,IRF3,ISG15,ISG20,RSAD2,TNFSF10 | +  3.51  6.75E-10 | CASP4,CD40,CD86,CFB,CXCL10,DDX58,IFI16,IFIT2,IRF7,ISG15,ISG20,RSAD2,SOCS1 |
| TLR3 | +  3.26  7.37E-24 | CMPK2,CPM,DDX58,DHX58,EIF2AK2,GBP2,HERC5,IFI16,IFI44,IFI6,IFIH1,IFIT1,MX1,MX2,OAS1,PTX3,RSAD2,STAT1,TNFSF10,USP18,ZNFX1 | +  3.09  3.08E-26 | CXCL10,DDX58,DHX58,EIF2AK2,GBP4,IFI16,IFI44,IFI44L,IFI6,IFIH1,IFIT1,IRF7,ISG15,ISG20,MX1,MX2,OAS1,RSAD2,STAT1,TNFSF10,USP18,ZNFX1 | +  4.15  1.16E-23 | CD274,CD40,CMPK2,CXCL10,EIF2AK2,GBP2,GBP4,HERC5,IFI16,IFI44L,IFI6,IFIH1,IFIT1,IFIT2,IFIT3,IL23A,IRF3,ISG15,ISG20,LIPA,MAP3K8,MX1,MX2,OAS1,RSAD2,STAT1,TNFSF10,TNFSF13B,USP18,ZNFX1 | +  4.14  1.35E-25 | ARG2,ATF3,CD40,CD69,CD86,CFB,CXCL10,DDX58,DHX58,EIF2AK2,GBP4,IFI16,IFI44,IFI44L,IFI6,IFIH1,IFIT2,IRF7,ISG15,ISG20,MX1,MX2,NMI,OAS1,PTX3,RSAD2,SOCS1,STAT1,TNFSF13B,USP18,ZNFX1 |

S15 Table. Continued.

|  | Bacteria infused day 16 | | Healthy day 15 | | Healthy day 16 | | Healthy day 17 | |
| --- | --- | --- | --- | --- | --- | --- | --- | --- |
| Predicted upstream regulator | +/-  z -score  *P* value | Molecules | +/-  z -score  *P* value | Molecules | +/-  z -score  *P* value | Molecules | +/-  z -score  *P* value | Molecules |
| TLR4 | +  2.75  4.32E-09 | BPI,CCL8,CMPK2,GBP2,IFI16,IFITM3,MX1,PML,PTX3,RSAD2,STAT1,TNFSF10 | +  2.56  2.28E-08 | CCL8,CXCL10,IFI16,IRF7,ISG15,ISG20,MX1,PML,RSAD2,STAT1,TNFSF10 | +  3.50  5.86E-14 | BPI,CASP8,CD274,CD40,CMPK2,CXCL10,GBP2,IFI16,IFIT2,IFIT3,IL23A,IRF3,ISG15,ISG20,MX1,OXTR,PML,RSAD2,SLC6A12,STAT1,STAT2,TNFSF10,TREM2,TRIM21,USP25 | +  3.51  1.42E-13 | ATF3,CCL8,CCR7,CD40,CD86,CFB,CXCL10,IFI16,IFIT2,IRF7,ISG15,ISG20,MX1,NMI,OXTR,PTX3,RGS16,RSAD2,SMPDL3B,SOCS1,SPTLC2,STAT1,STAT2,TNFRSF13B |
| TLR7 | +  2.81  2.09E-09 | DKK1,IFI44,IFIT1,IRF9,MX1,MX2,OAS2,PTX3,RSAD2,STAT1 | +  3.41  3.07E-15 | CXCL10,DKK1,IFI44,IFI44L,IFIT1,IFITM1,IRF7,IRF9,ISG15,ISG20,MX1,MX2,RSAD2,STAT1 | +  4.14  2.19E-15 | CCND1,CD274,CD40,CXCL10,DKK1,ESM1,IDO1,IFI35,IFI44L,IFIT1,IFIT3,IL23A,IRF9,ISG15,ISG20,MX1,MX2,OAS2,RSAD2,STAT1,STAT2 | +  4.46  3.73E-18 | ATF3,CCR7,CD40,CD69,CD86,CXCL10,DKK1,IDO1,IFI35,IFI44,IFI44L,IFITM1,IRF7,IRF9,ISG15,ISG20,MX1,MX2,PTX3,RSAD2,SOCS1,STAT1,STAT2 |
| TLR9 | +  3.15  3.86E-10 | CPM,IFI16,IFIT1,IRF9,MX1,MX2,OAS2,RSAD2,STAT1,TNFSF10,USP18 | +  3.65  5.81E-16 | CXCL10,IFI16,IFI44L,IFIT1,IFITM1,IRF7,IRF9,ISG15,ISG20,MX1,MX2,RSAD2,STAT1,TNFSF10,USP18 | +  4.30  7.35E-20 | CCND1,CD274,CD40,CXCL10,IDO1,IFI16,IFI35,IFI44L,IFIT1,IFIT2,IFIT3,IL23A,IRF4,IRF9,ISG15,ISG20,MX1,MX2,NAMPT,OAS2,RSAD2,STAT1,STAT2,TNFSF10,TNFSF13B,USP18 | +  4.98  4.32E-24 | ARG2,ATF3,CCR7,CD40,CD69,CD86,CXCL10,IDO1,IFI16,IFI35,IFI44L,IFIT2,IFITM1,IRF7,IRF9,ISG15,ISG20,MX1,MX2,PYCARD,RSAD2,SMPDL3B,SOCS1,SPTLC2,STAT1,STAT2,TNFRSF13B,TNFSF13B,USP18 |

S15 Table. Continued.

|  | Bacteria infused day 16 | | Healthy day 15 | | Healthy day 16 | | Healthy day 17 | |
| --- | --- | --- | --- | --- | --- | --- | --- | --- |
| Predicted upstream regulator | +/-  z -score  *P* value | Molecules | +/-  z -score  *P* value | Molecules | +/-  z -score  *P* value | Molecules | +/-  z -score  *P* value | Molecules |
| TNF | +  4.04  4.42E-10 | BST2,DDX58,DKK1,EIF2AK2,GBP1,GBP2,HERC5,IFI16,IFI27,IFI6,IFIH1,IFIT1,IFIT5,LBP,MX1,OAS1,OAS2,PARP14,PLAAT3,PML,PTX3,SAMD9,STAT1,TIFA,TNFSF10 | +  4.50  1.05E-11 | BST2,C4A/C4B,CXCL10,DDX58,DKK1,EIF2AK2,GBP1,GBP4,IFI16,IFI27,IFI6,IFIH1,IFIT1,IFIT5,IFITM1,IRF7,ISG15,MX1,OAS1,PARP14,PML,SAMD9,SLC7A2,STAT1,TIFA,TNFSF10 | +  5.35  9.79E-19 | ANXA1,B2M,C3AR1,C4A/C4B,CASP4,CASP8,CCL11,CCND1,CD274,CD40,CXCL10,DKK1,EDNRB,EIF2AK2,ESM1,FRMD4A,GBP1,GBP2,GBP4,GBP6,HERC5,IDO1,IFI16,IFI27,IFI6,IFIH1,IFIT1,IFIT3,IFIT5,IL23A,IRF4,ISG15,ITGA10,KYNU,LAMP3,LGALS9,MAP3K8,MST1R,MX1,NAMPT,OAS1,OAS2,OPTN,PARP14,PLAUR,PLVAP,PML,PSMA2,PSME2,SLC15A3,SLC40A1,SOAT1,STAT1,TAP1,TCIM,TDRD7,TIFA,TM4SF1,TNFSF10,TNFSF13B,TREM2,TRIM56 | +  4.75  3.67E-14 | AATK,ALOX5AP,ATF3,CASP4,CCR7,CD40,CD69,CD86,CFB,CNP,CREM,CRYAB,CXCL10,DDX58,DKK1,EIF2AK2,GBP1,GBP4,H19,IDO1,IFI16,IFI27,IFI6,IFIH1,IFIT5,IFITM1,IRF7,ISG15,LGALS9,MST1R,MSTN,MX1,NCF2,OAS1,OSMR,PARP14,PCK2,PIGR,PLVAP,PTX3,PYCARD,RGS16,S100A12,SAMD9,SCNN1A,SLC16A2,SOCS1,STAT1,TAP1,TDRD7,TIFA,TMEM40,TNFSF13B |
| TNFSF10 | +  2.78  1.32E-09 | EIF2AK2,IFI16,IFI27,IFI6,IFIT1,IRF9,STAT1,TNFSF10 | +  3.27  1.06E-14 | EIF2AK2,IFI16,IFI27,IFI6,IFIT1,IFITM1,IRF9,ISG15,SP100,STAT1,TNFSF10 | +  3.23  3.42E-09 | CASP8,EIF2AK2,IFI16,IFI27,IFI6,IFIT1,IRF9,ISG15,PSME2,STAT1,TNFSF10 | +  2.13  3.42E-07 | CD69,EIF2AK2,IFI16,IFI27,IFI6,IFITM1,IRF9,ISG15,STAT1 |
| TNK1 | +  2.00  2.36E-07 | IFI16,IFIH1,OAS2,TNFSF10 | +  2.24  1.31E-09 | IFI16,IFIH1,IRF7,ISG20,TNFSF10 | +  2.45  7.92E-09 | IFI16,IFIH1,IFIT2,ISG20,OAS2,TNFSF10 | +  2.24  3.25E-07 | IFI16,IFIH1,IFIT2,IRF7,ISG20 |

S15 Table. Continued.

|  | Bacteria infused day 16 | | Healthy day 15 | | | Healthy day 16 | | | Healthy day 17 | | |
| --- | --- | --- | --- | --- | --- | --- | --- | --- | --- | --- | --- |
| Predicted upstream regulator | +/-  z -score  *P* value | Molecules | +/-  z -score  *P* value | Molecules | +/-  z -score  *P* value | | Molecules | +/-  z -score  *P* value | | Molecules |  |
| TP53 | +  2.19  2.16E-02 | DKK1,GBP1,HERC5,IFI16,IRF9,MX1,OAS1,PLAAT3,PML,STAT1,TNFSF10,XAF1 | +  2.28  1.70E-03 | CXCL10,DKK1,FABP3,GBP1,IFI16,IRF7,IRF9,ISG15,MX1,OAS1,PML,STAT1,TNFSF10,XAF1 | +  2.66  5.92E-06 | | ANXA1,C1QC,C2,CASP4,CASP8,CCND1,COL13A1,COX7A1,CXCL10,DKK1,DRAM1,FAM83D,FRMD4A,GBP1,GDA,HERC5,IFI16,IFI35,IRF9,ISG15,LAMP3,MAP3K8,MX1,NAMPT,OAS1,P2RY14,PGAM2,PLAUR,PMEPA1,PML,POLK,PSMA2,PTPRE,SHISA5,SLC66A3,STAT1,TAP1,TNFSF10,XAF1 | +  3.01  1.25E-03 | | ATF3,C1QC,C2,CASP4,COL14A1,CRYAB,CXCL10,DKK1,DRAM1,EPAS1,FABP3,FGFBP1,GBP1,H19,IFI16,IFI35,IRF7,IRF9,ISG15,MX1,OAS1,PYCARD,RGS16,SCNN1A,SHISA5,SLC16A1,SPHK2,SPTLC2,STAT1,TAP1,XAF1 |  |
| TREX1 | -  -2.41  4.97E-11 | IFI16,IFI44,IFIT1,MX1,OAS1,USP18 | -  -3.09  1.10E-20 | CXCL10,IFI16,IFI44,IFI44L,IFIT1,ISG15,ISG20,MX1,OAS1,USP18 | -  -3.08  1.61E-15 | | CXCL10,IFI16,IFI44L,IFIT1,IFIT2,ISG15,ISG20,MX1,OAS1,USP18 | -  -3.07  8.10E-18 | | CD86,CXCL10,IFI16,IFI44,IFI44L,IFIT2,ISG15,ISG20,MX1,OAS1,USP18 |  |
| TRIM24 | -  -3.94  2.26E-22 | CMPK2,DDX58,DHX58,EPSTI1,GBP2,HERC6,IFI44,IFIH1,IRF9,OAS1,PARP12,PLAC8,RTP4,STAT1,UBA7,USP18 | -  -4.19  1.02E-26 | CXCL10,DDX58,DHX58,EPSTI1,GBP4,HERC6,IFI44,IFIH1,IRF7,IRF9,ISG15,OAS1,PARP12,PLAC8,RTP4,STAT1,UBA7,USP18 | -  -4.95  2.64E-27 | | AGRN,CMPK2,CXCL10,EPSTI1,GBP2,GBP4,HERC6,IFI35,IFIH1,IFIT2,IFIT3,IRF9,ISG15,LGALS3BP,MOV10,OAS1,PARP12,PLAC8,RTP4,SHISA5,STAT1,STAT2,TAP1,UBA7,USP18 | -  -4.95  6.64E-28 | | CALHM6,CXCL10,DDX58,DHX58,GBP4,IFI35,IFI44,IFIH1,IFIT2,IRF7,IRF9,ISG15,LGALS3BP,NMI,OAS1,PARP12,PLAC8,RTP4,SHISA5,SOCS1,STAT1,STAT2,TAP1,UBA7,USP18 |  |
| USP18 | -  -2.59  7.01E-12 | IFI6,IFIH1,IFITM3,IRF9,MX1,OAS1,TNFSF10 | -  -2.94  2.33E-16 | CXCL10,IFI6,IFIH1,IRF7,IRF9,ISG15,MX1,OAS1,TNFSF10 | -  -2.77  3.90E-10 | | CXCL10,IFI6,IFIH1,IRF9,ISG15,MX1,OAS1,TNFSF10 | -  -2.96  5.69E-12 | | CXCL10,IFI6,IFIH1,IRF7,IRF9,ISG15,MX1,OAS1,SOCS1 |  |

S15 Table. Continued.

|  | Bacteria infused day 16 | | Healthy day 15 | | Healthy day 16 | | Healthy day 17 | |
| --- | --- | --- | --- | --- | --- | --- | --- | --- |
| Predicted upstream regulator | +/-  z -score  *P* value | Molecules | +/-  z -score  *P* value | Molecules | +/-  z -score  *P* value | Molecules | +/-  z -score  *P* value | Molecules |
| VCAN | +  2.53  1.27E-10 | IFI44,IFI6,IFIT1,LBP,MX1,MX2,OAS2,PARP14,STAT1,XAF1 | +  2.71  2.21E-12 | C4A/C4B,IFI44,IFI44L,IFI6,IFIT1,IFITM1,MX1,MX2,PARP14,STAT1,XAF1 | +  2.51  5.95E-09 | AGRN,C1S,C4A/C4B,IFI44L,IFI6,IFIT1,IFIT2,MX1,MX2,OAS2,PARP14,STAT1,XAF1 | +  2.89  3.13E-08 | IFI44,IFI44L,IFI6,IFIT2,IFITM1,MX1,MX2,PARP14,PRELP,SMPDL3B,STAT1,XAF1 |
| ISG15 | -  -2.22  6.58E-12 | DDX58,IFI6,IFITM3,MX1,OAS1 |  |  |  |  |  |  |
| AIRE | -  -2.24  6.66E-06 | EIF2AK2,HERC6,IFI44,PARP14,TNFSF10 |  |  |  |  |  |  |
| PF4 | +  2.22  5.19E-09 | CMPK2,CPM,DDX58,EPSTI1,RSAD2,STAT1,USP18 |  |  |  |  |  |  |
| DUSP1 | +  2.00  5.58E-04 | CMPK2,DKK1,IFIT1,MX1 |  |  |  |  |  |  |
| NFKBIA | +  2.18  1.45E-02 | GBP2,IFI16,IFI6,PTX3,TNFSF10 |  |  |  |  |  |  |
| CNOT7 |  |  | -  -2.21  6.65E-21 | BST2,HERC6,IFI27,IFI44L,IFI6,IFIT5,IFITM1,ISG15,OAS1,PARP12,STAT1 | -  -2.22  5.43E-27 | B2M,CMPK2,HERC6,IFI27,IFI35,IFI44L,IFI6,IFIT5,ISG15,LGALS3BP,OAS1,OAS2,PARP12,SP110,STAT1,TAP1,UBE2L6 | -  -2.21  3.23E-19 | IFI27,IFI35,IFI44L,IFI6,IFIT5,IFITM1,ISG15,LGALS3BP,OAS1,PARP12,STAT1,TAP1,UBE2L6 |
| DNASE2 |  |  | -  -2.60  3.96E-18 | CXCL10,DHX58,IRF7,ISG15,OAS1,RSAD2,RTP4,TNFSF10,USP18,ZBP1 | -  -2.40  5.37E-13 | ACKR4,CXCL10,IFIT3,ISG15,OAS1,RSAD2,RTP4,TNFSF10,USP18,ZBP1 | -  -2.20  5.73E-10 | CXCL10,DHX58,IRF7,ISG15,OAS1,RSAD2,RTP4,USP18 |

S15 Table. Continued.

|  | Bacteria infused day 16 | | Healthy day 15 | | Healthy day 16 | | Healthy day 17 | |
| --- | --- | --- | --- | --- | --- | --- | --- | --- |
| Predicted upstream regulator | +/-  z -score  *P* value | Molecules | +/-  z -score  *P* value | Molecules | +/-  z -score  *P* value | Molecules | +/-  z -score  *P* value | Molecules |
| DOCK8 |  |  | +  2.45  6.31E-08 | CXCL10,IRF7,ISG15,ISG20,RSAD2,STAT1 | +  3.46  2.25E-12 | CD40,CMPK2,CXCL10,IFIT2,IFIT3,ISG15,ISG20,RSAD2,STAT1,STAT2,TRIM21,USP25 | +  3.32  2.87E-11 | CD40,CXCL10,IFIT2,IRF7,ISG15,ISG20,NMI,RSAD2,SOCS1,STAT1,STAT2 |
| IFI16 |  |  | +  2.21  3.88E-06 | CXCL10,DDX58,IFI16,ISG15,OAS1 | +  2.20  1.01E-04 | CCND1,CXCL10,IFI16,ISG15,OAS1,STAT2 | +  2.42  7.55E-05 | CXCL10,DDX58,IFI16,ISG15,OAS1,STAT2 |
| IFIH1 |  |  | +  2.40  1.91E-16 | CXCL10,IFI27,IFI44L,IFIT1,IRF7,ISG15,OAS1,RSAD2,SIGLEC1,USP18 | +  2.40  2.39E-11 | CXCL10,IFI27,IFI44L,IFIT1,ISG15,OAS1,OAS2,RSAD2,SIGLEC1,USP18 | +  2.20  4.14E-10 | CXCL10,IFI27,IFI44L,IRF7,ISG15,OAS1,RSAD2,SIGLEC1,USP18 |
| IFN alpha/ beta |  |  | +  2.39  1.66E-07 | CXCL10,IFI16,IRF7,RSAD2,STAT1,TNFSF10 | +  3.52  7.86E-13 | CD40,CXCL10,IDO1,IFI16,IFIT2,IFIT3,LY6E,RSAD2,STAT1,STAT2,TNFSF10,TNFSF13B,TRIM21 | +  3.79  6.20E-16 | CCR7,CD40,CD69,CD86,CXCL10,IDO1,IFI16,IFIT2,IRF7,LY6E,RSAD2,SOCS1,STAT1,STAT2,TNFSF13B |
| IFNL2 |  |  | +  2.24  6.40E-10 | CXCL10,IFIT1,IRF7,MX1,RSAD2 | +  2.00  1.01E-05 | CXCL10,IFIT1,MX1,RSAD2 | +  2.00  8.17E-06 | CXCL10,IRF7,MX1,RSAD2 |
| MAP2K3 |  |  | +  2.62  1.92E-09 | CCL8,CXCL10,IRF9,ISG15,PPA1,STAT1,TNFSF10 | +  2.43  5.97E-05 | CXCL10,IRF9,ISG15,PPA1,STAT1,TNFSF10 | +  2.62  3.61E-06 | ARG2,CCL8,CXCL10,IRF9,ISG15,PPA1,STAT1 |
| mir-155 |  |  | -  -2.21  3.18E-05 | CXCL10,IRF7,IRF9,MX1,STAT1 | -  -2.00  1.55E-04 | CCND1,CXCL10,IFIT3,IRF4,IRF9,MX1,STAT1 | -  -2.59  1.11E-04 | CD69,CXCL10,IRF7,IRF9,MX1,SOCS1,STAT1 |

S15 Table. Continued.

|  | Bacteria infused day 16 | | Healthy day 15 | | Healthy day 16 | | Healthy day 17 | |
| --- | --- | --- | --- | --- | --- | --- | --- | --- |
| Predicted upstream regulator | +/-  z -score  *P* value | Molecules | +/-  z -score  *P* value | Molecules | +/-  z -score  *P* value | Molecules | +/-  z -score  *P* value | Molecules |
| MYD88 |  |  | +  2.32  1.77E-04 | CXCL10,IRF7,ISG15,RSAD2,SLC7A2,USP18 | +  3.39  1.31E-05 | CASP4,CD274,CD40,CMPK2,CXCL10,EDNRB,IFIT2,IL23A,ISG15,RSAD2,TNFSF13B,USP18 | +  3.32  7.62E-06 | CASP4,CD40,CD86,CXCL10,IFIT2,IRF7,ISG15,RSAD2,SOCS1,TNFRSF13B,TNFSF13B,USP18 |
| OSM |  |  | +  2.71  2.94E-04 | CXCL10,GBP1,IRF7,IRF9,ISG20,MX1,OAS1,STAT1 | +  4.10  1.18E-07 | ANXA1,B2M,BTC,C1S,CASP4,CCL11,CCND1,CXCL10,GBP1,GBP2,IFI35,IRF9,ISG20,KRT17,MX1,NAMPT,OAS1,STAT1,TAP1,TM4SF1,UBE2L6 | +  3.50  9.54E-09 | ATF3,CASP4,CXCL10,GBP1,IFI35,IRF7,IRF9,ISG20,KRT17,LY6G6C,MX1,NEDD4L,OAS1,OSMR,PIGR,S100A12,SLC15A1,SLC16A1,SOCS1,STAT1,TAP1,UBE2L6 |
| PRDM16 |  |  | -  -2.18  1.11E-09 | CXCL10,GBP4,IFI44,IRF7,MX2,STAT1 | -  -2.40  4.27E-08 | CXCL10,GBP4,IFIT2,MX2,OAS2,STAT1,STAT2 | -  -2.58  9.41E-10 | CXCL10,GBP4,IFI44,IFIT2,IRF7,MX2,STAT1,STAT2 |
| SOCS3 |  |  | -  -2.21  9.23E-06 | CXCL10,IFIT1,ISG20,MX1,OAS1 | -  -2.89  1.40E-09 | CCND1,CD40,CXCL10,FCGR1A,IFIT1,IFIT2,IL23A,ISG20,MX1,OAS1,OAS2 | -  -2.37  1.22E-08 | ATF3,CD40,CD86,CXCL10,FCGR1A,IFIT2,ISG20,MX1,OAS1,SOCS1 |
| STAT2 |  |  | +  2.01  5.80E-30 | CXCL10,GBP1,IFI27,IFI6,IFIT1,IFITM1,IRF7,IRF9,ISG15,MX1,OAS1,RSAD2,RTP4,STAT1,TNFSF10,USP18,ZBP1 | +  2.39  7.64E-28 | CD40,CXCL10,GBP1,GBP6,IFI27,IFI35,IFI6,IFIT1,IFIT2,IFIT3,IRF9,ISG15,MX1,OAS1,OAS2,RSAD2,RTP4,STAT1,TNFSF10,USP18,ZBP1 | +  2.80  1.52E-26 | CD40,CD86,CXCL10,GBP1,IFI27,IFI35,IFI6,IFIT2,IFITM1,IRF7,IRF9,ISG15,MX1,OAS1,RSAD2,RTP4,SOCS1,STAT1,USP18,WARS1 |
